# TMSCF_3_-Mediated Conversion of Salicylates into α,α-Difluoro-3-coumaranones: Chain Kinetics, Anion-Speciation, and Mechanism

**DOI:** 10.1021/acs.joc.3c02219

**Published:** 2023-12-02

**Authors:** Hannah B. Minshull, Guy C. Lloyd-Jones

**Affiliations:** School of Chemistry, University of Edinburgh, Joseph Black Building, Edinburgh EH9 3FJ, U.K.

## Abstract

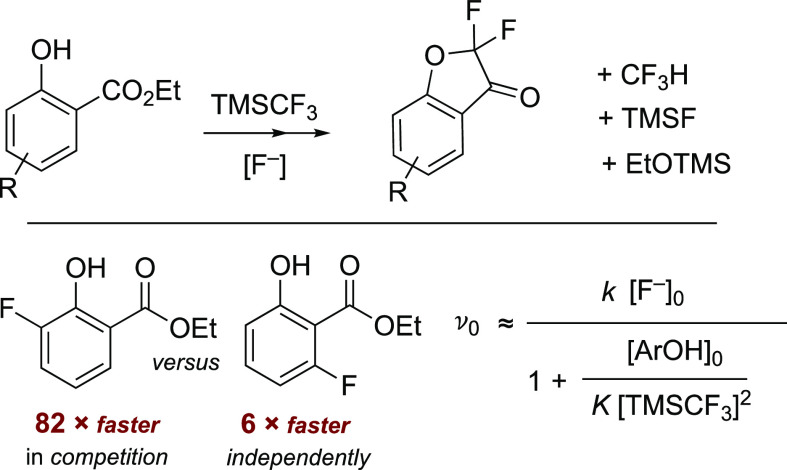

As reported by Zhao, the TBAT ([Ph_3_SiF_2_]^−^[Bu_4_N]^+^)-initiated reaction of ethyl salicylate with TMSCF_3_ in THF generates α,α-difluoro-3-coumaranones via the corresponding *O*-silylated ethoxy ketals. The mechanism has been investigated by in situ ^19^F and ^29^Si NMR spectroscopy, CF_2_-trapping, competition, titration, and comparison of the kinetics with the 3-, 4-, 5-, and 6-fluoro ethyl salicylate analogues and their *O*-silylated derivatives. The process evolves in five distinct stages, each arising from a discrete array of anion speciations that modulate a sequence of silyl-transfer chain reactions. The deconvolution of coupled equilibria between salicylate, [CF_3_]^−^, and siliconate [Me_3_Si(CF_3_)_2_]^−^ anions allowed the development of a kinetic model that accounts for the first three stages. The model provides valuable practical insights. For example, it explains how the initial concentrations of the TMSCF_3_ and salicylate and the location of electron-withdrawing salicylate ring substituents profoundly impact the overall viability of the process, how stoichiometric CF_3_H generation can be bypassed by using the *O*-silylated salicylate, and how the very slow liberation of the α,α-difluoro-3-coumaranone can be rapidly accelerated by evaporative or aqueous workup.

## 1 Introduction

### 1.1 Trifluoromethyltrimethylsilane

**Scheme 1:**
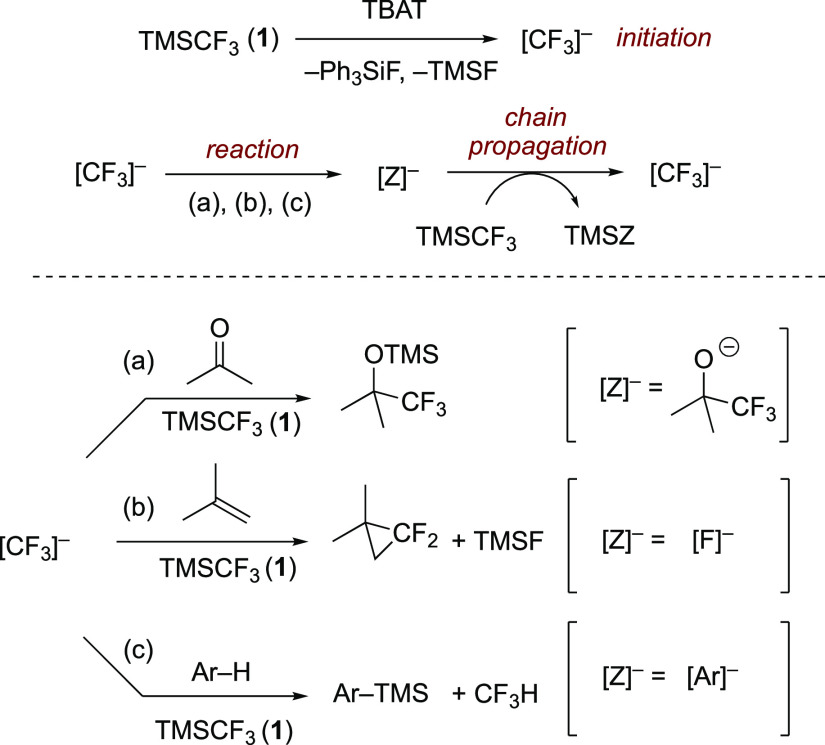
Generic TMSCF_3_-Mediated Chain Reactions TBAT = [Ph_3_SiF_2_]^−^[Bu_4_N]^+^; TMSF = Me_3_SiF.

Since its introduction in 1984, TMSCF_3_ (**1**) has been a reagent of choice for the addition of CF_3_ to electrophiles.^1,2^ Over the past decade, the scope of application of **1** has expanded considerably and now includes, for example, the transfer of CF_2_ and C–H silylation.^1−6^ The majority of the reactions of TMSCF_3_ (**1**) require the addition of a silaphilic anion, and a growing body of evidence indicates that this liberates a transient trifluoromethylcarbanion(oid) “[CF_3_]^−^” from the TMS group.^2,3^ Through kinetic and NMR spectroscopic studies, we recently established that reactions of TMSCF_3_ initiated by [Ph_3_SiF_2_]^−^[Bu_4_N]^+^ (“TBAT”), a readily handled surrogate for “[F]^−^”, proceed via anionic chain reactions, Scheme 1.^3^

In these reactions, the identity of the chain carrier, [Z]^−^, is determined by whether the [CF_3_]^−^ reacts with an electrophile, e.g., a ketone, Scheme 1a, a fluorophile, e.g., a TMS group, Scheme 1b, or an acid, e.g., an arene bearing suitably electron-withdrawing substituents, Scheme 1c. For an efficient process, the chain carrier, [Z]^−^, must be stable enough to be generated but also sufficiently silaphilic to react with TMSCF_3_ to regenerate [CF_3_]^−^ and propagate the chain. Examples of efficient chain carriers are [Z]^−^ = [RO]^−^, [F]^−^, and [R]^−^. Conversely, if [Z]^−^ does not react efficiently with TMSCF_3_, for kinetic or thermodynamic reasons, the chain is terminated, and a stoichiometric initiator will be required. Examples of this are for [Z]^−^ = [Cl]^−^ or [RS]^−^.

The identity of which of the step(s) governs the chain reaction velocity and thus the kinetic influence of [CF_3_]^−^ and [Z]^−^ is highly substrate dependent. Consequently, the rate of the overall process is dictated by (i) the initiator concentration, [TBAT]_0_, (ii) the carrier speciation, i.e., the dynamic distribution of [CF_3_]^−^ and [Z]^−^, and (iii) the presence of endogenous or exogenous inhibitors, or chain terminators, that reduce the net carrier concentration [CF_3_ + Z]^−^. Taken together, these factors can lead to unusual temporal–concentration profiles, including delayed or progressive rate accelerations. Thus, in the absence of kinetic insight into the species controlling the rate and evolution of the process, scale-up of reactions using TMSCF_3_ (**1**) should be conducted with caution.

### 1.2 Synthesis of Difluoro-3-coumaranones

**Scheme 2:**
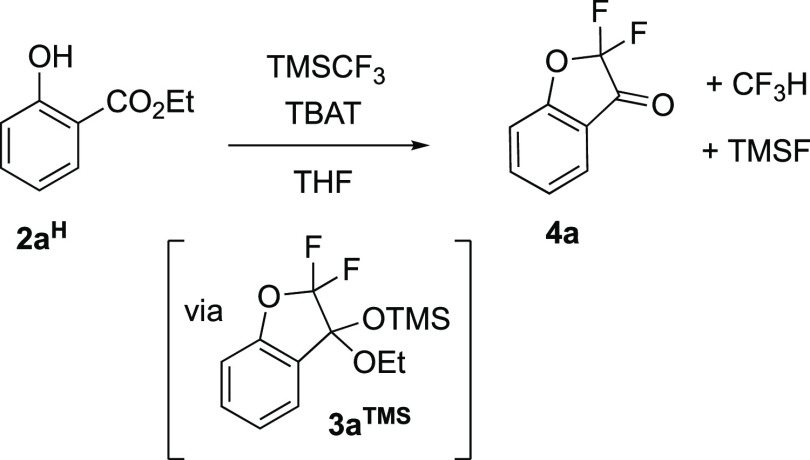
Zhao’s α,α-Difluoro-3-coumaranone Synthesis Conditions: **2a**^**H**^ 0.1 M, TMSCF_3_ (**1**) 0.25 M, TBAT, 0.03 M, 0.3 equiv, THF, N_2_, RT. TBAT = [Ph_3_SiF_2_]^−^[Bu_4_N]^+^.

There is a growing interest in annelation reactions proceeding via the formal insertion of CF_2_,^6^ and in 2021, Zhao reported using TMSCF_3_ (**1**) and TBAT in THF to convert salicylate esters (**2**^**H**^) into α,α-difluoro-3-coumaranones (**4**), Scheme 2.^5^ As part of these developments, Zhao identified that the TMSCF_3_ reagent (**1**) plays a “multifunctional role”,^5^ and that an *O*-silylated ethoxy ketal (**3**^**TMS**^, Scheme 2) is an intermediate that accumulates before being converted into coumaranone (**4**). Zhao’s method^5^ provides a broad range of new α,α-difluoro compound **4** as prospective bioisosteres to the potent range of 3-coumaranones employed in drug discovery^7^ and is thus of considerable interest. However, the complexity of the process evident from Zhao’s preliminary investigations^5^ warrants a more detailed analysis of the chain carrier speciation and sequence of steps that convert salicylates (**2**^**H**^) into coumaranones (**4**).^8^

**Chart 1:**
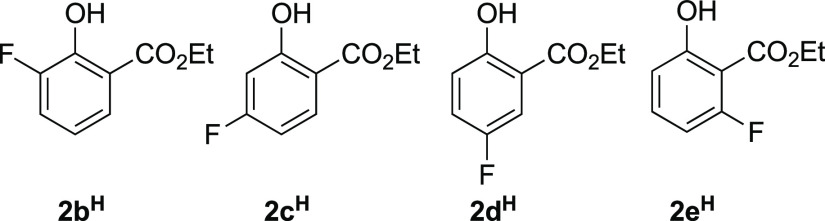
Monofluoro-Salicylates **2b–e**^**H**^

Herein, we report on the in situ ^19^F and ^29^Si NMR spectroscopic analysis of the TBAT-initiated reaction of ethyl salicylate (**2a**^**H**^, Scheme 2) and its 3-, 4-, 5-, and 6-fluoroarene analogues (**2b–e**^**H**^, Chart 1) with TMSCF_3_ (**1**) in THF. While the study confirms several aspects of the prior mechanistic proposals,^5^ it also elucidates and explains several anomalies,^8^ as well as providing a general kinetic model for the absolute and relative rates of evolution of the ketals (**3**^**TMS**^) from the salicylates (**2**). The evolution of ketone **4** from ketal **3**^**TMS**^ was found to vary extensively between experiments, and alternative methods to stimulate this process (**3**^**TMS**^**→ 4**) are also presented.

## 2 Results and Discussion

### 2.1 Preliminary Observations

We began by identifying conditions under which the conversion of ethyl salicylates **2a–e**^**H**^ to coumaranones **4a**–**e** could be effectively and safely monitored in situ by ^19^F NMR spectroscopy.^9^ This required several further adjustments to the preparative methodology reported by Zhao.^5^ First, in addition to reducing the scale of the process from 5 to 0.5 mL, the concentration of the TBAT was reduced from 110 to 15 mM so that the full sequence of steps in the overall reaction could be analyzed in detail over a suitable time frame. Second, the concentration of the salicylate **2a–e**^**H**^ was reduced to avoid the development of hazardous overpressures of fluoroform (CF_3_H) in the sealed NMR tubes.

**Figure 1:**
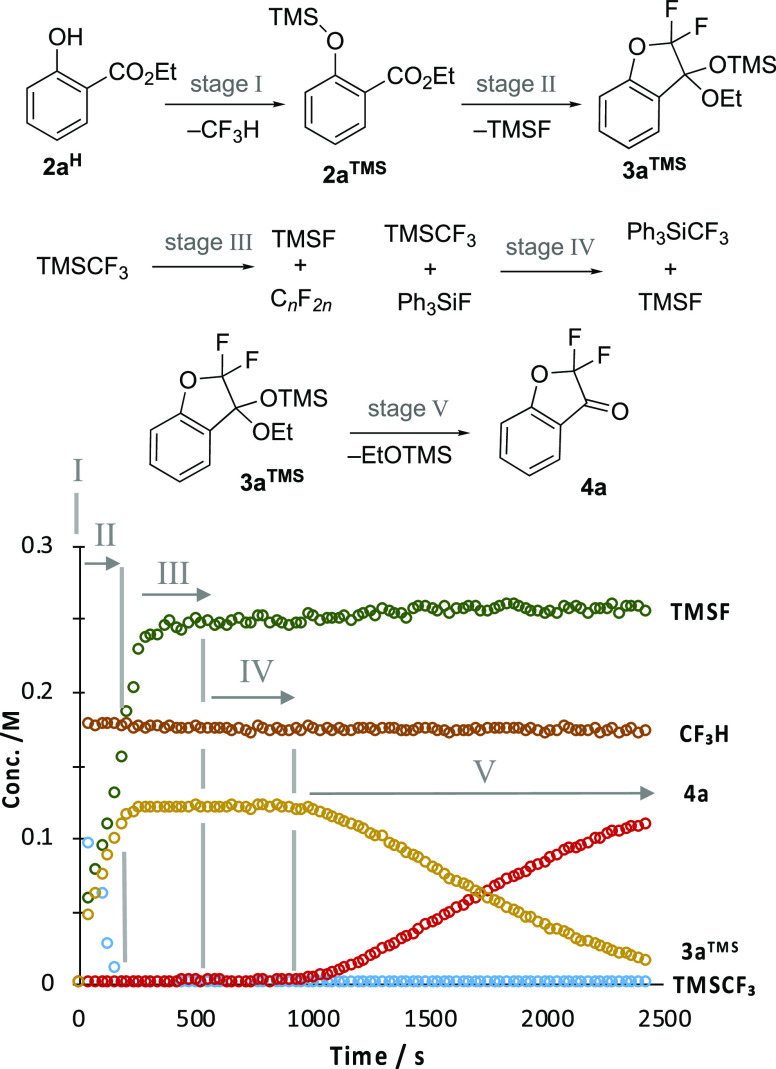
Five stages (I–V) identified by in situ ^19^F NMR (376 MHz) spectroscopic analysis of the conversion of salicylate **2a**^**H**^ into coumaranone **4a**. Conditions: **2a**^**H**^ 0.18 M, TMSCF_3_ (1) 0.49 M, TBAT, 0.015 M, 0.08 equiv, THF, N_2_, RT. TBAT = [Ph_3_SiF_2_]^−^[Bu_4_N]^+^. Stage I is complete within the “dead-time” between the addition of the TBAT and the measurement of the first NMR spectrum.

Single pulse ^19^F NMR spectra, acquired at 15 s intervals after the addition of TBAT (0.075 equiv) to a solution of the parent salicylate **2a**^**H**^ (0.2 M) and TMSCF_3_ (0.5 M) in THF, identified that the overall process evolves in five distinct stages, beginning with CF_3_H generation (stage I, Figure 1). The ketal, **3a**^**TMS**^, is generated in stage II in concert with trimethylsilyl fluoride, TMSF. This process diverges in stage III, with significant acceleration in the generation of TMSF and a cessation in ketal **3a**^**TMS**^ production. After a short period, TMSF generation then ceases abruptly, and the process enters stage IV. This sustained period of apparent stasis eventually leads to the conversion of ketal **3a**^**TMS**^ into ketone **4a** in stage V.

Analogous behavior was found for the aryl-fluorinated salicylates (**2b–e**^**H**^, Chart 1) that were explored under these standard conditions ([**1**]_0_ 0.5 M; [**2a–e**^**H**^]_0_ 0.2 M; and [TBAT]_0_ 0.015 M), but in some cases, the induction period (stage IV) lasted many hours, and in others there were competing side reactions in stages II and III, see Section 2.7.

### 2.2 Stage I: Aryl *O*-Silylation

Fluoroform (CF_3_H) is generated immediately after the addition of TBAT to a mixture of TMSCF_3_ + **2a**^**H**^ and before any significant accumulation of the ketal, **3a**^**TMS**^. Quantitative ^19^F NMR spectroscopy shows that the amount of CF_3_H generated corresponds to the initial concentration of the salicylate, i.e., [CF_3_H]_tot_ = [**2a**^**H**^]_0_. Analysis of the fluorinated substrates (**2b**–**e**^**H**^) identified that the salicylate is completely consumed in stage I to generate the corresponding aryl-*O*-silyl ether, **2b**–**e**^**TMS**^. The analogous reaction of the sterically more hindered reagent TESCF_3_ with **2e**^**H**^ proceeded slowly enough for the parallel evolution of equimolar CF_3_H and the aryl-*O*-triethylsilane (**2e**^**TES**^) in stage I to be monitored by in situ ^19^F NMR, see Section S4.2 in the Supporting Information.

The identities of the aryl-*O*-trimethylsilyl ethers, **2a–e**^**TMS**^, were confirmed by independent synthesis, see Section S3.2 in the Supporting Information. Importantly, the silyl ethers undergo conversion to the corresponding ketals, **3a–e**^**TMS**^, and ketones, **4a–e**, when reacted with TMSCF_3_ and TBAT. They do this without generating CF_3_H, thus bypassing stage I to directly enter stage II. A key observation in all the reactions, beginning from the phenolic (**2a–e**^**H**^) or silylated (**2a–e**^**TMS**^) forms of the salicylate, is that the ^19^F NMR signals arising from fluoroaryl substituents on the silylated substrates (**2b–e**^**TMS**^) undergo a progressive increase in line width and decrease in chemical shift during stages II and III. ^19^F NMR analysis of the titration of the 5-fluoroarene silyl ether **2d**^**TMS**^ with TBAT, in the absence of TMSCF_3_, shows that the process generates equimolar TMSF, salicylate anion, [**2d**]^−^, and Ph_3_SiF, see Section S3.4 in the Supporting Information.^10^ There is a significant line-broadening and progressive migration in the chemical shifts of all of the species, except the Ph_3_SiF, through the titration.

**Scheme 3:**
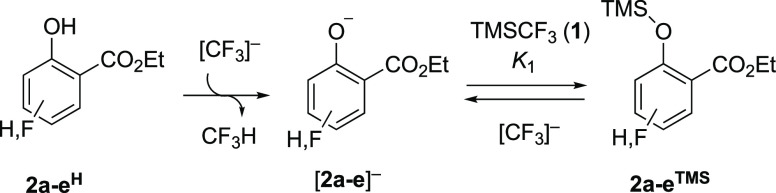
Stage I Silylation and Anionic Equilibrium, *K*_1_ Equilibrium, *K*_1_, between silyl ethers **2a–e**^**TMS**^ and salicylate anions, [**2a**–**e**]^−^, is rapid relative to the ^19^F NMR (376 MHz) time scale.

Overall, the data indicate that there is a dynamic equilibrium between the silyl ethers **2a–e**^**TMS**^ and their salicylate anions, [**2a–e**]^−^, with the rate, *k*_1_, and speciation, *K*_1_, dependent on the aryl ring substituents (H, F), the concentration of TMSCF_3_ (**1**), and the total anion concentration, [TBAT]_0_, Scheme 3.

Under the standard conditions of the reaction, the exchange, *k*_1_ and *k*_–1_, between **2b–e**^**TMS**^ and [**2b**–**e**]^−^ is fast enough to coalesce their ^19^F NMR resonances into a broad time-averaged, concentration-weighted peak. At the start of the reaction, the speciation is dominated by **2**^**TMS**^, and as the reaction progresses and both **2b–e**^**TMS**^ and TMSCF_3_ are consumed, the weighted chemical shift of the signal migrates upfield toward that of [**2b**–**e**]^−^, see Section S3.1 in the Supporting Information.^10^

### 2.3 Stage II: Ketal **3**^**TMS**^ Generation

**Scheme 4:**
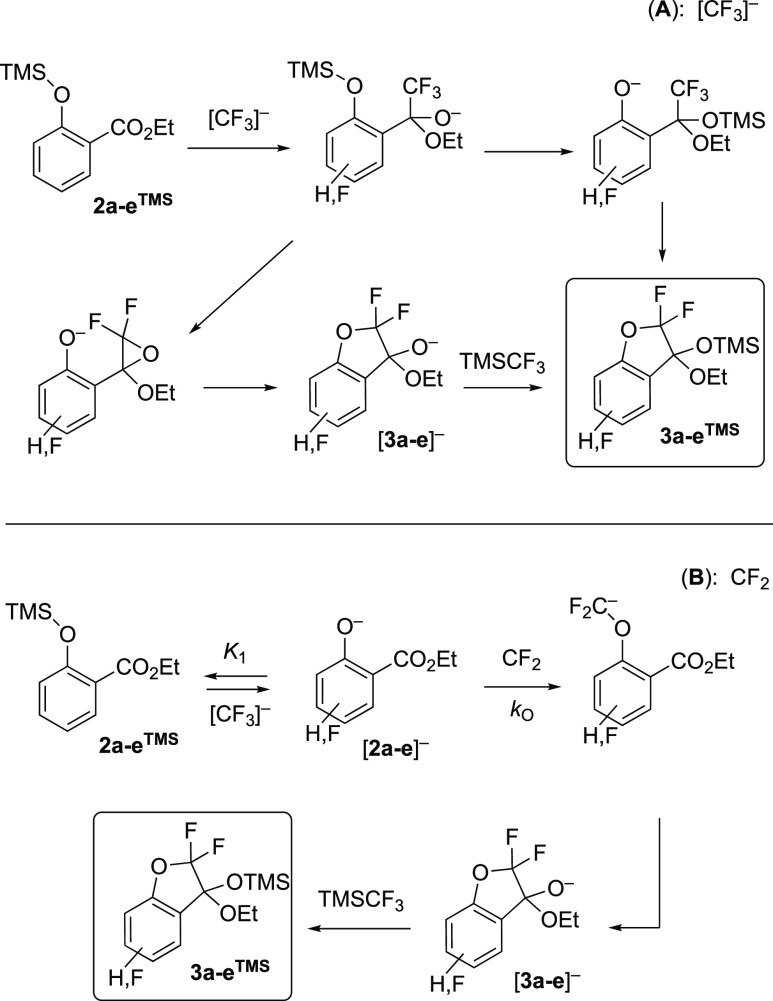
Potential Routes “A” and “B” from Silyl Ether 2**a–e**^TMS^ to Ketal 3**a–e**^TMS^, in Stage II Various alternative related silyl transfer(s) and intramolecular displacement(s) can be envisaged for route A.

Two distinct general pathways (A and B) can be envisaged for the process that converts **2a–e**^**TMS**^ to **3a–e**^**TMS**^, Scheme 4. One begins by nucleophilic attack of the ester carbonyl in **2a–e**^**TMS**^ by [CF_3_]^−^, followed by various silyl transfer(s) and intramolecular displacement(s).^11^ The second general pathway begins with equilibrium between the silyl ether **2a–e**^**TMS**^ and the salicylate anion, [**2a**–**e**]^−^ (1/*K*_1_), with the latter being trapped by CF_2_.^8^ The ester then undergoes intramolecular nucleophilic attack by [–OCF_2_]^−^, and the resulting ketal oxy-anion [**3a**–**e**]^−^ is silylated by TMSCF_3_. Both pathways require [CF_3_]^−^ and both pathways generate TMSF in a 1:1 ratio with ketal **3a–e**^**TMS**^ during stage II, Figure 1.

**Scheme 5:**
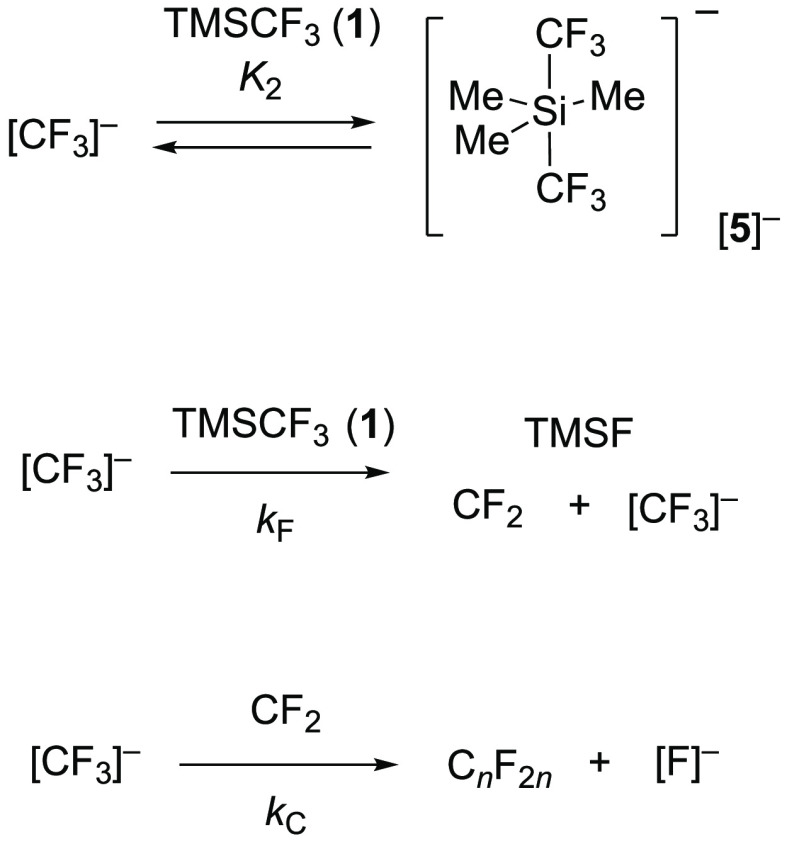
Equilibrium (*K*_2_) between [CF_3_]^−^ and Siliconate [5]^−^, Carbene Liberation (*k*_F_), and Oligomerization (*k*_C_)

The pentacoordinate siliconate [**5**]^−^, Scheme 5, is a key indicator for trace concentrations of the carbanion(oid), [CF_3_]^−^[Bu_4_N]^+^, in a medium containing TMSCF_3_ (**1**).^2c,2d,3^ The siliconate does not react directly with electrophiles but instead acts as a dynamic and dominant anion reservoir (*K*_2_) to a metastable (*k*_F_ and *k*_C_) system.^3^ The rates of reactions of carbonyl species with TMSCF_3_ (**1**) [ are powerfully attenuated by this equilibrium and thus accelerate with conversion when there is a substoichiometric TMSCF_3_ reagent: [**1**]_0_/[carbonyl]_0_ < 1.^3a^ Conversely, rate-limiting generation of CF_2_ by reaction of [CF_3_]^−^ with TMSCF_3_ (**1**), Scheme 5, is not significantly influenced by the siliconate equilibrium (*K*_2_), due to the near-cancelation of the effects of [TMSCF_3_] (**1**) concentration on the competing steps (*K*_2_, *k*_F_).^3b^ However, both processes (*K*_2_ and *k*_F_) are sensitive to the steric hindrance of the silyl reagent. For example, using TESCF_3_ leads to higher [CF_3_]^−^ concentrations (*K*_2_^TMS^/*K*_2_^TES^ ≈ 20) and overall faster CF_3_-addition to carbonyl species, but slower generation of CF_2_.^3^

**Figure 2:**
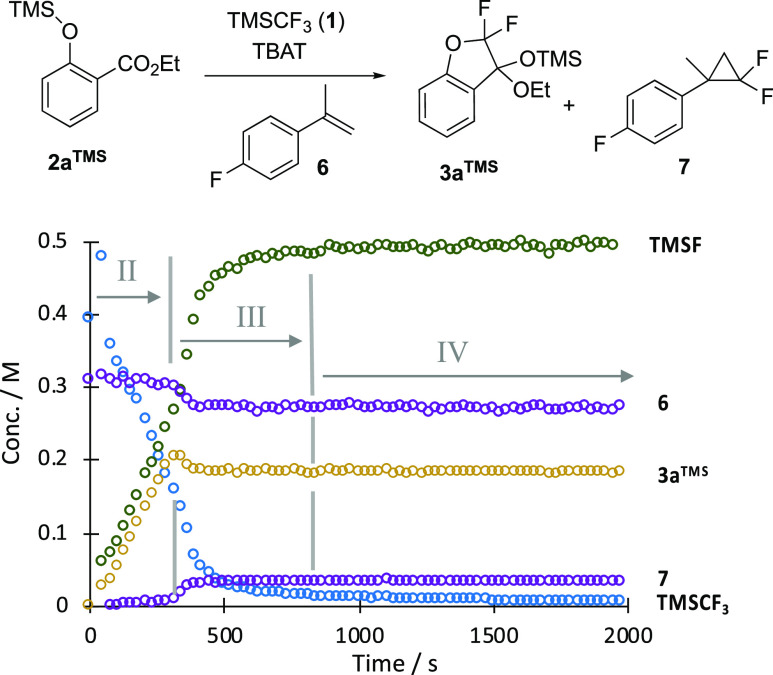
Use of alkene **6** as a probe for CF_2_ generation in stages II and III. Conditions: **2a**^TMS^, 0.2 M, TMSCF_3_, **1**, 0.5 M, TBAT, 0.015 M, 0.08 equiv, 6, 0.3 M, THF, N_2_, RT. TBAT = [Ph_3_SiF_2_]^−^[Bu_4_N]^+^. Analysis by in situ ^19^F NMR (376 MHz).

The above features inform tests to probe and distinguish the two general pathways of [CF_3_]^−^ addition (A) versus CF_2_ addition (B), Scheme 4. The presence of [**5**]^−^, and by implication [CF_3_]^−^,^3^ was confirmed by the appearance of a broad signal at δ_F_ ≈ −64.2 ppm^3^ during stage II of the in situ ^19^F NMR analysis of the conversion of silyl ether **2a**^**TMS**^ to ketal **3a**^**TMS**^ on cooling the sample to 275 K, see Section S1.4 in the Supporting Information. Moreover, on using TESCF_3_, the rate of generation of ketals **3c,d,e**^**TES**^ is strongly suppressed compared to identical conditions when employing TMSCF_3_ (**1**), see Section S1.3 in the Supporting Information. These results weigh strongly against a mechanism in which the silyl ethers **2a–e**^**R3Si**^ undergo nucleophilic attack by [CF_3_]^−^, i.e., pathway A in Scheme 4.^3a^ The addition of α-methyl-*p*-fluorostyrene (**6**) to the reaction of preformed silyl ether **2a**^**TMS**^ resulted in no detectable attenuation in the rate of generation of ketal **3a**^**TMS**^ but did produce a small quantity of difluorocyclopropane **7**, the product of the addition of the electrophilic carbene CF_2_ to the alkene,^3b,12^Figure 2.

The majority of **7** is generated at the point of transition from stage II to III, when **2a**^**TMS**^ is near fully consumed and the concentration of salicylate [**2**]^−^ becomes acutely reduced (*K*_1_, Scheme 3). This behavior is consistent with stage II proceeding via rate-limiting generation (*k*_F_) of CF_2_, Scheme 5, and then rapid trapping (*k*_O_) of this by the salicylate anion [**2a**]^−^ to generate [**3a**]^−^ (B), Scheme 4;^8,13^ see Section 2.8 for discussion of the overall anion speciation and kinetics in stage II.

### 2.4 Stage III: Accelerating TMSF Generation

**Scheme 6:**
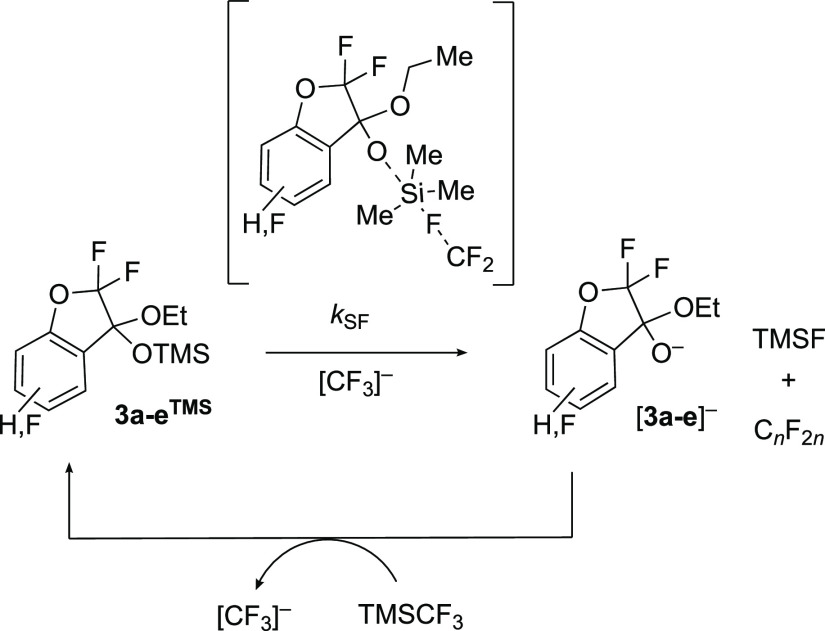
Accelerating TMSF Generation in Stage III^14^ via Ketals **3a–e**^TMS^ Acting as Surrogate F-Acceptors (*k*_SF_)

The primary process in stage III is the conversion of excess TMSCF_3_ into TMSF and perfluoroalkenes, C_*n*_F_2*n*_. The latter are evident from the broad and complex array of multiplets that accumulate in the in situ ^19^F NMR spectra, see Section S1.3 in the Supporting Information. We have previously shown that the hierarchical growth of C_*n*_F_2*n*_ species in difluorocyclopropanation reactions^12^ involving TMSCF_3_ arises from a cascade of formal CF_2_ oligomerizations initiated by addition (*k*_C_) of [CF_3_]^−^ and elimination of fluoride, Scheme 5.^3b,3d^ The process can be monitored by the accumulating TMSF coproduct, and in all previous cases, it has been found to undergo progressive deceleration due to anion sequestration in larger oligomers to generate perfluorocarbanions such as [C_11_F_23_]^−^.^2e,3b,3d^ In stark contrast, during stage III of the reactions of TMSCF_3_ (**1**) with salicylates, TMSF generation accelerates, and in some cases, profoundly, vide infra. The magnitude of the acceleration is dependent on the identity of the ketal **3**^**TMS**^, suggesting the latter can act as a surrogate fluoride acceptor (*k*_SF_) to accelerate the overall conversion of TMSCF_3_ into TMSF and C_*n*_F_2*n*_,^14^Scheme 6.

The anionic chain reaction then propagates via resilylation of ketal oxy-anion [**3**]^−^ by TMSCF_3_. Transient dynamic line broadening of the diastereotopic fluorine atoms in the α,α-difluoro unit in **3e**^**TMS**^ during stage III and an accompanying contraction in their ^19^F NMR chemical shift separation, see Section S5.1 in the Supporting Information, supports the conclusion that **3a–e**^**TMS**^ undergo rapid interconversion with traces of [**3a**–**e**]^−^. Kinetic modeling, see Section 2.9, shows that **3a–e**^**TMS**^ do not need to be better fluoride (*k*_SF_) acceptors than TMSCF_3_ (*k*_F_) for there to be substantial acceleration in TMSF generation in stage III, provided that **3a–e**^**TMS**^ do not exergonically complex [CF_3_]^−^ to generate a siliconate (Scheme 5).^2c,2d,3^

### 2.5 Stage IV: TMSCF_3_ Depletion

After the vigorous TMSF evolution of stage III, the reaction enters a variable and sometimes prolonged period of near-stasis. During this, there is a slow but progressive reduction in the concentration of any remaining TMSCF_3_ by several processes, including formal exchange with Ph_3_SiF (a coproduct from anionic initiation by TBAT, Scheme 1) to generate Ph_3_SiCF_3_ and TMSF, see Section S1.3 in the Supporting Information. The behavior suggests that TMSCF_3_ is a powerful inhibitor of the chain reaction that releases the coumaranone **4**, vide infra, and must fall below a critical concentration for the transition from stage IV to stage V to occur. A variety of tests were conducted to support this conclusion; see Sections S7.1–3 in the Supporting Information. For example, briefly bubbling CO_2_ gas through the NMR sample at stage IV rapidly converts residual TMSCF_3_ into CF_3_CO_2_TMS/[CF_3_CO_2_][Bu_4_N]^+^ and stimulates the conversion of ketal **3**^**TMS**^ to coumaranone **4**. Removing the volatiles (CF_3_H, TMSF, THF, and TMSCF_3_) in vacuo and then redissolving the residue in THF also elicits a transition to stage V.^15^

### 2.6 Stage V: In Situ ^29^Si NMR Analysis of the Liberation of Coumaranone **4** from Ketal **3**^**TMS**^

**Figure 3:**
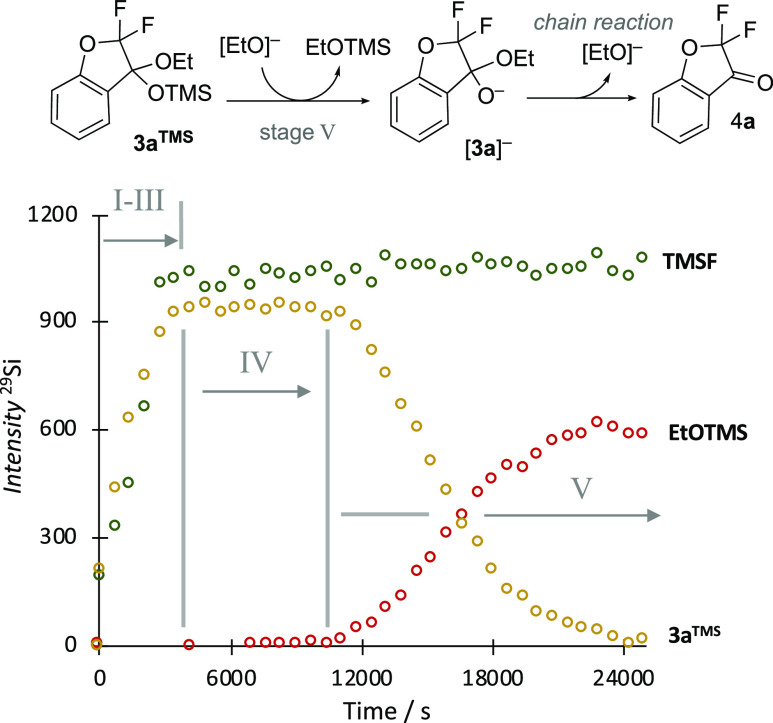
In situ ^29^Si NMR (INEPT) spectroscopic analysis of the conversion of salicylate **2a**^**H**^ into ketone **4a** at 285 K. The data indicates that desilylation of ketal **3a**^**TMS**^ in stage V proceeds via an anionic chain reaction with [EtO]^−^ as the carrier, with no further generation of TMSF.^8^ The ^29^Si signal intensities have not been corrected for the differing effects of magnetization transfer (INEPT) or relaxation at the three sites, and the analysis is only semiquantitative. Analogous results were obtained on analysis of the reaction of salicylate **2e**^**H**^ at ambient temperature; see Section S7.3 in the Supporting Information. The identity of the EtOTMS coproduct was confirmed by synthesis from EtOH and ^29^Si NMR (INEPT) analysis in THF.

For many of the salicylates studied, the duration of the stage IV induction period can be so extensive that stage V is not reached, even after prolonged in situ NMR reaction monitoring. To study stage V, we thus employed salicylates **2a**^**H**^ and **2e**^**H**^, which reliably liberate the corresponding coumaranones, **4a,e**, in a reasonable time scale without requiring triggering by additives or physicochemical manipulations, vide supra. To determine the fate of the TMS group derived from ketal **3a**^**TMS**^ in its conversion to ketone **4a**, we analyzed the full evolution of the reaction of **2a**^**H**^ by in situ ^29^Si NMR spectroscopy, Figure 3.

The insensitivity and slow relaxation of the ^29^Si nuclei required the process to be monitored at 285 K, with data acquired under a semiquantitative regime.^9^ Nonetheless, the temporal intensity profiles report on the mechanistic sequences of the process and correlate well with the general trends determined by quantitative in situ ^19^F NMR spectroscopy. The ^29^Si NMR spectroscopic analysis, Figure 3, shows that, in contrast to Scheme 2,^8^ the TMS is cleaved from ketal **3a**^**TMS**^ by ethoxide in stage V, with the chain reaction being initiated by elimination in anion [**3a**]^−^. If anion [**3a**]^−^, resulting from TMS cleavage in ketal **3a**^**TMS**^, has sufficient lifetime to eliminate the ethoxide anion, then this will propagate the chain reaction and generate ketone **4a**. Conversely, if [**3a**]^−^ is resilylated (Scheme 6), the chain is terminated and the process remains at stage IV until the TMSCF_3_ has been consumed, physically removed, or quenched in workup.^15^

### 2.7 Influence of Electron-Withdrawing Aryl-Ring Substituents on Side Reactions

**Scheme 7:**
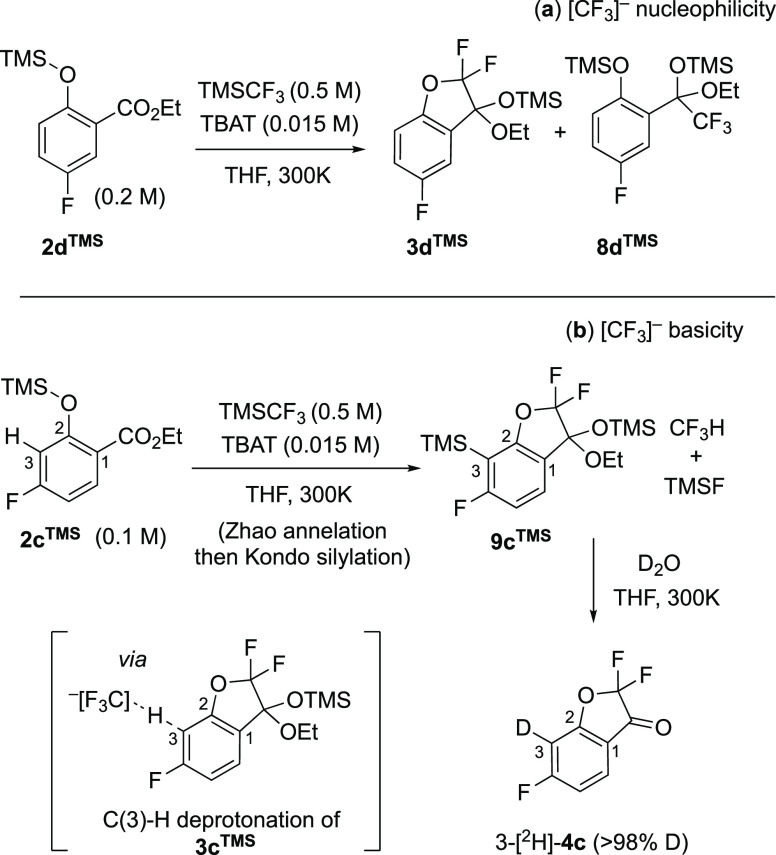
Side Reactions Arising from Nucleophilicity (a) and Basicity (b) of the Carbanion(oid) [CF_3_]^−^ Inset in (b) shows the generation of CF_3_H and an aryl anion(oid). The latter is rapidly silylated to give **9c**^**TMS**^. The deuterium incorporation 3-[^2^H]-**4c**/**4c** was estimated as 65/1 by ^19^F NMR spectroscopy, see Section S8.2 in the Supporting Information.

Salicylates **2c,d**^**H/TMS**^ undergo significant side reactions, as identified by in situ ^19^F NMR spectroscopy, and these are associated with the nucleophilicity and basicity of the carbanion(oid) [CF_3_]^−^,^2,3^Scheme 7. There is a nonlinear growth in the concentration of [CF_3_]^−^ through stages II and III, as modulated by the coupled equilibria, *K*_1_ and *K*_2_, see Section 2.8. The position of the fluorine on the aryl ring in the salicylate substrate impacts not only the inherent propensity of intermediates **2c,d**^**TMS**^ and **3c,d**^**TMS**^ to undergo side reactions with [CF_3_]^−^ but also the [CF_3_]^−^ concentration, via *K*_1_, vide infra.

The carbonyl ester group is susceptible to nucleophilic attack by [CF_3_]^−^,^16^ as confirmed by in situ ^19^F NMR spectroscopic analysis of the reaction of ethylbenzoate with TMSCF_3_ + TBAT to generate the *O*-silylated CF_3_-addition product. However, the *O*-TMS group in silyl ethers **2a–e**^**TMS**^ appears to exert considerable steric shielding and electronic deactivation of the ester unit, with little evidence for CF_3_-addition in **2b,c**,**e**^**TMS**^. An analogous deactivating effect is observed in methyl ether. For the case of silyl ether **2d**^**TMS**^, however, the meta position of the electron-withdrawing fluorine substituent relative to the ester is sufficiently activating to induce the generation of moderate quantities of what was assigned as the *O*-silylated CF_3_-addition product **8d**^**TMS**^, Scheme 7a, see Section S8.3 in the Supporting Information. Under the standard conditions, approximately 15% of **2d**^**TMS**^ is converted to **8d**^**TMS**^ during stage II.^17^

For salicylate **2c**^**TMS**^, the dominant side reaction is again related to the dynamic concentration of [CF_3_]^−^, but instead arises from aryl deprotonation-silylation of the ketal **3c**^**TMS**^ during stages II and III, see Section S8.1 in the Supporting Information. This general reaction class has been extensively developed by Kondo^4^ for a range of (hetero) arenes and requires suitably electron-withdrawing substituents to proceed on simple benzene ring systems. In the Zhao process, it becomes feasible in ketal **3c**^**TMS**^ because C(3)–H is located between two electron-withdrawing groups, C(4)–F and C(2)–OCF_2_, and is sufficiently C–H acidic to be deprotonated by [CF_3_]^−^, to generate CF_3_H and a transient aryl anion.^3c,3d^ The latter is rapidly silylated by TMSCF_3_, thus propagating the chain reaction,^3c^ and under the standard conditions, >50% conversion of **3c**^**TMS**^ into **9c**^**TMS**^ has occurred by the end of stage III. On applying the standard workup,^4,15^ the C(3)-TMS group in **9c**^**TMS**^ undergoes protonolysis, and ketone **4c** is obtained. Thus, any Kondo-silylation^4^ that occurs under Zhao’s conditions^5^ is ‘traceless’ in the absence of in situ analysis. By increasing the ratio [TMSCF_3_]_0_/[**2c**^**TMS**^]_0_ to extend the duration of stage III, the conversion of **3c**^**TMS**^ into Ar-silylated **9c**^**TMS**^ was increased to >98%, and on addition of D_2_O, coumaranone 3-[^2^H]-**4c** (>98% D) was generated, Scheme 7b, see Section S8.2 in the Supporting Information.

### 2.8 Anion Speciation and Rates of Conversion of Salicylates **2**^**TMS**^ to Ketals **3**^**TMS**^ in Stage II

**Scheme 8:**
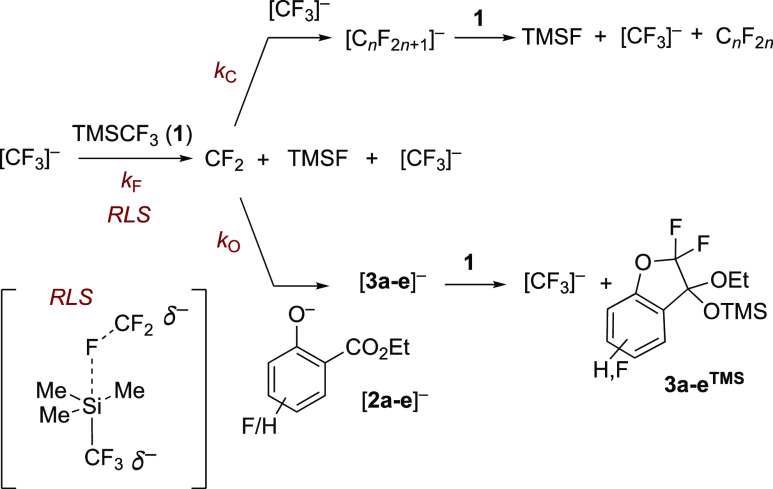
Rate-Limiting CF_2_ Generation via Concerted Fluoride Transfer (*k*_F_) from [CF_3_]^−^ to TMSCF_3_ (1)^ ^3b,3c^−3d^ and Competing Capture of CF_2_ by [CF_3_]^−^ (*k*_C_) and Salicylate (*k*_O_)

The rate of accumulation of the ketal, **3a–e**^**TMS**^, in stage II depends on three general processes: (i) the rate-limiting generation of CF_2_ by concerted F-anion transfer^3b^ (*k*_F_) from [CF_3_]^−^ and TMSCF_3_ (**1**), Scheme 8, (ii) the fractional efficiency (*f*) of CF_2_ capture by salicylate anion [**2a–e**]^−^ (*k*_O_) versus oligomerization (*k*_C_), and (iii) the net effect of any side-reactions (Σ_SR_, Section 2.7) that deplete **2a–e**^**TMS**^ and/or **3a–e**^**TMS**^.

**Figure 4:**
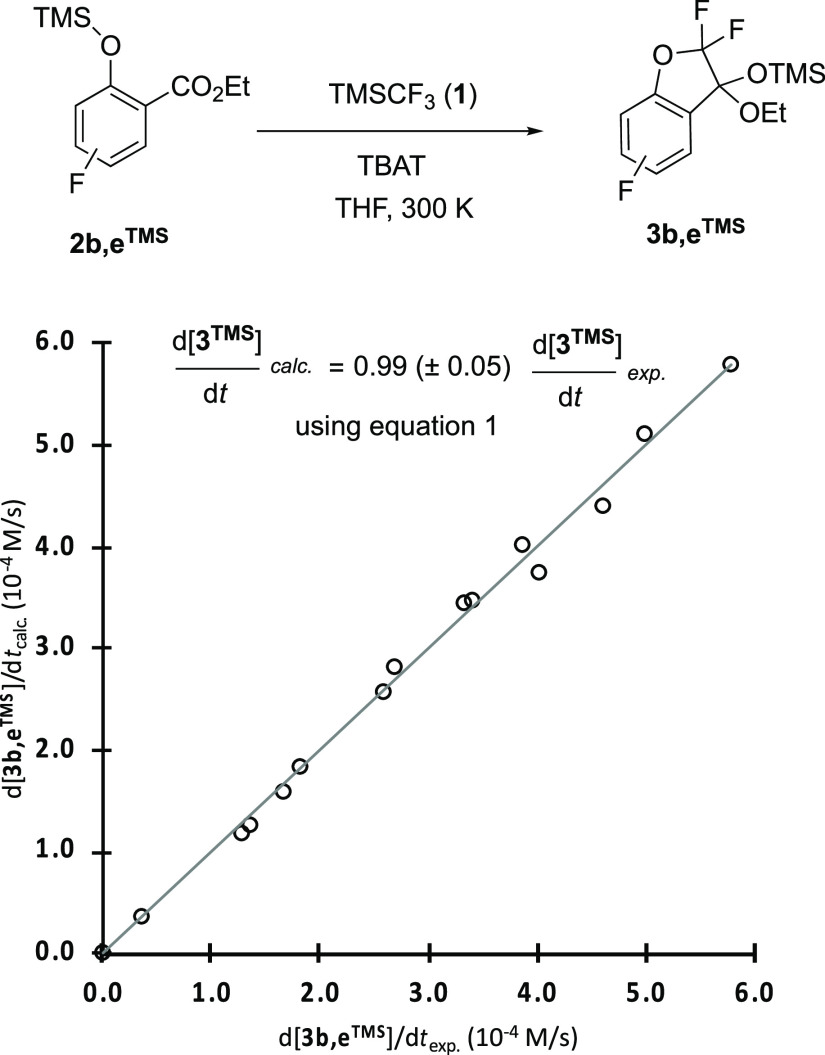
Correlation of the initial rates of generation of ketals **3b^TMS^** and **3e**^**TMS**^ in stage II predicted by the steady state rate approximation based on Schemes 8 and 9 and eq 1, with the experimentally determined values. TBAT = [Ph_3_SiF_2_]^−^[Bu_4_N]^+^. Experimental data from in situ ^19^F NMR spectroscopic analysis at 300 K, see Section S11.4 in the Supporting Information for full details of initial conditions. Fitting parameters *k*_F_/*K*_2_ = 4.2 ± 0.2 × 10^–2^ s^–1^, *K*_1_*K*_2_ = 1.5 ± 0.2 M^–1^ (**2b**^**TMS**^), and *K*_1_*K*_2_ = 0.23 ± 0.02 M^–1^ (**2e**^**TMS**^), estimated by standard linear regression.

**Scheme 9:**
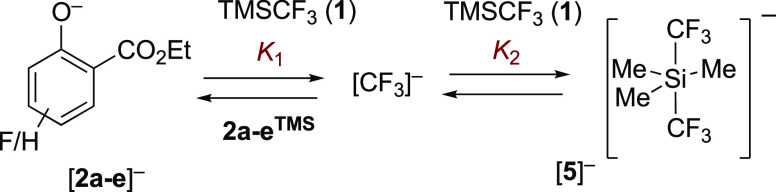
Coupled Anion Equilibria (*K*_1_ and *K*_2_) and the Influence of Phenolate Stability (1/*K*_1_), and the Concentrations of 2^TMS^ and TMSCF_3_ (1) on the Dynamic Concentration of [CF_3_]^−^

A series of approximations can be applied to derive the relationship between the concentrations of the reaction components, i.e., the three major anions ([**2**]^−^ + [CF_3_]^−^ + [**5**]^−^) ≈ [TBAT]_0_, the reagent [**1**]_*t*_ (TMSCF_3_), and the substrate [**2**^**TMS**^]_*t*_, with the dynamic concentration of [CF_3_]^−^, as determined by the coupled equilibria, *K*_1_ and *K*_2_, Scheme 9. Because siliconate generation (*K*_2_) is exergonic, the pre-equilibrium and steady state approximations can be combined and simplified to eq 1, when Σ_SR_ ≈ 0 and *f* ≈ 1; see Section S11.3 in the Supporting Information for a full derivation
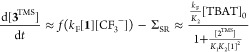
1Equation 1 indicates that lower phenolate stability (larger *K*_1_), higher reagent concentration ([**1**]^2^), and lower silyl ether concentration ([**2**^TMS^]) all increase the rate of CF_2_ generation in stage II toward a rate maximum of (*k*_F_[TBAT]_0_/*K*_2_), i.e., kinetic saturation. Two of the salicylates (**2b,e**^**TMS**^) cleanly convert to the ketals **3b,e**^**TMS**^ in near-parallel with TMSF generation, indicative that Σ_SR_ ≈ 0 and *f* ≈ 1.^18^ These features allow eq 1 to be tested experimentally, as shown in Figure 4, across a variety of initial conditions.

Inclusion of an additional term in eq 1 for CF_2_ generation by transfer of fluoride from [CF_3_]^−^ to **2b,e**^**TMS**^ (analogous to *k*_SF_ for **3**^**TMS**^ in Scheme 6) attenuated the correlation in Figure 4, indicative that TMSCF_3_ is the dominant (>95%) acceptor (*k*_F_, Scheme 8) in stage II. The kinetic approximation shown in eq 1 was then further explored in the analysis of the relative rates of conversion of pairs of salicylates (i and ii) into their ketals (**3**^**TMS**^) in stage II, eqs 2 and 3; see Section S11.3 in the Supporting Information for full derivation
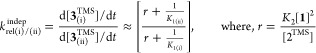
2
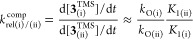
3

**Chart 2:**
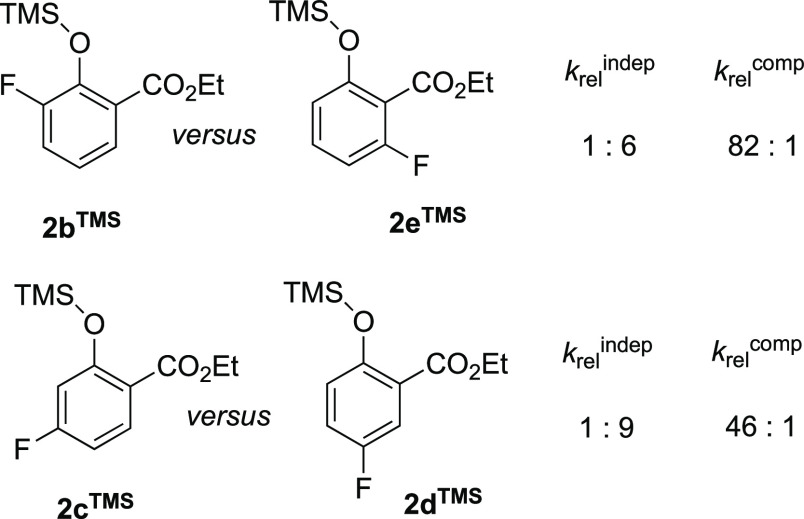
Examples of Relative Rates (*k*_rel_) of Conversion of 2^TMS^ to 3^TMS^, Evaluated Independently and in Competitiona

The relative rates (*k*_rel_ (i), (ii)) are compared under the two regimes. In the first, the initial rates of independent reactions were estimated under otherwise identical conditions, eq 2, and the substrate with the electron-withdrawing F-substituent closer to the phenolic position (lower *K*_1_, eq 2) was found to react slower, **2b**^**TMS**^ and **2c**^**TMS**^, Chart 2. Conversely, when the relative rates of reactions were estimated in competition reactions, eq 3, the substrates with the electron-withdrawing F-substituent closer to the phenolic position (lower *K*_1_, eq 3) were found to react faster, **2b**^**TMS**^ and **2c**^**TMS**^, Chart 2.

a For the factors controlling *k*_rel_, see eqs 2 and 3.

The relative rates, Chart 2, are opposite in independent versus competition reactions, as modulated by the *K*_1_ values of the substrates, eqs 2 and 3, but are qualitatively inverted. This arises from the additional effects of the concentrations ([**1**], [**2**^**TMS**^]) and thus the values for *r* on the relative absolute rates, eq 2, and the effects of relative efficiencies of carbene trapping (*k*_O_) by the salicylate anions, [**2**]^−^, eq 3, on the competitive rates. The overall kinetic behavior of stage II, eqs 1–3 is thus consistent with the rate-limiting generation (*k*_F_) of the singlet carbene CF_2_, and its rapid trapping (*k*_O_) by salicylate anions, [**2**]^−^, as modulated by the coupled equilibria *K*_1_ and *K*_2_ (Schemes 8 and 9).

### 2.9 Overarching Reaction Network for Stages I–V in the Anion-Initiated Conversion of Salicylates (**2**^**H**^) to Coumaranones (**4**) by TMSCF_3_

**Figure 5:**
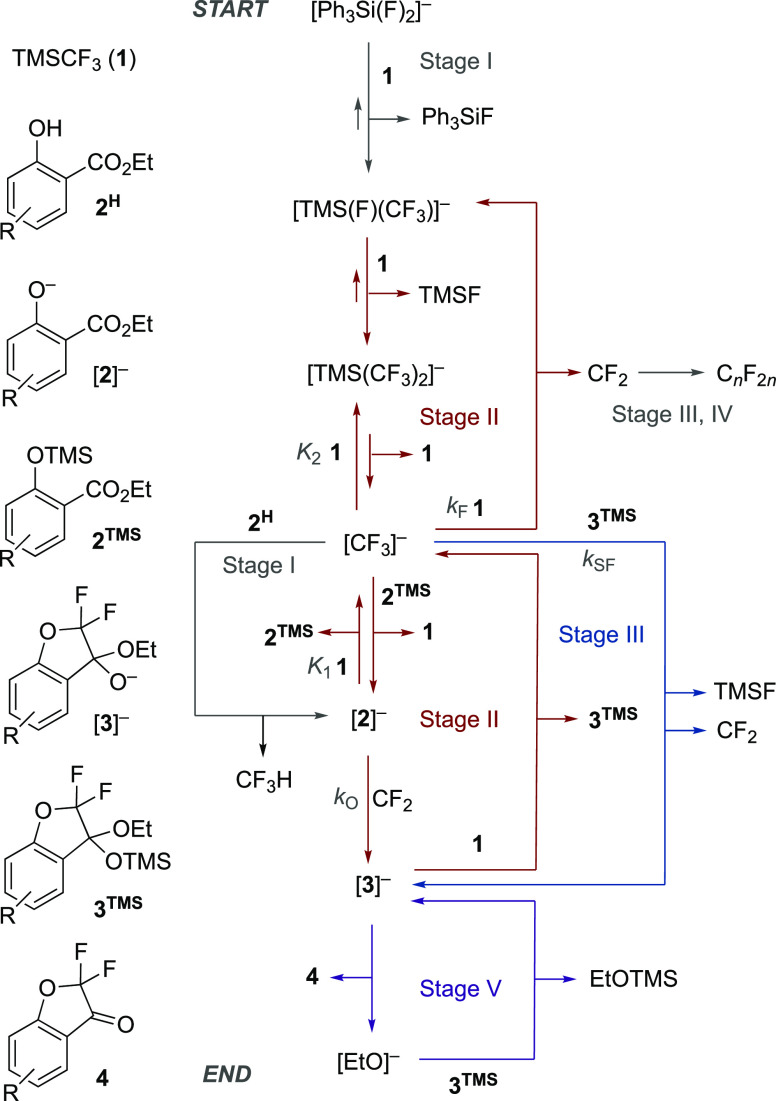
Minimal reaction network connecting the seven anions involved in stages I, II, III, IV, and V for the TBAT (Ph_3_SiF_2_]^−^[Bu_4_N]^+^) initiated the conversion of generic salicylate (**2**^H^) into α,α-difluoro-3-coumaranone (**4**) by TMSCF_3_ (1). In addition to the anion-mediated formal oligomerization of CF_2_, stage IV includes other processes not shown, e.g., conversion of Ph_3_SiF into Ph_3_SiCF_3_.

Having elucidated the dominant anion-speciations, and the interconnecting equilibria and reactions that govern the net conversion of ethyl salicylates **2a–e**^**H**^ into the corresponding coumaranones (**4a**–**e**),^5^ an overarching, albeit simplified, reaction network can be proposed, for the reactions of ethyl salicylates **2**^**H**^ in general, Figure 5. After initiation by TBAT (stage I), there are two productive anion-chain processes that convert **2**^**H**^ → **4**. These proceed via two discrete kinetic regimes (**2**^**H**^ → [**2**]^−^ → [**3**]^−^ → **3**^**TMS**^, in stage II; then **3**^**TMS**^ → **4**, in stage V). These are separated by stage III, which consumes the majority of the remaining TMSCF_3_ (**1**), and stage IV, during which the TMSCF_3_ (**1**) concentration falls low enough to allow ethoxide elimination from anion [**3**]^−^ and the onset of the chain reaction that releases the coumaranone **4** in stage V. Progression through the five stages leads to the complex temporal evolution of the overall process, Figure 1.

### 2.10 Kinetic Simulations of Stages I–III

**Figure 6:**
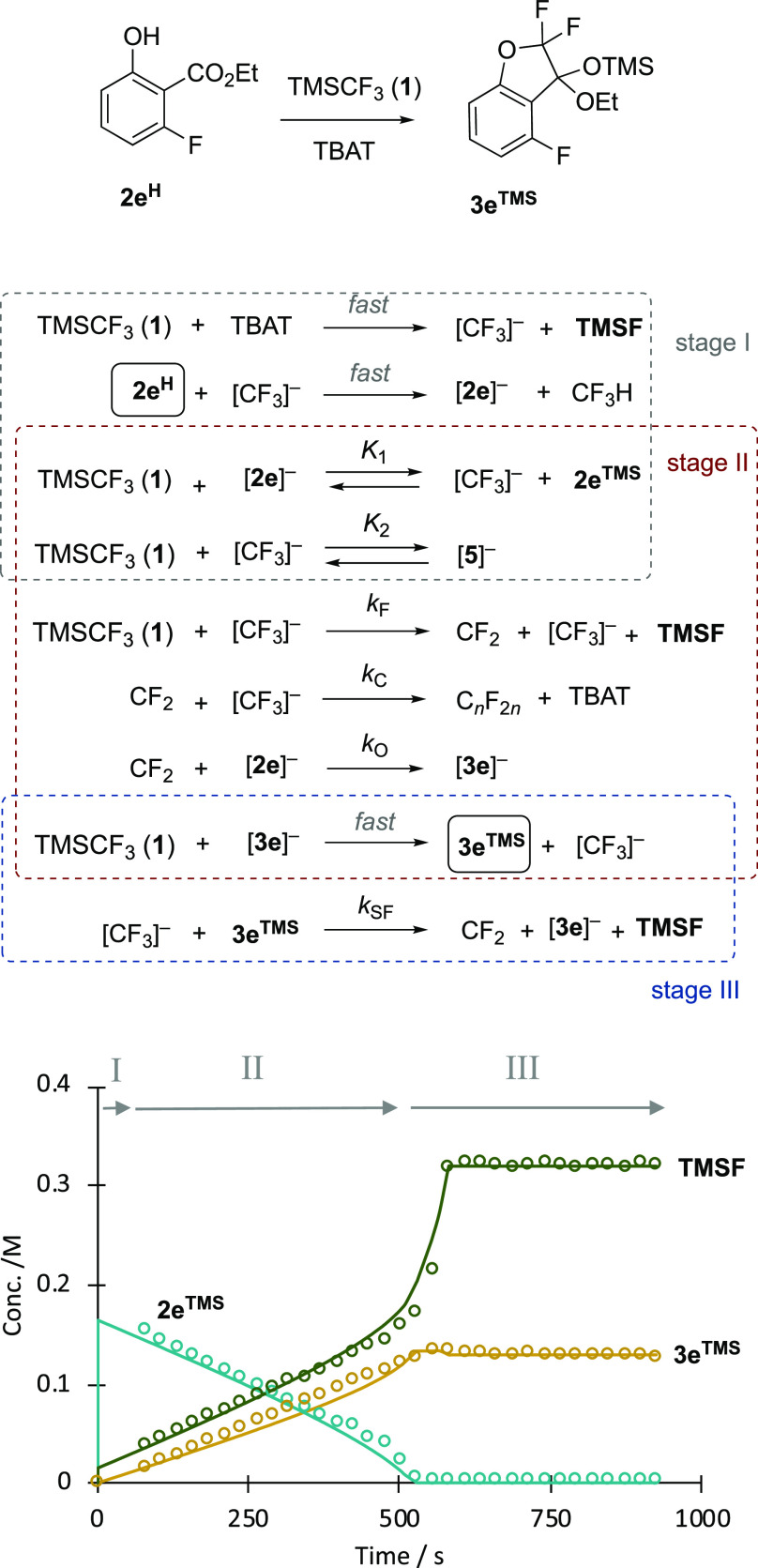
Numerical methods kinetic simulation^9^ of a simplified model for stages I–III in the conversion of salicylate **2e**^H^ into ketal **3e** with acceleration on transition from stage II to stage III as salicylate **2e**^TMS^ becomes depleted. Data from in situ ^19^F NMR (376 MHz) spectroscopic analysis. Conditions: **2e**^H^ 0.18 M, TMSCF_3_ (1) 0.49 M, TBAT, 0.015 M, 0.08 equiv, THF, N_2_, RT. TBAT = [Ph_3_SiF_2_]^−^[Bu_4_N]^+^. Stage I is complete within the “dead-time” between the addition of the TBAT and the measurement of the first NMR spectrum. Lines through the data points are the temporal concentrations predicted by the stages I–III minimal model shown. Stage IV is a period of variable duration quasi-stasis, and neither stage IV nor V are simulated. For details, further examples, and discussion of this and other models, see Section S11.1 in the Supporting Information.

Considerable effort was made to analyze and simulate the kinetics of the overall process.^9^ A key issue is the complexity of stage IV and its transition to sigmoidal growth of ketone **4** in stage V, neither of which was found to be experimentally reproducible. We thus focused on the generation of a model for kinetic simulation of stages I–III that responds with reasonable fidelity to experimental data obtained at various initial concentrations of TMSCF_3_ ([**1**]_0_), salicylate ([**2e**^**H**^]_0_), silyl ether ([**2e**^**TMS**^]_0_), and [TBAT]_0_. The final model and an example of its application to an experimental dataset are shown in Figure 6. For further examples and discussion of this and other models, see Section S11.1 in the Supporting Information. As the concentration of the salicylate **2**^**TMS**^ decays, the concentration of [CF_3_]^−^ rises (*K*_1_), leading to a surge in surrogate fluoride acceptance by **3**^**TMS**^, *k*_SF_, and the system transitions from stage II to stage III.

## 3 Conclusions

The mechanism of the Zhao method for the conversion of ethyl salicylates **2**^**H**^ into α,α-difluoro-3-coumaranones, **4**,^5^ has been investigated by in situ ^19^F/^29^Si NMR spectroscopy and analysis of the absolute and relative kinetics as a function of concentrations and identities of reactants and reagents. The process is found to evolve in five stages, as shown in Figure 1. Each stage has a discrete speciation of anions (Figure 5) that modulates the required sequence of silyl-transfer chain reactions.^8^ The distinction of the five stages allows four important practical conclusions to be drawn about the inherent behavior of different salicylate esters, **2**^**H**^, and how conditions can be selected to favor their effective and efficient conversion to the corresponding α,α-difluoro-3-coumaranones, **4**.

(1) Stage I necessarily consumes 1 equiv of TMSCF_3_ (**1**) and can be bypassed by directly employing silyl ether, **2**^**TMS**^, which is readily prepared from salicylate **2**^**H**^ using TMSCl, see Section S12 in the Supporting Information. Starting at stage II allows the reaction to be safely run at much higher concentrations without risk of overpressure arising from the evolution of 1 equiv of the greenhouse gas CF_3_H.
(2) The two key productive steps are the rate-limiting generation of the singlet carbene CF_2_ in stage II and the desilylation of ketal **3e**^**TMS**^ by [EtO]^−^ in stage V, and these have opposing requirements in terms of concentration of TMSCF_3_ (**1**). The kinetic dependencies in stage II, eq 1, mean that the generation of ketal **3**^**TMS**^ is disproportionately accelerated by high reagent (**1**) and low substrate (**2**^**TMS**^) concentrations. Reactions run at scale may thus benefit from the slow addition of **2**^**TMS**^ to TMSCF_3_ (**1**) in stage II, at a rate that holds concentrations of the two components in ratios for which the system is most productive.
(3) Electron withdrawing groups, e.g., fluorine, on the aryl ring of **2**^**TMS**^ not only inherently inhibit stage II (*K*_1_, Scheme 3) but also accelerate side reactions such as nucleophilic attack at the ester in **2**^**TMS**^ and Kondo silylation^4^ of the aromatic ring in **3**^**TMS**^ (Scheme 7). These substrates benefit from being run with a significantly raised TMSCF_3_ (**1**) concentration, not only to accelerate the conversion of **2**^**TMS**^ to **3**^**TMS**^ but also to suppress (*K*_2_, Scheme 5)^3^ the concentration of [CF_3_]^−^ and thus attenuate undesired side reactions.
(4) TMSCF_3_ (**1**) is rapidly but unproductively consumed in stage III by conversion to C_*n*_F_*2n*_ + TMSF,^14^ a process that is accelerated by ketal **3a**^**TMS**^. The presence of even low concentrations of TMSCF_3_ (**1**) powerfully inhibits stage V, leading in some cases, to the system remaining for a prolonged period at stage IV. Aqueous quenching of the reaction at the onset of stage III bypasses stages IV and V, leading to rapid hydrolysis of ketal **3a**^**TMS**^. Alternatively, excess TMSCF_3_ (**1**) can be removed in vacuo or by distillation, and residual anion is then allowed to mediate the chain-reaction that converts ketal **3a**^**TMS**^ into ketone **4** + EtOTMS under anhydrous conditions.^15^

## 4 Safety Considerations

Anionic initiation of the Zhao process^5^ for the conversion of ethyl salicylates **2**^**H**^ into α,α-difluoro-3-coumaranones, **4**, generates 1 equiv of CF_3_H, a greenhouse gas (bp −82 °C). While CF_3_H is soluble in THF, in our experience, it begins to evolve from solution at concentrations above 0.3 M. The use of sealed reaction vessels or employing salicylate **2**^**H**^ at concentrations in excess of 0.3 M can therefore lead to hazardous overpressures or spontaneous gaseous eruptions on opening the vessel. The initiation step is also considerably exothermic, and the use of the readily prepared silyl ether, **2**^**TMS**^, instead of the phenolic form **2**^**H**^ is advisible. The anionic chain processes inherently generate superstoichiometric TMSF (bp 19 °C), and with some substrates, the rate of TMSF generation in stage III accelerates considerably, potentially leading to uncontrolled exothermic events. Reactions involving anionic initiation of TMSCF_3_ (**1**) invariably cogenerate a complex range of perfluoroalkenes, C_*n*_F_2*n*_, some of which are volatile and toxic.^19^ For all of the above reasons, caution should be exercised in the use of these processes, especially on scale-up.

## Data Availability

The data underlying this study are available in the published article and its Supporting Information.

## Supporting Information Available

Procedures of reactions, characterization, in situ NMR data, kinetic analyses, and simulations (PDF)

## References

[1] aBeier P.; Zibinsky M.; Prakash G. K. S. Nucleophilic Additions of Perfluoroalkyl Groups. Org. React. 2016, 91, 1–492. 10.1002/0471264180.or091.01.

[2] aRuppert I.; Schlich K.; Volbach W. Die Ersten CF_3_-Substituierten Organyl(Chlor)Silane. Tetrahedron Lett. 1984, 25, 2195–2198. 10.1016/S0040-4039(01)80208-2.

[3] aJohnston C. P.; West T. H.; Dooley R. E.; Reid M.; Jones A. B.; King E. J.; Leach A. G.; Lloyd-Jones G. C. Anion-Initiated Trifluoromethylation by TMSCF_3_: Deconvolution of the Siliconate-Carbanion Dichotomy by Stopped-Flow NMR/IR. J. Am. Chem. Soc. 2018, 140, 11112–11124. 10.1021/jacs.8b06777. 30080973 PMC6133236

[4] aNozawa-Kumada K.; Inagi M.; Kondo Y. Highly Chemoselective DMPU-Mediated Trialkylsilylation of Terminal Alkynes Using Trifluoromethyltrialkylsilane. Asian J. Org. Chem. 2017, 6, 63–66. 10.1002/ajoc.201600472.

[5] Cai Y.; Zhu W.; Zhao S.; Dong C.; Xu Z.; Zhao Y. Difluorocarbene-Mediated Cascade Cyclization: The Multifunctional Role of Ruppert–Prakash Reagent. Org. Lett. 2021, 23, 3546–3551. 10.1021/acs.orglett.1c00962. 33913711

[6] aWang F.; Fu R.; Chen J.; Rong J.; Wang E.; Zhang J.; Zhang Z.; Jiang Y. Metal-free synthesis of gem-difluorinated heterocycles from enaminones and difluorocarbene precursors. Chem. Commun. 2022, 58, 3477–3480. 10.1039/d2cc00383j. 35191446

[7] aLee Y. H.; Shin M. C.; Yun Y. D.; Shin S. Y.; Kim J. M.; Seo J. M.; Kim N. J.; Ryu J. H.; Lee Y. S. Synthesis of aminoalkyl-substituted aurone derivatives as acetylcholinesterase inhibitors. Bioorg. Med. Chem. 2015, 23, 231–240. 10.1016/j.bmc.2014.11.004. 25468034

[8] Several of the species and steps in the mechanism proposed by Zhao,^5^ including phenolate generation, CF_2_-trapping, and accumulation of **3**^TMS^ before conversion to **4**, are supported by the data reported herein. However, the overall sequence for these is inconsistent with both the stoichiometry and the temporal evolution of the coproducts reported in preliminary in situ ^19^F NMR studies of the reaction of isobutyl salicylate at an unreported temperature.^5^ For example, **3a**^TMS^ would need to be liberated in concert with CF_3_H, and **3a**^TMS^ converted to **4a** by cogenerating TMSF. Moreover, anion inventory, i.e., where Σ_anions_ = [TBAT]_0_, of the proposed sequence,^5^ shows that stoichiometric TBAT would be required to fully convert **2a**^H^ into [**3a**]^−^, whereas high yields are obtained in reactions employing substoichiometric TBAT. The sequence^5^ also includes the direct generation of CF_2_ by α-elimination in [CF_3_]^−^, which has been shown to be endergonic and kinetically noncontributive when the counteranion is [Bu_4_N]^+^, see ref (3b).

[9] Ben-Tal Y.; Boaler P. J.; Dale H. J. A.; Dooley R. E.; Fohn N. A.; Gao Y.; García-Domínguez A.; Grant K. M.; Hall A. M. R.; Hayes H. L.; Kucharski M. M.; Wei R.; Lloyd-Jones G. C. Mechanistic Analysis by NMR Spectroscopy: a Users Guide. Prog. Nucl. Magn. Reson. Spectrosc. 2022, 129, 28–106. 10.1016/j.pnmrs.2022.01.001. 35292133

[10] Attempts to corroborate the assignment of the chemical shift of the salicylate {[**2d**]^−^[Bu_4_N]^+^} by ^19^F NMR analysis of the titration of salicylate **2d**^H^ with [Bu_4_N]^+^[OH]^−^ led to competing ester hydrolysis. Analogous titrations of the phenol **2d**^H^ were complicated by generation of the homoconjugate anion ([ArO··H··OAr]^−^) and competing ester hydrolysis.

[11] Anionic ring-opening C–F fluoride displacements are implicated in the generation of rearranged products after addition of CF_2_ to TMS-enol ethers, see ref (3a).

[12] Wang F.; Luo T.; Hu J.; Wang Y.; Krishnan H. S.; Jog P. V.; Ganesh S. K.; Prakash G. K. S.; Olah G. A. Synthesis of gem- Difluorinated Cyclopropanes and Cyclopropenes; Trifluoromethyl- trimethylsilane as a Difluorocarbene Source. Angew. Chem., Int. Ed. 2011, 50, 7153–7157. 10.1002/anie.201101691. 21681877

[13] Analogous pathways have been elucidated by Grygorenko and co-workers for addition of CF_2_ to imidazoles, see ref (6c).

[14] As is evident from the relative temporal concentrations of **7** and TMSF in Figure 2, the concentration of “available” CF_2_ in stage III is low. We have previously shown, see refs (3b)–^3d^, that high concentrations of [Bu_4_N]^+^[CF_3_]^−^ rapidly oligomerize CF_2_; and that this is usually accompanied by transient C_2_F_4_ (TFE) accumulation. However, the latter is not detected in stage III of the process investigated herein, suggesting that C_*n*_F_2*n*_ may be generated through other oligomerization processes, for example sequential capture by [**2**]^−^ or [**3**]^−^ then fluoride elimination.

[15] It is noteworthy that the experimental procedure reported by Zhao, see ref (5), includes analysis of the reaction by TLC, and then concentration in vacuo prior to isolation of the coumaranones, **4**, by column chromatography. We have found that both steps, i.e., TLC and concentration in vacuo, can reduce [TMSCF_3_] below that required to trigger transition to stage V. Addition of water initiates stage V by conversion of TMSCF_3_ to TMSOH (^29^Si NMR) and CF_3_H (^19^F NMR), see Section S7.3 in the Supporting Information.

[16] aSingh R. P.; Cao G.; Kirchmeier R. L.; Shreeve J. M. Cesium Fluoride Catalyzed Trifluoromethylation of Esters, Aldehydes, and Ketones with (Trifluoromethyl)trimethylsilane. Org. Chem. 1999, 64, 2873–2876. 10.1021/jo982494c. 11674359

[17] For the isomeric silyl ether **2b**^TMS^ the influence of the *meta*-F is considerably suppressed, with only traces of **8b**^TMS^ detected. This may be due to a change in the dominant Ar-O-Si conformation(s) resulting in augmented steric shielding of the ester by the TMS group.

[18] Analysis of [TMSF]_*t*_ versus [**3e,b**^TMS^]_*t*_ indicates that *f* ≥ 0.95 throughout stage II. The subunity correlation suggests that there are additional minor pathways that lead to TMSF that do not involve free CF_2_ generation; see also ref (14).

[19] Patocka J. Perfluoroisobutene: Poisonous Choking Gas. Vojen. Zdrav. Listy 2019, 88, 98–105. 10.31482/mmsl.2019.006.

