# Supplementary material for: TMSCF3-Mediated Conversion of Salicylates into α,α-Difluoro-3-coumaranones: Chain Kinetics, Anion-Speciation, and Mechanism
Source: J Org Chem. 2023 Dec 2;88(24):17450–60. doi: 10.1021/acs.joc.3c02219 (PMC10729029; doi:10.1021/acs.joc.3c02219)
Supplement: Supplementary file 1 — jo3c02219_si_001.pdf [file jo3c02219_si_001.pdf]

SUPPORTING INFORMATION

$TMSCF_3$-Mediated Conversion of Salicylates into α,α-Difluoro-3-Coumaranones: Chain Kinetics, Anion-Speciation, and Mechanism

Hannah B. Minshull and Guy C. Lloyd-Jones.*

School of Chemistry, University of Edinburgh, Joseph Black Building, Edinburgh, EH9 3FJ

*email: guy.lloyd-jones@ed.ac.uk

Table of Contents

S1. General considerations **S5**

S1.1 Reaction monitoring S5

S1.2 General procedure employed for *in-situ* monitoring by ^{19}F NMR spectroscopy S5

S1.3 Reaction Monitoring Spectra S6

S1.3.1 Ethyl 2-hydroxybenzoate S6

S1.3.1 Ethyl 3-fluoro-2-hydroxybenzoate ($\mathbf{2b^H}$) S9

S1.3.1 Ethyl 4-fluoro-2-hydroxybenzoate ($\mathbf{2c^H}$) S10

S1.3.1 Ethyl 5-fluoro-2-hydroxybenzoate ($\mathbf{2d^H}$) S12

S1.3.1 Ethyl 6-fluoro-2-hydroxybenzoate ($\mathbf{2e^H}$) S14

S1.4 Evidence for generation of siliconate [$\mathbf{5}^-$] S16

S2. Ketal formation: Variation of initial concentrations **S17**

S2.1 Initiator variation (6-fluoro, $\mathbf{2e^H}$) S17

S2.2 TMSCF3 variation (6-fluoro, $\mathbf{2e^H}$) S17

S2.3 TMSCF3 variation (3-fluoro, $\mathbf{2b^H}$) S18

S2.4 TMSCF3 variation (4-fluoro, $\mathbf{2c^H}$) S18

S2.5 Phenol variation (6-fluoro, $\mathbf{2e^H}$) S19

S3. Evidence for Generation of Salicylate TMS ethers ($\mathbf{2^{TMS}}$) – Stage I **S20**

S3.1 **Evidence for Salicylate TMS ethers ($\mathbf{2^{TMS}}$) in Equilibrium with Salicylate Anions [$\mathbf{2}^-$].** S20

S3.2 Use of TMS ether ($\mathbf{2a,d,e^{TMS}}$) as the substrate S21

S3.3 $TMSCF_3$ variation (TMS ether, $\mathbf{2a^{TMS}}$) S22

S3.4 Titration data S23

S4. Evidence for CF_2 Generation – Stage II **S24**

S4.1 Carbene trapping by alkene **6** S24

S4.2 Reactions of Salicylates $\mathbf{2^H}$ with $TESCF_3$ S25

S4.3 Reaction of Salicylate TMS Ether with $TESCF_3$ S26

S5. Accelerating rate of TMSF generation – Stage III **S27**

S5.1 Transient Dynamic line broadening S27

S5.2 Comparison of rate of accelerated TMSF generation in stage III,
across substrates **2a-e.** S28

S6. Evidence for CF_3 addition in stage II **S29**

S6.1 Reactions of reference esters S29

S7. Difluoro coumaranone (4) formation – Stage V **S31**

S7.1 CO_2 addition S31

S7.2 KOEt addition S32

S7.3 H_2O addition S33

S7.4 ^{29}Si monitoring S34

S8. Additional Observations **S35**

S8.1 Kondo Silylation S35

S8.2 D2O quenching of Kondo Silylation product **9c** S35

S8.3 Experiments comparing ethyl and methyl salicylates S38

S9. Relative Rates of Salicylates in Competition **S39**

S9.1 **.** Competition reaction, ethyl salicylate (**$2a^H$**) and
ethyl 5-fluoro-2-hydroxybenzoate (**$2d^H$**) S39

S9.2 Competition between ethyl salicylate (**$2c^H$**) and
ethyl 5-fluoro-2-hydroxybenzoate (**$2d^H$**) S40

S9. Competition between salicylate silyl ethers, **$2a^{TMS}$** and **$2d^{TMS}$.** S41

S9. Competition between salicylates, **$2b^H$** and **$2e^H$.** S42

S9.5 Addition of $TMSCF_3$ During Stage III S43

S9.6 Addition of $TESCF_3$ During Stage II/III of Reaction Initiated with$TMSCF_3$ S43

S10. Kinetic Simulations of the Reported Mechanism **S45**

S11. Kinetic Analyses of the Expanded Mechanism **S50**

S11.1. Kinetic Simulations S50

S11.2. Additional Notes S50

S11.3. Derivation of Steady State, Rapid Pre-Equilibrium Approximations S53

S11.4 Analysis of Initial Rate of Generation of **3b,e**^{TMS} and Correlation with Steady State Approximation; Figure 4 in main manuscript. S55

S12. Experimental S56

S13. References S62

S14. ^{1}H, ^{19}F, ^{13}C, ^{29}Si NMR spectra and IR spectra S63

S1. General Considerations

Reagents were purchased from Fluorochem and Merck. Ethyl salicylate and TBAT were introduced into the glovebox without additional purification. Trifluoromethyltrimethylsilane, $TMSCF_3$, and trifluoromethyltriethylsilane $TESCF_3$ (purchased from Fluorochem) were distilled and degassed before being introduced into the glovebox. Fluorobenzene was distilled from calcium hydride, and stored over 4 Å molecular sieves in the glovebox. All glassware, NMR tubes and syringes were oven-dried for at least 24 h prior to use, or introduction to the glovebox. Anhydrous tetrahydrofuran (THF) (unstabilised HPLC grade) was obtained by passage through a column of anhydrous alumina using an MBraun SPS-800 system directly into an MBraun glovebox, and used directly.

Instruments: ^{19}F NMR reaction monitoring on a Bruker Avance III 400 MHz spectrometer with a Prodigy cryoprobe, at a temperature of 300 K. Spectra were acquired using 'multi_zgvd' command using a 10° pulse angle with an acquisition time of 3 s, relaxation delay of 5 s, and fixed delay of 15-25 s between spectra. All ^{19}F spectra were obtained with a single scan. Spectra were processed using MestReNova software version 14. Fluorobenzene was used as an internal standard for substrates **2b-e^H** (δ_F = −114.0 ppm) and 1-fluoronaphthalene (δ_F = −125.0 ppm) for substrate **2a^H**. The internal standard was used to calculate the concentration of each species at each time point.

S1.1. Reaction monitoring

Unless stated otherwise, all reactions were set up in the glovebox in NMR tubes (5 mm diameter) with a Precision Seal® rubber septa caps (Z554014) which were purchased from Sigma-Aldrich. All phenols (**2a-e^H**) and $TMSCF_3$ stock solutions were prepared by directly weighing the reagents into volumetric flasks. These stock solutions were stored in the glovebox freezer (-40 °C). Stock solutions of TBAT were prepared freshly on the day that reaction monitoring was carried out. Gas-tight microsyringes were used to measure the volume of stock solution required for each specific reaction.

S1.2. General procedure employed for *in-situ* monitoring by ^{19}F NMR spectroscopy:

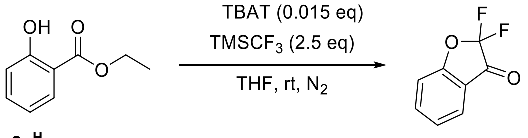

2a^H **4a**

In a nitrogen filled glovebox, 200 uL from a stock solution of ethyl 2-hydroxybenzoate (**2a^H**, 1 M) and fluoronaphthalene (1 M) as an internal standard in THF were added to a vacuum-oven-dried NMR tube. 200 uL of $TMSCF_3$ (2.5 M) in THF was added, followed by THF (500 uL). The NMR tube was capped with a rubber septum (Sigma Aldrich) and shaken thoroughly, horizontally along the longest axis. Each stock solution used its own syringe to prevent contamination. The NMR tube was then removed from the glovebox, and placed into the NMR spectrometer at 300 K. The spectrometer was shimmed (^1H) and tuned (^{19}F) without a deuterium lock. The sample was removed from the spectrometer and 100 uL from a TBAT stock solution (0.15 M) was added through the rubber septum with a syringe. The NMR tube was shaken thoroughly horizontally for 30 s and placed back into the spectrometer, recording the time between the addition of TBAT and the first spectrum acquisition with a stopwatch. The total volume for each reaction was 1 mL.

S1.3. Reaction Monitoring spectra

Each reaction presented in this section was monitored by ^{19}F NMR (376 Hz) at 300 K. All kinetic plots have a fixed delay of 15 s between scans. The formation and decay of all intermediates and major side products are shown in the kinetic profile given below for the reaction of **2a-e^{H}** (0.2 M), $TMSCF_3$ (0.5 M), TBAT (0.015 M). Significant minor side products are also detected, which includes a number of CF_3-addition species, and products thereof.

S1.3.1 Ethyl 2-hydroxybenzoate

Presented in Figure S1 are three ^{19}F NMR spectra; (a) before initiation by TBAT, (b) during the reaction generating ketal intermediate **3a^{TMS}** and (c) after near-complete conversion to ketone **4**, for the reaction of ethyl 2-hydroxybenzoate (**2a^{H}**, 0.2 M) and $TMSCF_3$ (0.5 M) with TBAT (0.015 M) as initiator and fluoronaphthalene (0.2 M) as internal standard (IS). The reaction was conducted at 300 K. Also shown are two sets of 'stacked' spectra from the reaction monitoring, at two levels of vertical magnification.

a)

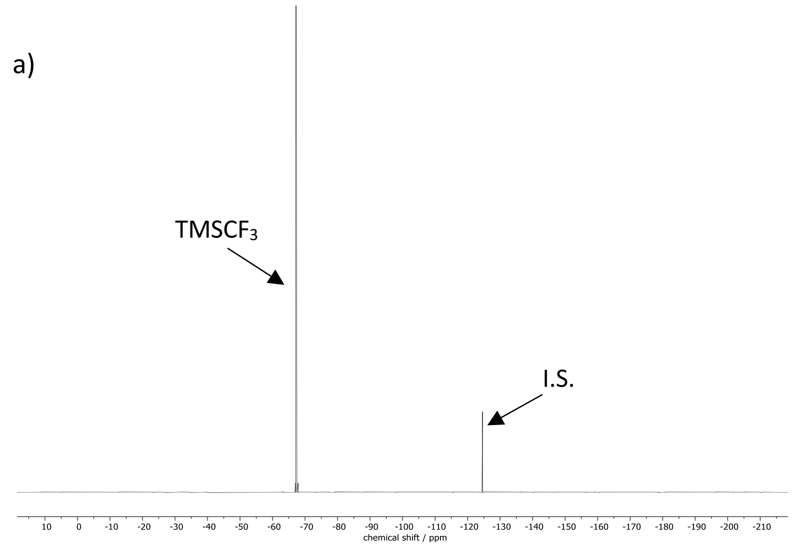

b)

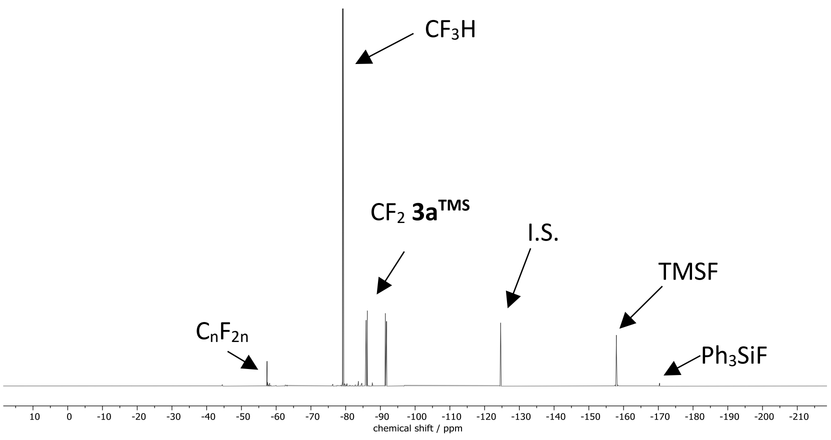

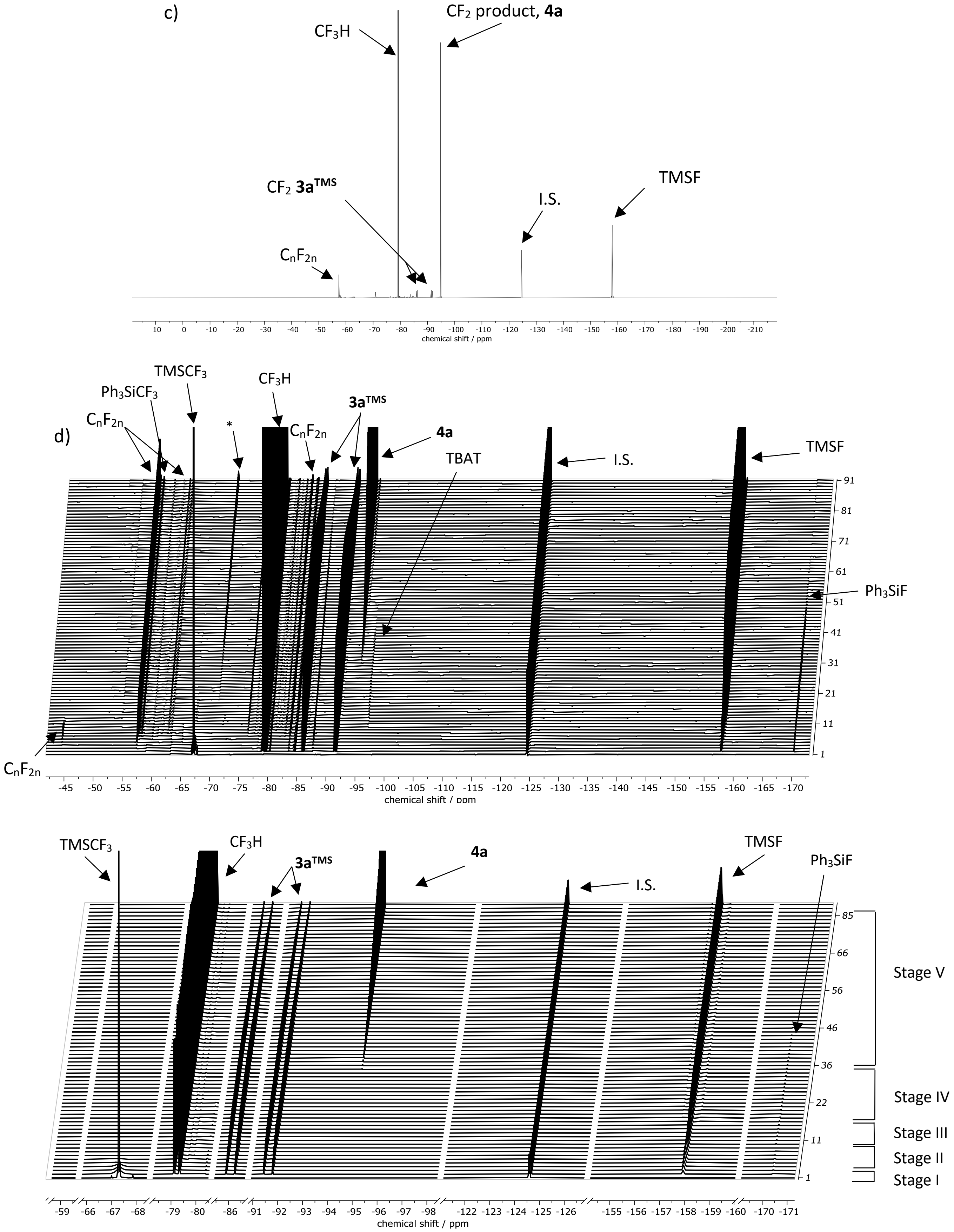

c)
CF$_3$H
CF$_2$ product, **4a**
CF$_2$ **3a^{TMS}**
C$_n$F$_{2n}$
I.S.
TMSF
chemical shift / ppm
d)
TMSCF$_3$
Ph$_3$SiCF$_3$
C$_n$F$_{2n}$
*
CF$_3$H
3a^{TMS}
C$_n$F$_{2n}$
4a
TBAT
I.S.
TMSF
Ph$_3$SiF
C$_n$F$_{2n}$
chemical shift / ppm
TMSCF$_3$
CF$_3$H
3a^{TMS}
4a
I.S.
TMSF
Ph$_3$SiF
Stage V
Stage IV
Stage III
Stage II
Stage I
chemical shift / ppm

Figure S1. ^{19}F NMR (376 Hz) spectra of the standard reaction of ethyl 2-hydroxybenzoate (0.2 M), $TMSCF_3$ (0.5 M), TBAT (0.015 M) at 300 K a) before initiation, b) during the reaction and c) upon completion. I.S. = internal standard, fluoronaphthalene d) Stacked ^{19}F NMR (376 Hz) spectra of ethyl 2-hydroxybenzoate (0.2 M), $TMSCF_3$ (0.5 M), TBAT (0.015 M) at 300 K. e) Partial temporal evolution of the major species in the reaction ethyl 2-hydroxybenzoate (0.2 M), $TMSCF_3$ (0.5 M) and TBAT (0.015 M) at 300 K. *= Likely to be 2,2,2-trifluoro-1-(2-trimethylsiloxyphenyl)ethanone on elimination of ethoxide from **8c**.

S1.3.2 Ethyl 3-fluoro-2-hydroxybenzoate ($2b^H$)

Presented below is the kinetic plot for the reaction of ethyl 3-fluoro-2-hydroxybenzoate (0.2 M) and $TMSCF_3$ (0.5 M) with TBAT (0.015 M) as the initiator and PhF (0.2 M) as the internal standard. The reaction was conducted at 300 K. The stacked ^{19}F NMR spectra showing the major species detected during monitoring are also presented.

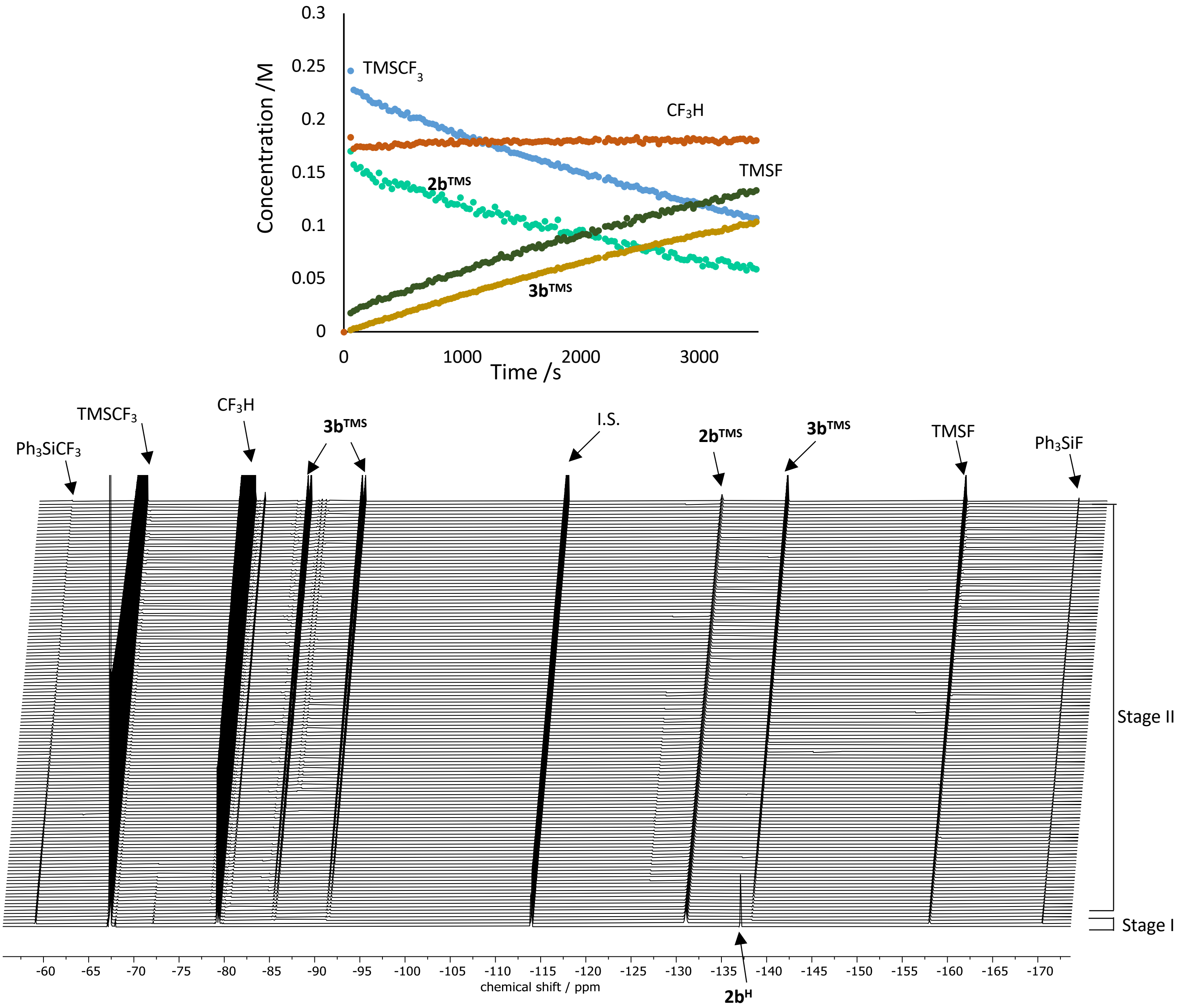

Figure S2. A) Kinetic plot obtained by ^{19}F NMR spectra of ethyl 3-fluoro-2-hydroxybenzoate (0.2 M) and $TMSCF_3$ (0.5 M) with TBAT (0.015 M). b) Stacked ^{19}F NMR (376 Hz) spectra of ethyl 3-fluoro-2-hydroxybenzoate, **$2b^H$** (0.2 M), $TMSCF_3$ (0.5 M), TBAT (0.015 M) at 300 K. The spectra show evolution of all major species in the reaction under these conditions.

S1.3.3 Ethyl 4-fluoro-2-hydroxybenzoate ($2c^H$)

Presented below is the kinetic plot for the reaction of ethyl 4-fluoro-2-hydroxybenzoate (0.2 M) and $TMSCF_3$ (0.5 M) with TBAT (0.015 M) as the initiator and PhF (0.1 M) as the internal standard. The reaction is conducted at 300 K. The stacked ^{19}F NMR spectra showing the major species detected during monitoring are also presented.

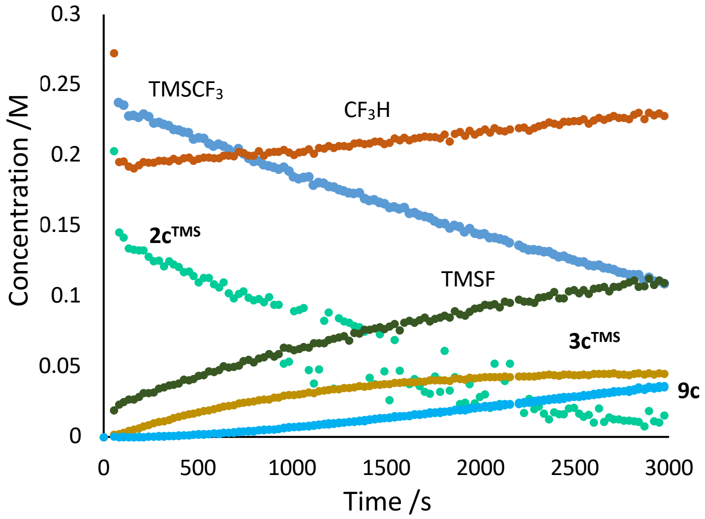

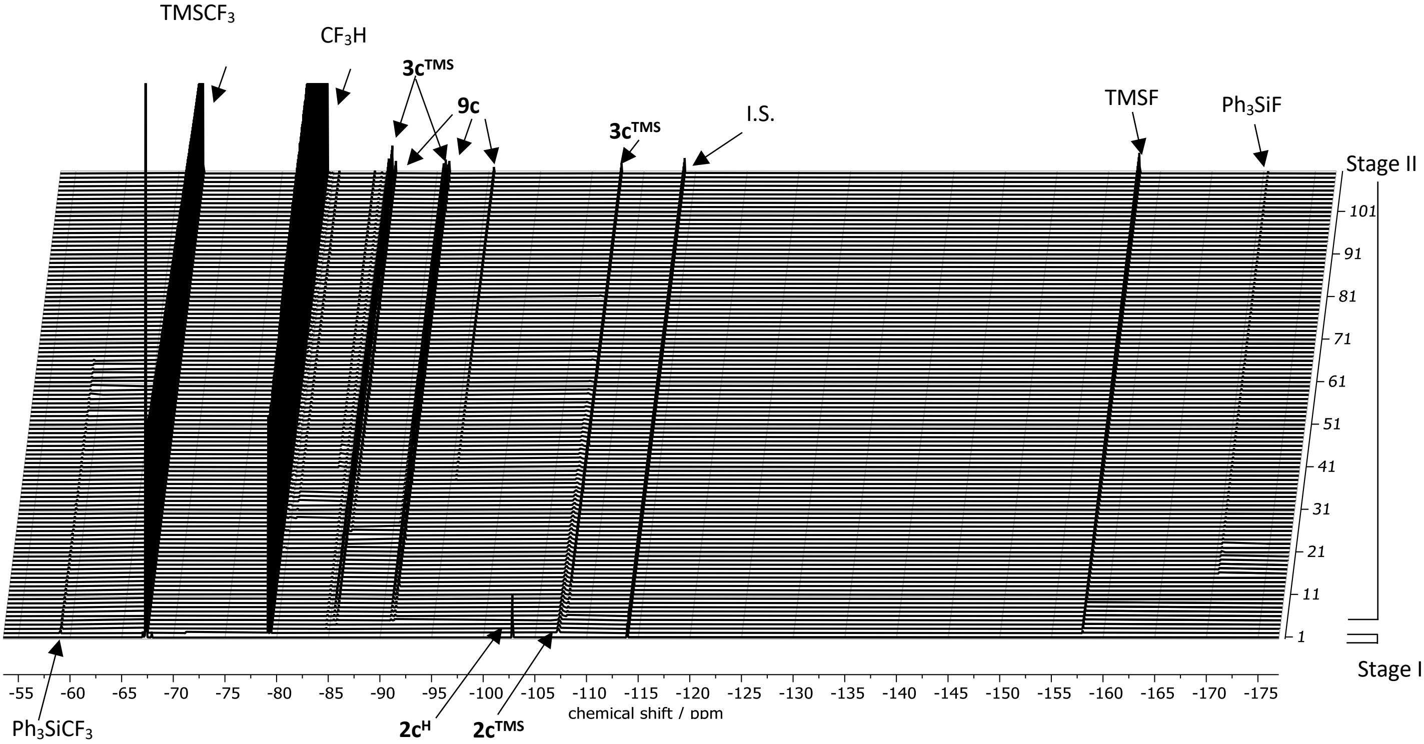

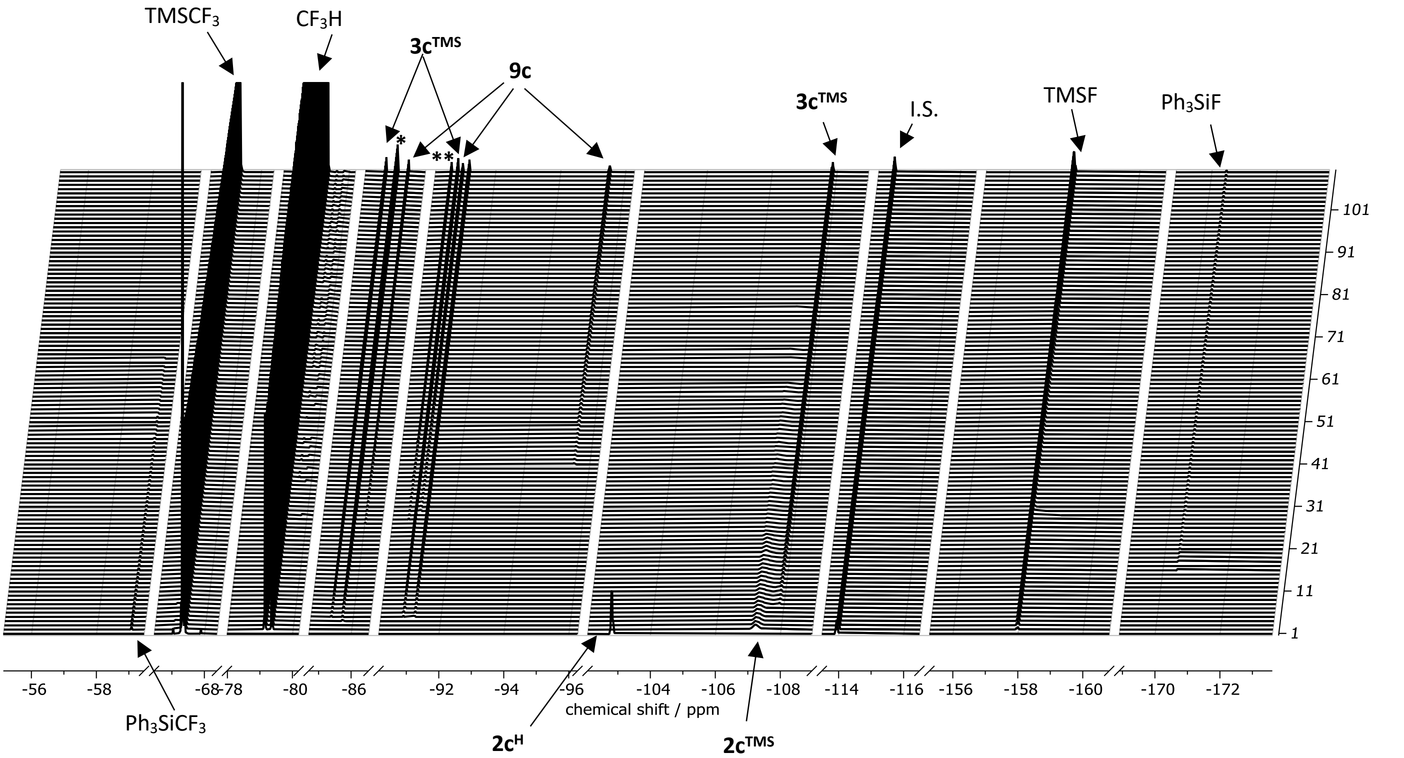

Figure S3. A) Kinetic plot obtained by ^{19}F NMR spectra of ethyl 4-fluoro-2-hydroxybenzoate (0.2 M) and $TMSCF_3$ (0.5 M) with TBAT (0.015 M). b) Stacked ^{19}F NMR (376 Hz) spectra of ethyl 4-fluoro-2-hydroxybenzoate, **2c^{H}** (0.2 M), $TMSCF_3$ (0.5 M), TBAT (0.015 M) at 300 K. The spectra show evolution of all species in the reaction under these conditions, c) Partial temporal evolution of all major species in the reaction. *= overlap of **3c^{TMS}** and **9c** signal. **= the doublet from both **3c^{TMS}** and **9c** overlap.

S1.3.4 Ethyl 5-fluoro-2-hydroxybenzoate ($2d^H$)

Presented below is the kinetic plot for the reaction of ethyl 5-fluoro-2-hydroxybenzoate (0.2 M) and $TMSCF_3$ (0.5 M) with TBAT (0.015 M) as the initiator and PhF (0.2 M) as the internal standard. The reaction is conducted at 300 K. The stacked ^{19}F NMR spectra showing the major species detected during monitoring are also presented.

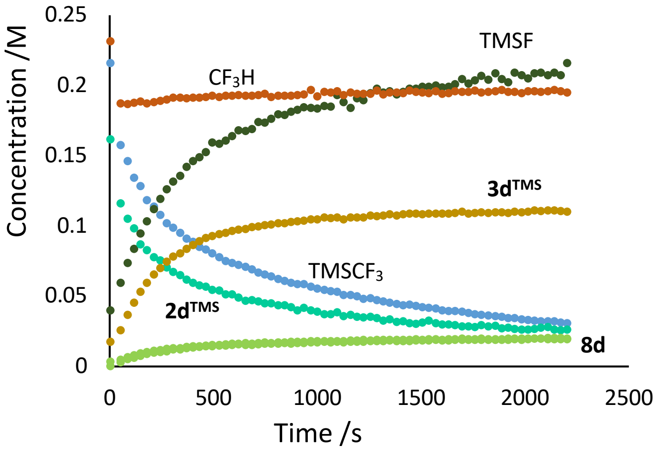

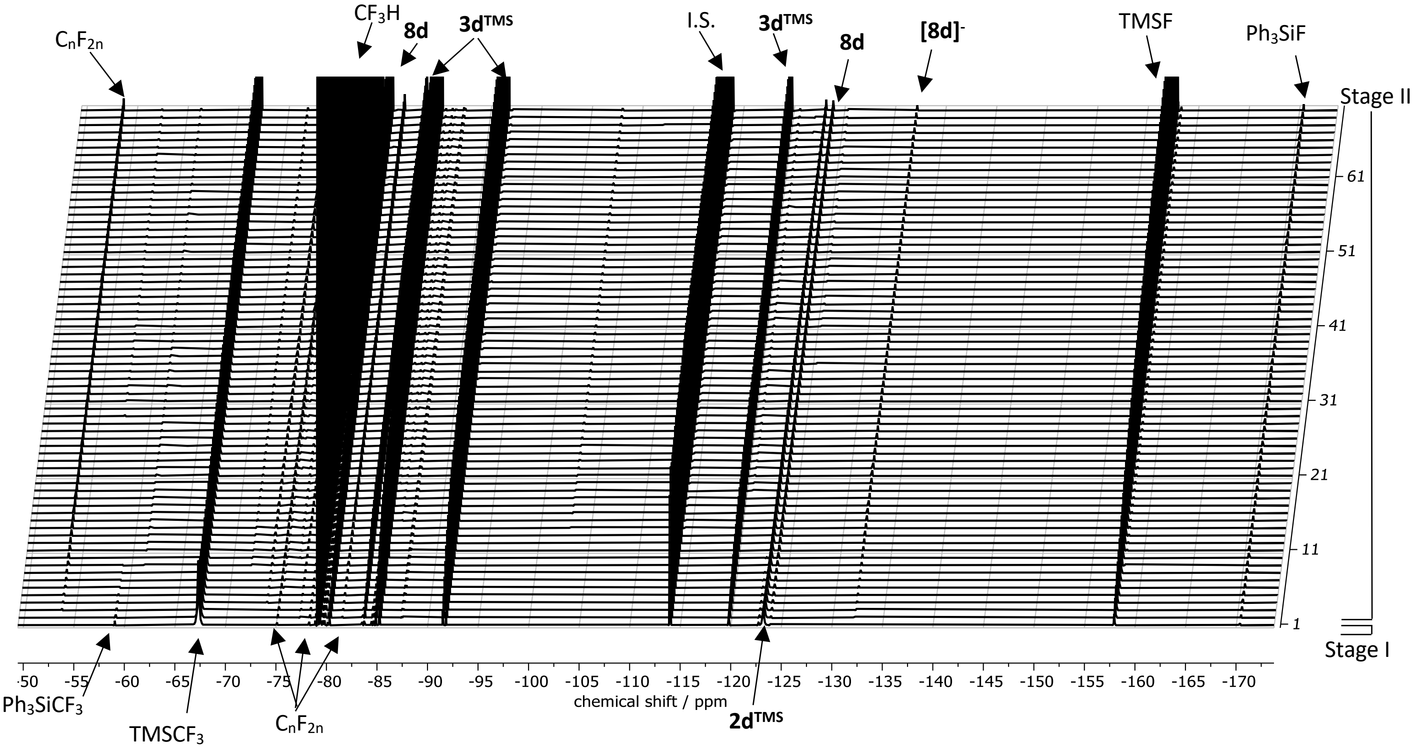

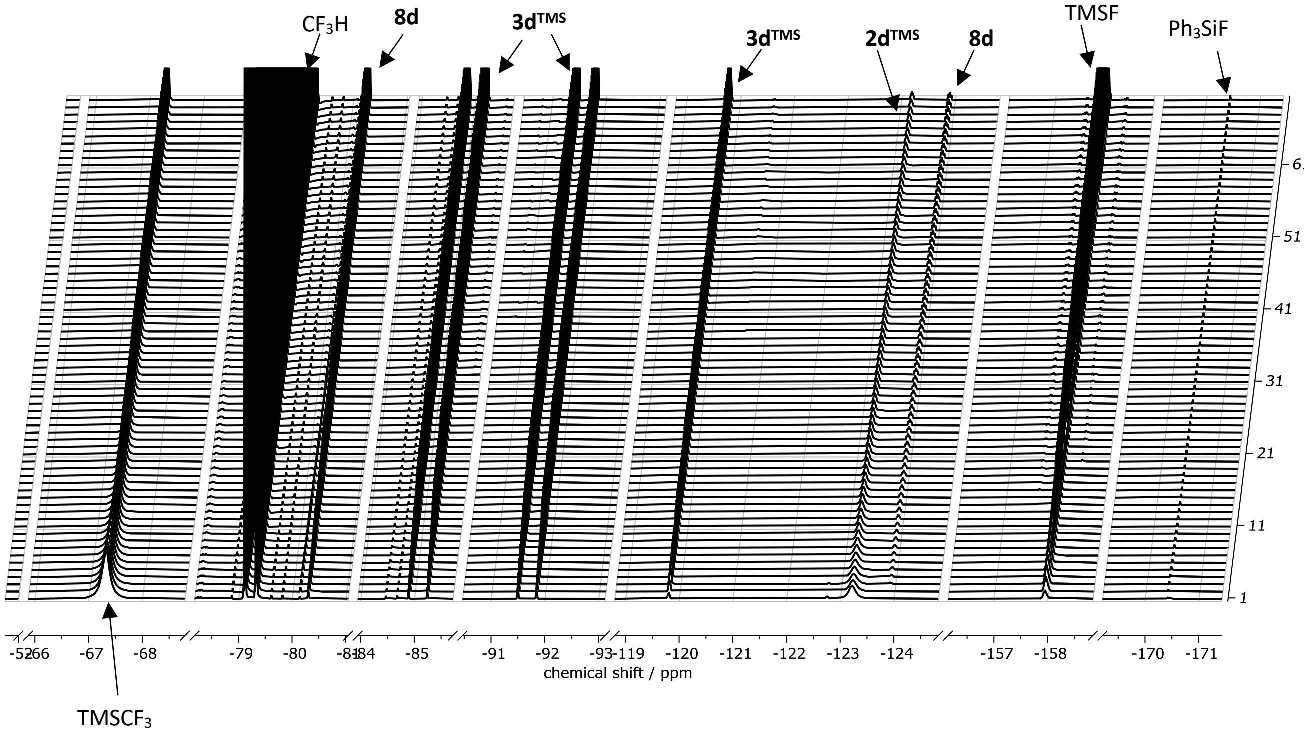

Figure S4. A) Kinetic plot obtained by ^{19}F NMR spectra of ethyl 5-fluoro-2-hydroxybenzoate (0.2 M) and $TMSCF_3$ (0.5 M) with TBAT (0.015 M). b) Stacked ^{19}F NMR (376 Hz) spectra of ethyl 5-fluoro-2-hydroxybenzoate, **2d^H** (0.2 M), $TMSCF_3$ (0.5 M), TBAT (0.015 M) at 300 K. The spectra show evolution of all species in the reaction under these conditions, c) Partial temporal evolution of all major species in the reaction.

S1.3.4 Ethyl 6-fluoro-2-hydroxybenzoate ($2e^H$)

Presented below is the kinetic plot for the reaction of ethyl 6-fluoro-2-hydroxybenzoate (0.2 M) and $TMSCF_3$ (0.5 M) with TBAT (0.015 M) as the initiator and PhF (0.2 M) as the internal standard. The reaction was conducted at 300 K. The stacked ^{19}F NMR spectra showing the major species detected during monitoring are also presented. An additional side product was identified when using **$2e^H$** which may be an additional ketal forming from **$3e^{TMS}$**, possibly accompanied by generation of TMSF. ^{29}Si NMR analysis (Section S7.4), suggested no significant quantity of other silicon-containing side products are formed.

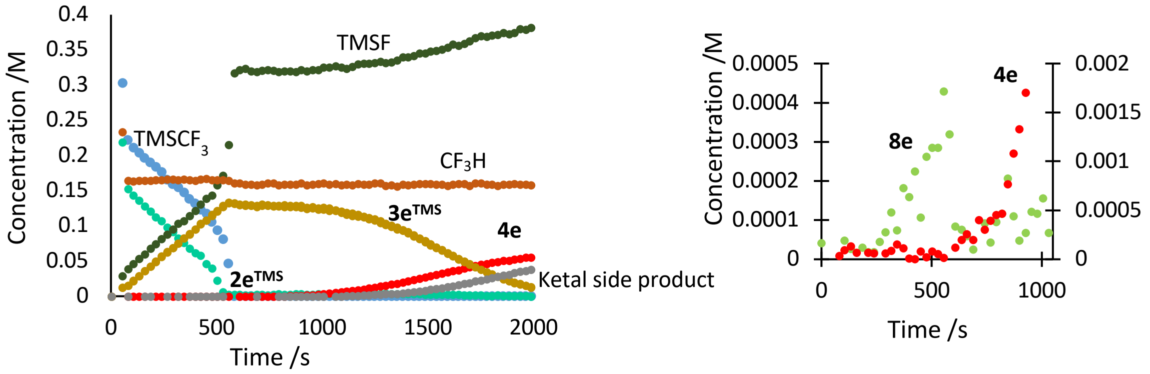

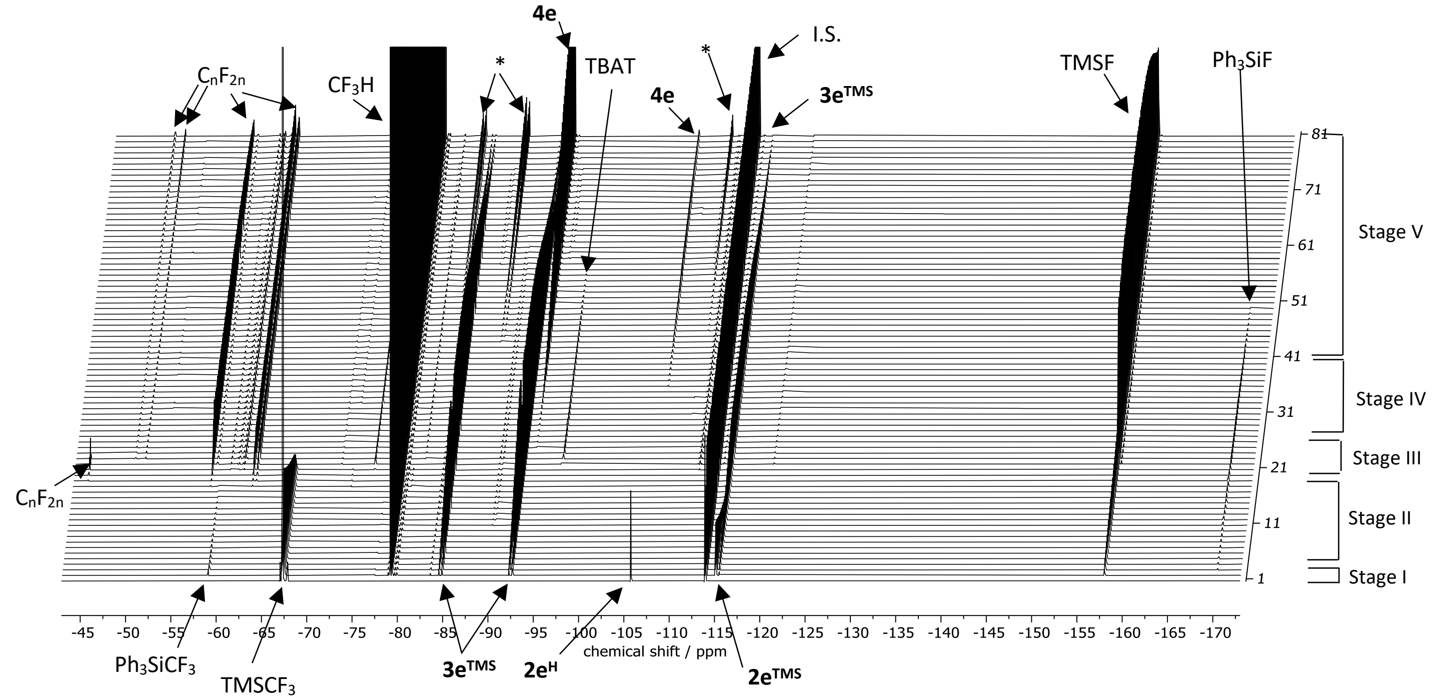

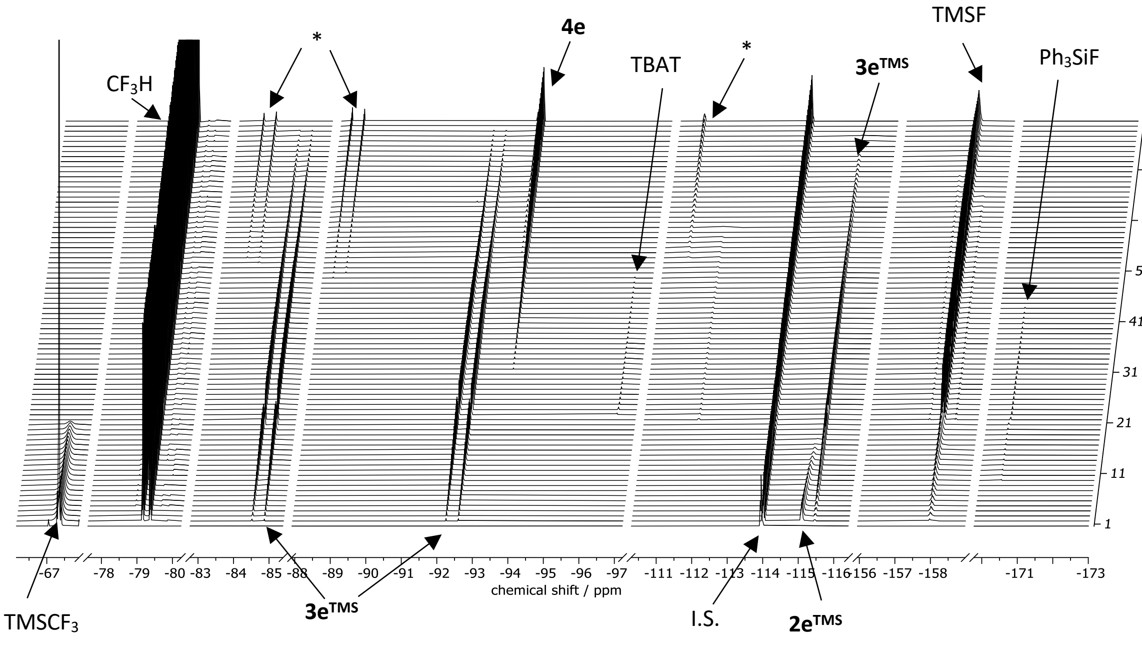

Figure S5. A) Temporal concentration evolution of the reaction of ethyl 6-fluoro-2-hydroxybenzoate (0.2 M), $TMSCF_3$ (0.5 M), and TBAT (0.015 M), monitored by ^{19}F NMR (376 Hz) at 300 K. Observation of CF_3 addition side product, **8e**. Product **4e** is plotted on the secondary axis to allow for observation of both species. b) Stacked ^{19}F NMR (376 Hz) spectra of ethyl 6-fluoro-2-hydroxybenzoate, **2e^H** (0.2 M), $TMSCF_3$ (0.5 M), TBAT (0.015 M) at 300 K. The spectra show evolution of all species in the reaction under these conditions, c) Partial temporal evolution of all major species in the reaction. An additional side product was identified using **2e^H** which may be an additional ketal forming from **3e^{TMS}**.

S1.4. Evidence for generation of siliconate [5⁻]

Previous work has established the equilibrium between the siliconate, **[5]**⁻ and TMSCF$_3$, **1**.[S1-3] However for reactions involving salicylates **2a-e**, the ^{19}F NMR signal corresponding to siliconate **[5]**⁻ peak is only observed when the reaction is cooled below 300 K. In the spectra below, the TBAT-initiated reaction of hydroxybenzoate **2e^{H}** with **1** was cooled from 300K to 275 K; held for a period, and then returned to 300 K. This confirmed the presence of the siliconate **[5]**⁻ in the reaction , further supported by the broadening of the signal arising from TMSCF$_3$, **1**.[S1-3]

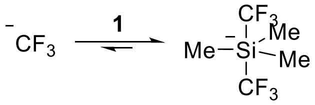

[5]⁻

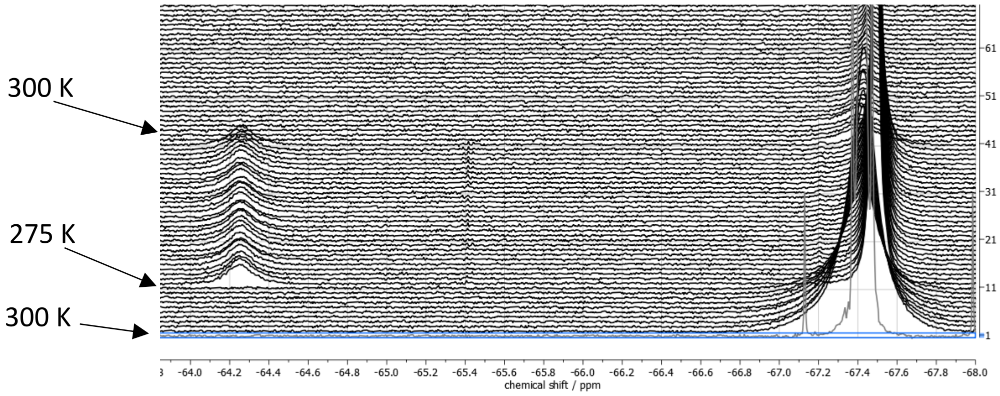

Figure S6. ^{19}F NMR (376 Hz) detection of TMSCF$_3$, **1**, and siliconate **[5]**⁻ in rapid equilibrium, and by implication, CF_3^-, on analysis of standard reaction starting with ethyl 6-fluoro-2-hydroxybenzoate **2e^{H}**. 10 spectra taken at 300 K, followed by 30 spectra taken at 275 K, followed 70 spectra at 300 K.

S2. Ketal formation: Variation of initial concentrations

The following reactions have been monitored by ^{19}F NMR, with a 15 s delay between each spectrum and thus data point. The concentration of ketal $\mathbf{3^{TMS}}$ during stages I-III is analyzed. All reactions have been carried out at 300 K unless otherwise specified. Initial standard conditions use the respective salicylate (0.2 M), $TMSCF_3$ (0.5 M) and TBAT (0.015 M)

S2.1. Initiator variation (ethyl 6-fluoro-2-hydroxybenzoate, 2e)

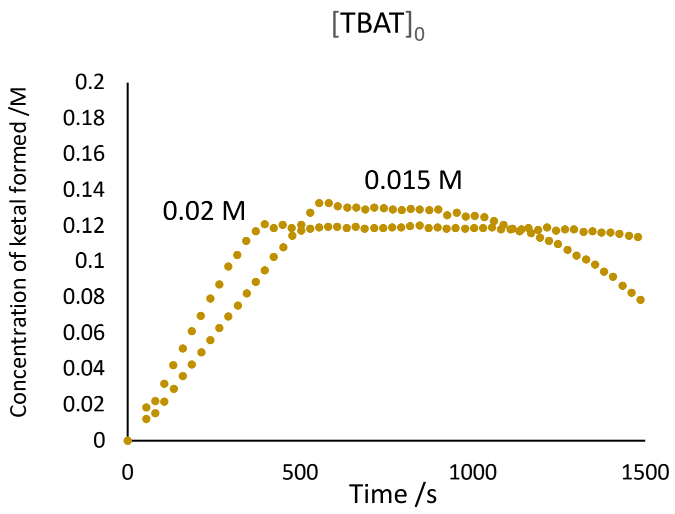

Figure S7. Temporal ketal evolution by varying initial concentrations of TBAT, the fluoride initiator, monitored by ^{19}F NMR (376 Hz) using ethyl 6-fluoro-2-hydroxybenzoate ($\mathbf{2e^H}$, 0.2 M).

S2.2. $TMSCF_3$ variation (ethyl 6-fluoro-2-hydroxybenzoate, 2e)

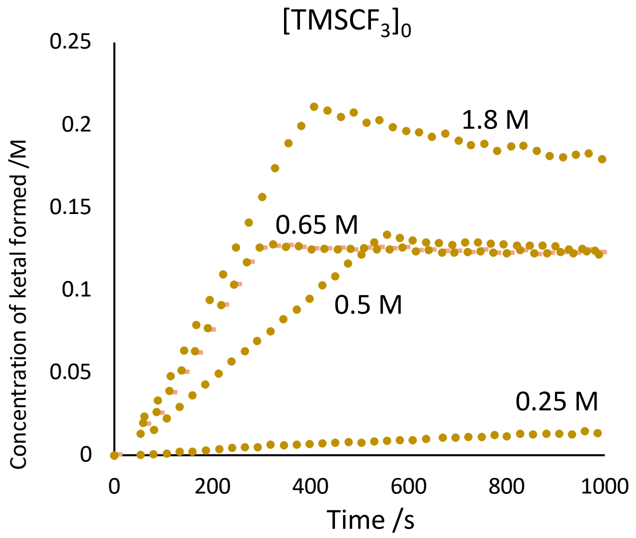

Figure S8. Temporal ketal evolution by varying initial concentrations of $TMSCF_3$ monitored by ^{19}F NMR (376 Hz) using ethyl 3-fluoro-2-hydroxybenzoate ($\mathbf{2e^H}$, 0.2 M).

S2.3. $TMSCF_3$ variation (ethyl 3-fluoro-2-hydroxybenzoate, 2b)

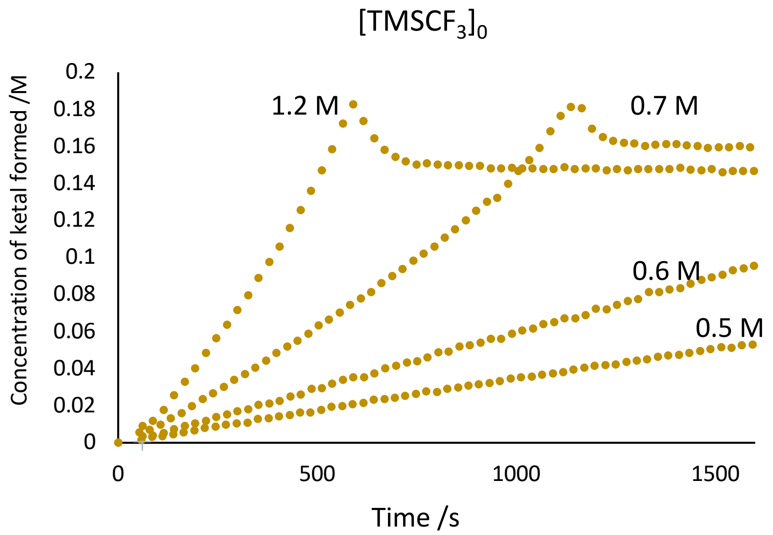

Figure S9. Temporal ketal evolution by varying initial concentrations of $TMSCF_3$ monitored by ^{19}F NMR (376 Hz) using ethyl 3-fluoro-2-hydroxybenzoate (**2b**^{H}, 0.2 M).

S2.4. $TMSCF_3$ variation (ethyl 4-fluoro-2-hydroxybenzoate, 2c)

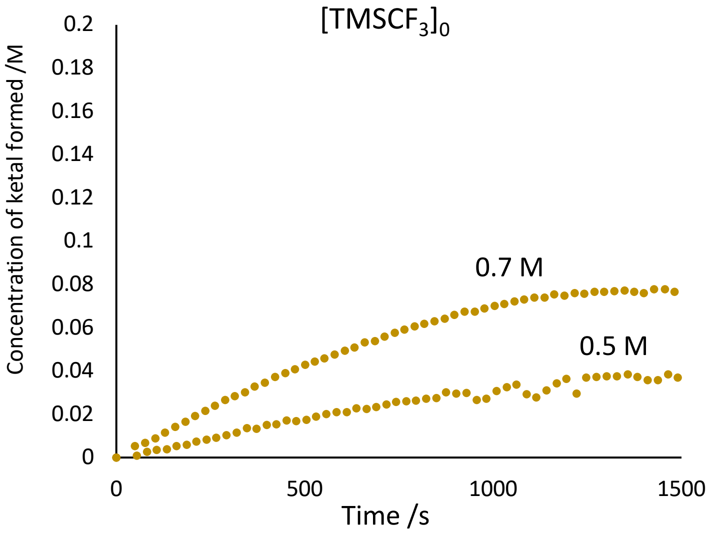

Figure S10. Temporal ketal evolution by varying initial concentrations of $TMSCF_3$ using (**2c**^{H}, 0.2 M) monitored by ^{19}F NMR (376 Hz). No ketal intermediate was generated when using 0.25 M $TMSCF_3$.

S2.5. Phenol substrate variation (ethyl 6-fluoro-2-hydroxybenzoate, 2e)

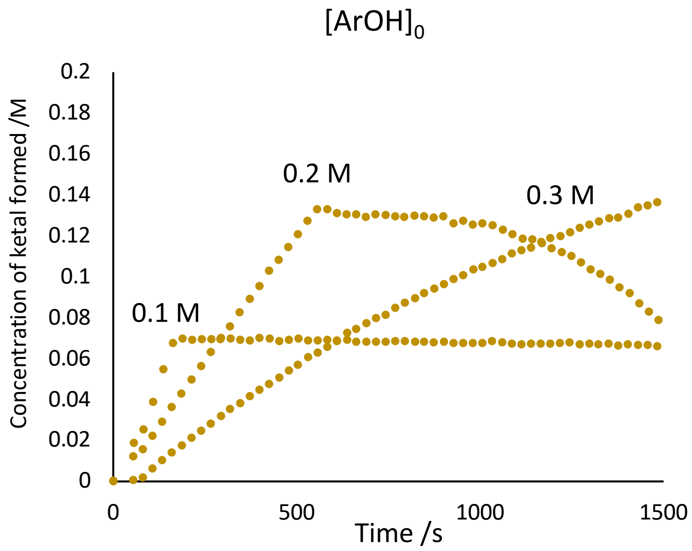

Figure S11. Temporal ketal evolution by varying initial concentrations of salicylate using (**2e**^{H}) monitored by ^{19}F NMR (376 Hz).

S3. Evidence for Generation of Salicylate TMS ethers (2^{TMS}) – Stage I

S3.1. Evidence for Salicylate TMS ethers (2^{TMS}) in Equilibrium with Salicylate Anions [2^-].

The ^{19}F NMR signal assigned to TMS ethers (**2^{TMS}**) detected during in situ reaction monitoring, changes in chemical shift as the reaction progresses. This indicates that a dynamic equilibrium may be present. The TMS ether forms after the reaction is initiated using the phenolic substrate. Figure S12 employs ethyl 6-fluoro-2-hydroxybenzoate (**$2e^H$**) to illustrate this. Using the corresponding TMS ether (**$2e^{TMS}$**) as the starting material, also results in peak migration and broadening after initiation**.**

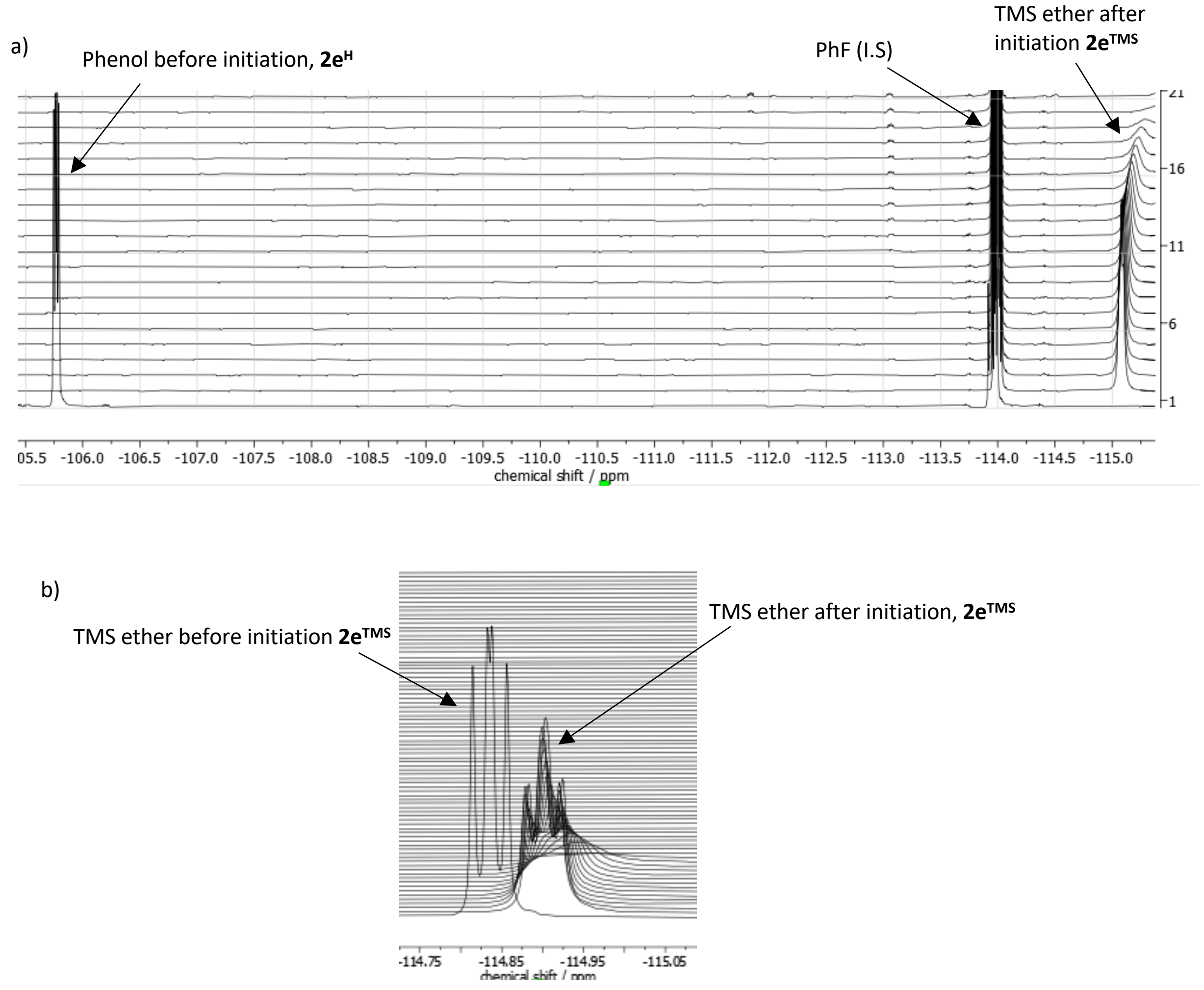

Figure S12. a) TMS ether, **$2e^{TMS}$** formation using 6-fluoro-2-hydroxybenzoate, **$2e^H$**, as the substrate under standard conditions, $TMSCF_3$ (0.5 M), TBAT (0.015 M) monitored by ^{19}F NMR (376 Hz), b) 6F-TMS ether **$2e^{TMS,}$** used directly in the reaction. Both spectra show the broadening and shifting of the TMS ether peak. PhF = fluorobenzene, I.S = internal standard.

S3.2. Use of TMS ether (2a,d,e^{TMS}) as the substrate

Using the corresponding TMS ethers of ethyl 6-fluoro-2-hydroxybenzoate, ethyl 2-hydroxybenzoate and ethyl 5-fluoro-2-hydroxybenzoate, the reaction proceeded to the ketal intermediate, the same intermediate formed as when starting the respective phenols, but without CF_3H generation.

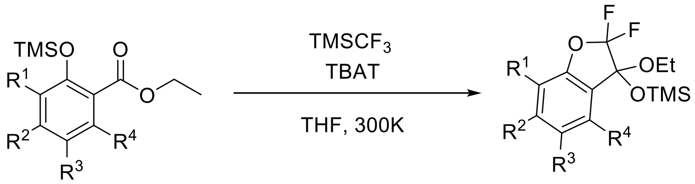

2a^{TMS} R^1= H, R^2= H, R^3= H, R^4=H
2d^{TMS} R^1= H, R^2= H, R^3= F, R^4=H
2e^{TMS} R^1= H, R^2= H, R^3= H, R^4=F

3a^{TMS} R^1= H, R^2= H, R^3= H, R^4=H
3d^{TMS} R^1= H, R^2= H, R^3= F, R^4=H
3e^{TMS} R^1= H, R^2= H, R^3= H, R^4=F

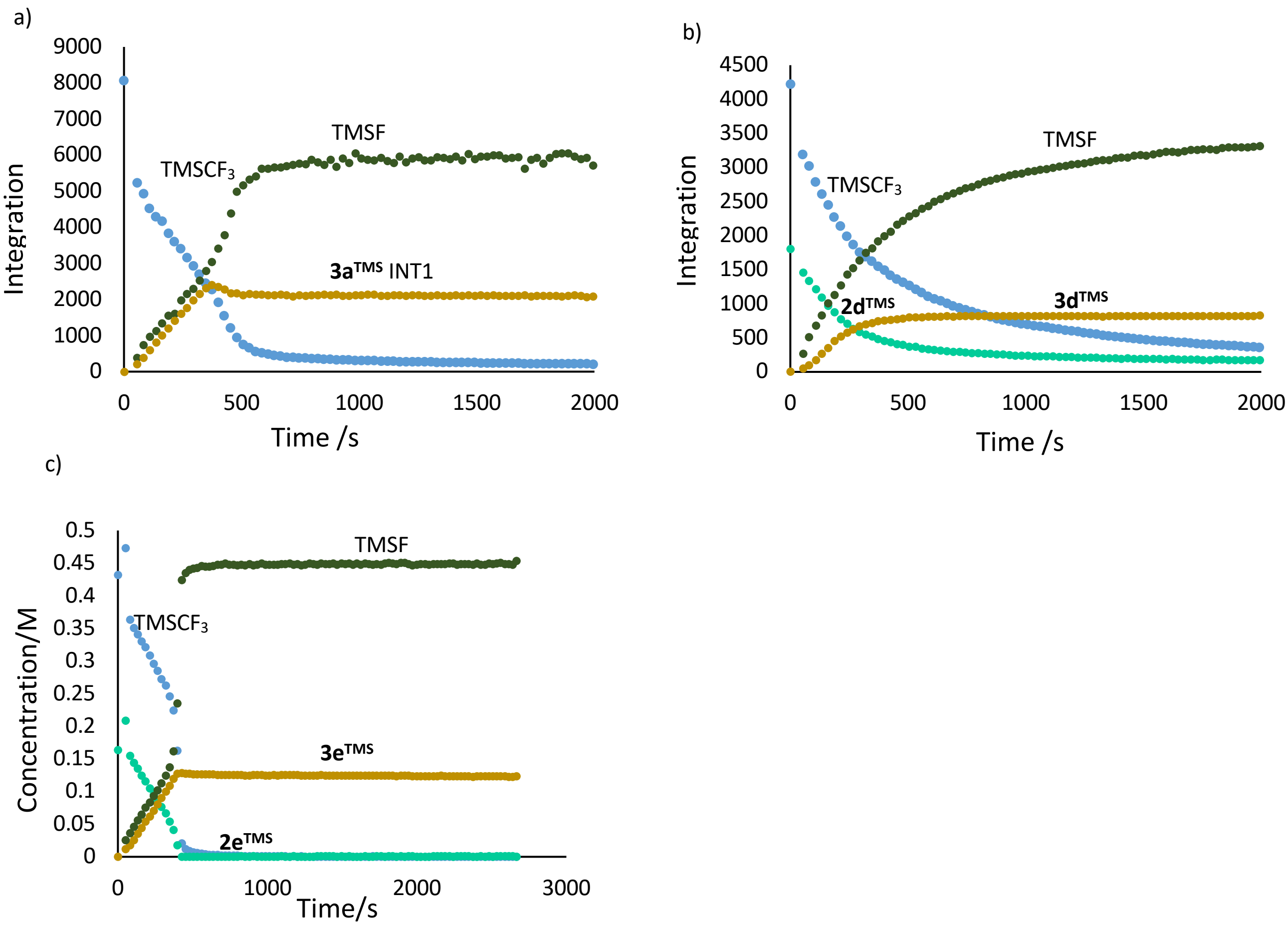

Figure S13. Kinetic profiles of the reactions of the TMS ethers of a) ethyl salicylate, **2a^{TMS}**, b) ethyl 5-fluoro-2-hydroxybenzoate, **2d^{TMS}**, c) ethyl 6-fluoro-2-hydroxybenzoate, **2e^{TMS}** monitored by ^{19}F NMR (376 Hz). All reactions are initiated at **2^{TMS}** (0.2 M), $TMSCF_3$ (0.5 M) and TBAT (0.015 M).

S3.3. $TMSCF_3$ variation (TMS ether, $2a^{TMS}$)

The following reactions have been run using pre-synthesised TMS ether (**2a**^{TMS}) derived from ethyl salicylate (**2a**^{H}). These reactions were monitored by ^{19}F NMR at 300 K, with a 15 s delay between each spectrum and thus data point. Increasing $TMSCF_3$ concentration leads to an increasing rate of formation of the ketal intermediate towards a point of saturation, mirroring the kinetic data obtained from reactions carried out with the corresponding phenol, **2a**^{H}.

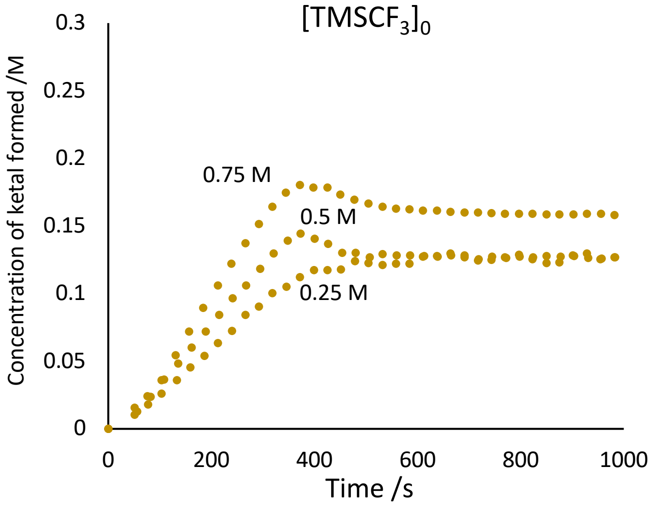

Figure S14. Temporal ketal evolution by varying initial concentrations of $TMSCF_3$. Reactions monitored by ^{19}F NMR (376 Hz) using TMS ether **2a**^{TMS} (0.2 M), TBAT (0.015 M) at 300 K.

S3.4. Titration Data

To detect the ^{19}F NMR signal corresponding to the salicylate anion, [**2**$^-$], ethyl 5-fluoro-2-hydroxybenzoate (**2d**^{TMS}) was titrated with a stoichiometric quanity of TBAT. The ^{19}F NMR signal corresponding to [**2d**$^-$] initially appears at -131.7 ppm, and migrates to -137.3 ppm as further TBAT is added in 16 mg (0.15 equiv.) increments, until 1.1 equivalents total had been added.

^{19}F NMR signals corresponding to unidentified side products, not present in the initial spectra, were also noted. In addition, the signal corresponding to [**2d**$^-$] is broad throughout the titration, despite no $TMSCF_3$ being added, leading to the conclusion that there is potentially an additional equilibrium with TBAT.

Quantification was challenging due to the broad nature of the signals peak, which impacted phase and baseline correction of the spectra. Imperfect baseline correction results in a reduced integral for the phenoxide, [**2d**$^-$]. Nonetheless, the titration demonstrates that the chemical shift of phenoxide, [**2d**$^-$], migrates in the same direction as that observed during the reaction monitoring when $TMSCF_3$ is present.

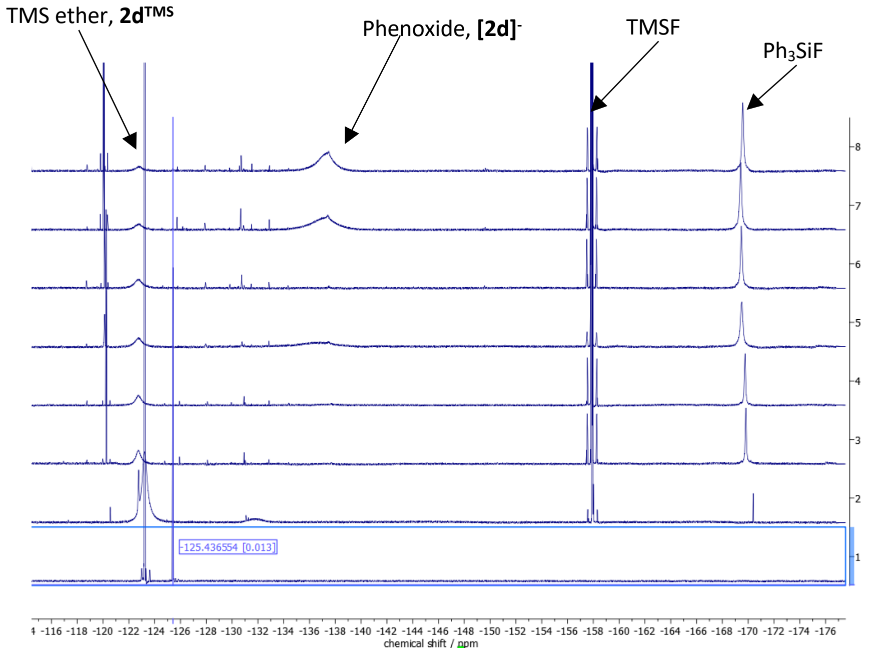

Figure S15. Stacked spectra of a titration of ethyl 5-fluoro-2-hydroxybenzoate, **2d**, with stoichiometric amount of TBAT monitored by ^{19}F NMR (376 Hz), adding equal increments until 1.2 equivalents has been added, to allow for observation of the phenoxide peak, **[2d]**$^-$.

S4. Evidence for CF_2 Generation – Stage II

S4.1. Carbene trapping by alkene 6

To probe for transient CF_2, the standard reaction conditions of $TMSCF_3$ (0.5 M) and TMS ether **2a^{TMS}** (0.2 M) were run with alkene **6** present in excess (0.3 M) and initiated with TBAT (0.015 M).

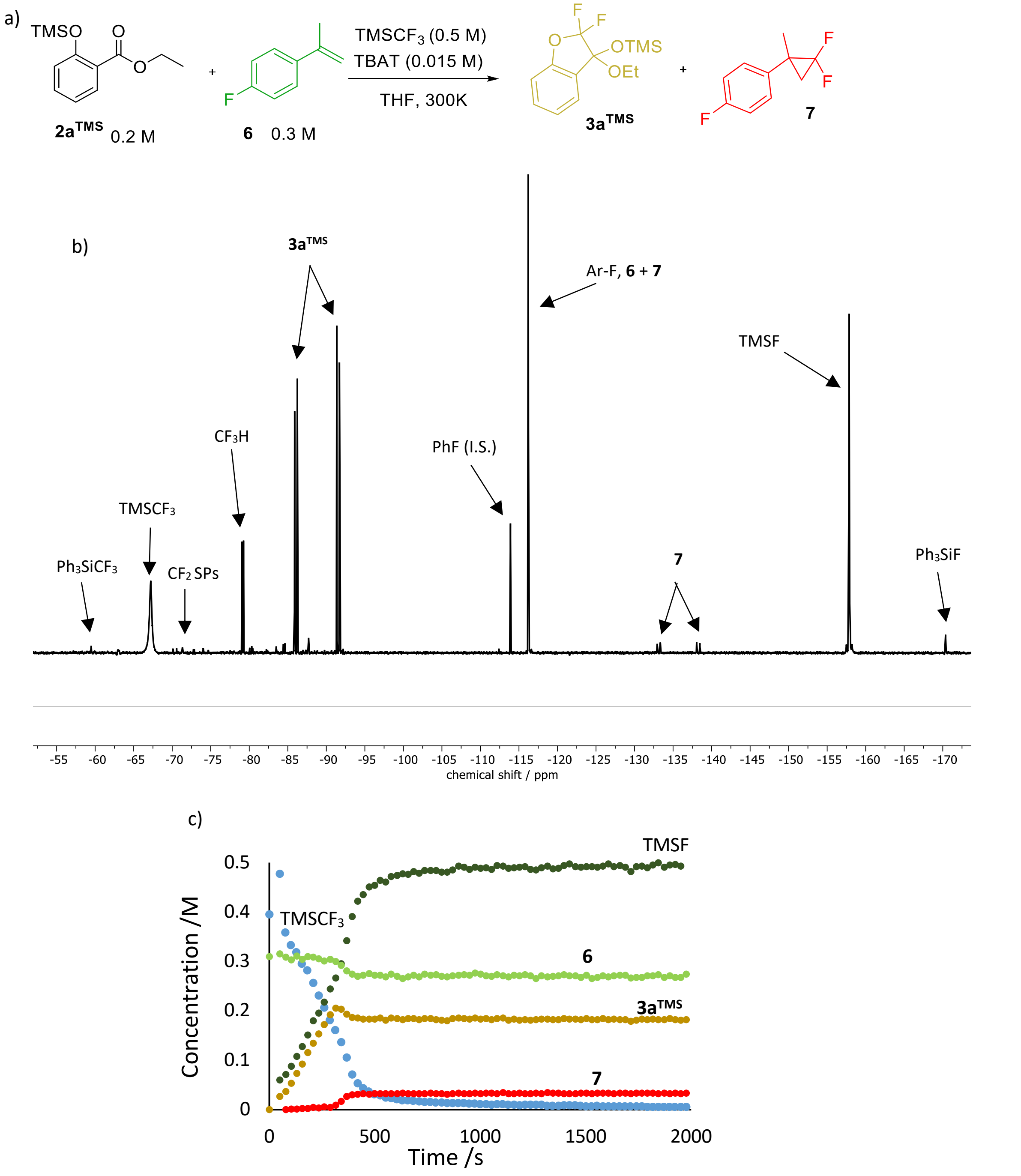

Figure S16. a) Reaction scheme. b) Spectrum at the end of the reaction. c) Kinetic plot from *in situ* ^{19}F NMR (376 Hz), showing carbene trapping by alkene **6** is only significant during the phase when ketal **3a^{TMS}** generation plateaus and TMSF accelerates (the stage II-III transition). The ^{19}F NMR signal from the aryl fluorine in alkene **6** (-116.2 ppm) overlaps with the aryl fluorine signal of the product **7**. However the geminal difluoro unit in difluorocyclopropane **7** (-133.2, 138.2 ppm) is clearly evident.

S4.2. Reactions of Salicylates 2^H with $TESCF_3$

The reactions of ethyl 4-, 5-, - and 6--fluoro-2-hydroxybenzoates **2c**^{H}, **2d**^{H} and **2e**^{H} with $TESCF_3$ demonstrate that when the silane reagent is more biased to CF_3 anion reactivity over CF_2 generation, the rate of ketal generation is strongly attenuated. CF_3 addition to the ester is observed in all reaction.

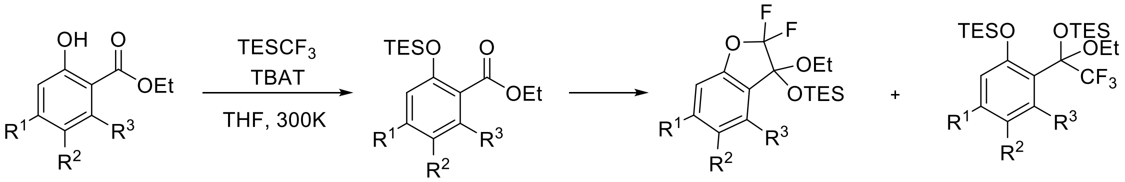

2c^{H} R^1=F, R^2,R^3 =H
2d^{H} R^1,R^3=H, R^2=F
2e^{H} R^1,R^2=H, R^3 =F

2c^{TES} R^1=F, R^2,R^3 =H
2d^{TES} R^1,R^3=H, R^2=F
2e^{TES} R^1,R^2=H, R^3 =F

3c^{TES} R^1=F, R^2,R^3 =H
3d^{TES} R^1,R^3=H, R^2=F
3e^{TES} R^1,R^2=H, R^3 =F

8c^{TES} R^1=F, R^2,R^3 =H
8d^{TES} R^1,R^3=H, R^2=F
8e^{TES} R^1,R^2=H, R^3 =F

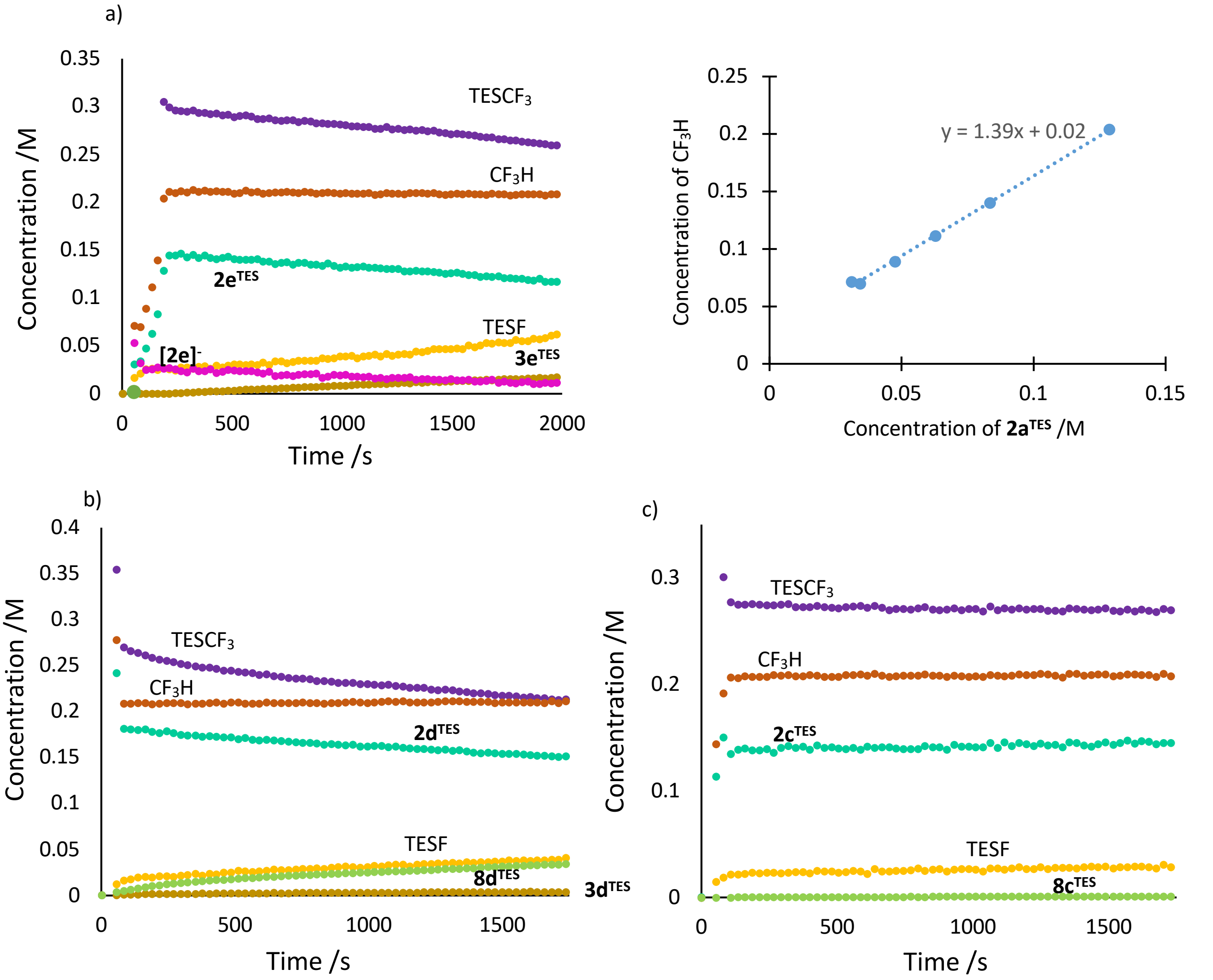

Figure S17. a) Kinetic profile of the reaction of ethyl 6-fluoro-2-hydroxybenzoate (**2e**^{H}, 0.2 M), $TESCF_3$ (0.5 M), and TBAT (0.015 M), and concentration vs. concentration plot showing formation of CF_3H and **2a**^{TES}. b) Kinetic profile of the reaction of ethyl 5-fluoro-2-hydroxybenzoate (**2d**^{H}, 0.2 M), $TESCF_3$ (0.5 M), and TBAT (0.015 M) c) Kinetic profile of the reaction of ethyl 4-fluoro-2-hydroxybenzoate (**2c**^{H}, 0.2 M), $TESCF_3$ (0.5 M), and TBAT (0.015 M). All reactions monitored by ^{19}F NMR (376 Hz).

S4.3. Reaction of Salicylate TMS Ether with $TESCF_3$

The reaction of TMS ether **2e**^{TMS} (0.2 M), with $TESCF_3$ (0.5 M), was monitored by ^{19}F NMR (376 Hz) at 300 K and found to generate two ketal intermediates: **3e**^{TMS} and **3e**^{TES} in a process that was considerably faster than reactions involving only TES species, Section S4.2. Endogenous $TMSCF_3$ is detected in the first data point, suggesting indirect exchange between $TESCF_3$ and TMS ether, mediated by CF_3 anion(oid), and thus also generating **2e**^{TES}. Both TMSF and TESF are detected. Accelerated TESF generation is not observed in the reactions of $TESCF_3$ alone (Section S4.2), but only when $TMSCF_3$ is present, Figure S18.

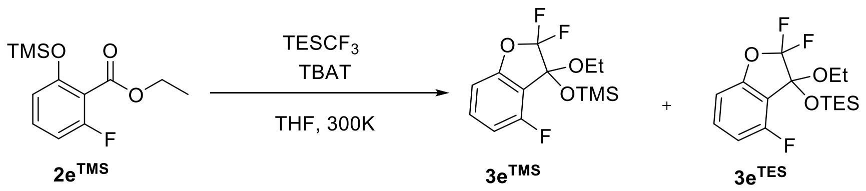

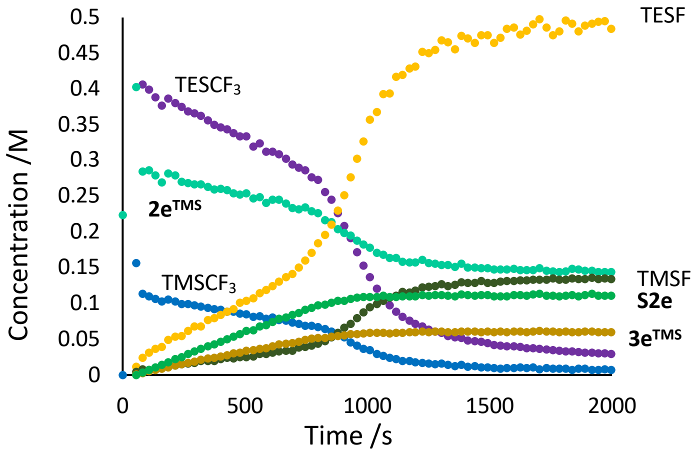

Figure S18. a) Kinetic profile of the reaction of TMS ether **2e**^{TMS} (0.2 M), $TESCF_3$ (0.5 M), and TBAT (0.015 M) monitored by ^{19}F NMR (376 Hz) at 300 K. Formation of both **3e**^{TMS} and **3e**^{TES}, and stage III acceleration is observed, unlike reactions using $TESCF_3$ alone (Section S4.2).

S5. Accelerating rate of TMSF generation – Stage III

S5.1. Transient dynamic line broadening

Line broadening is observed in ^{19}F NMR signals of the ketal intermediates (**3**$^{\mathbf{TMS}}$) during stage III when there is an accelerated rate of TMSF generation. Figure S19 shows an example for reaction of **2e**$^{\mathbf{H}}$. The broadening of the two ^{19}F NMR doublets arising from the CF_2 unit in **3e**$^{\mathbf{TMS}}$ indicates dynamic equilibrium with a minor species, e.g., [**3**$^{-}$]. The slight mutual contraction of the chemical shift separation between the 'AB' system may also indicate further equilibrium of [**3**$^{-}$] with its ring-opened form (i.e. the primary product from addition of CF_2 to [**2**$^{-}$]) which, through anion inversion, would result in overall equilibration of the diastereotopic geminal fluorine atoms in **3e**$^{\mathbf{TMS}}$.

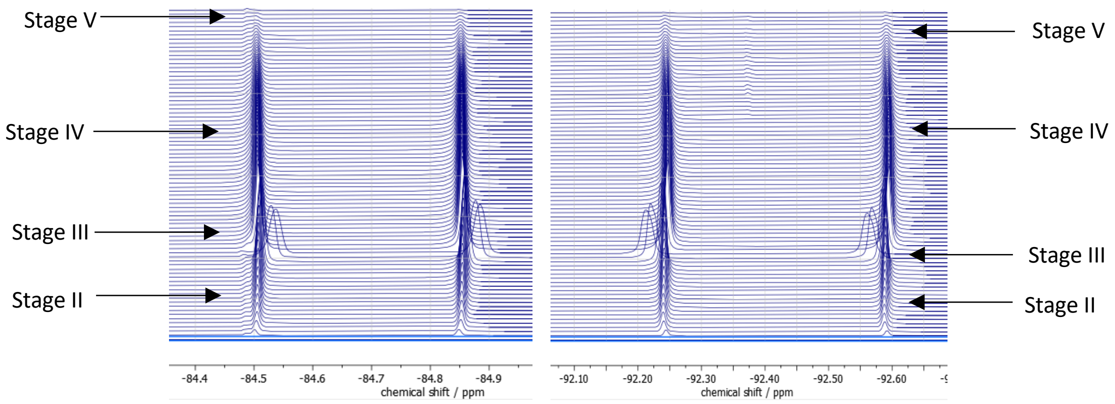

Figure S19. Dynamic line broadening of the intermediate ketal **3e**$^{\mathbf{TMS}}$ evident in the *in situ* ^{19}F NMR spectra (376 Hz) during accelerated TMSF generation (stage III).

S5.2. Comparison of rate of accelerated TMSF generation in stage III, across substrates 2a-e.

During all of the reactions of the salicylates $\mathbf{2^H}$ and $\mathbf{2^{TMS}}$, employing $TMSCF_3$, acceleration in the generation of TMSF (and TESF, Section S4.3) is observed at stage III when the rate of formation of the ketal intermediate ($\mathbf{3^{TMS}}$) plateaus. The limiting rate of generation of CF_2 + TMS (i.e. $k_F[TBAT]_0/K_2$) was estimated from the rate of TMSF generation using alkene **6** in a standard difluorocyclopropanation reaction.[S3]

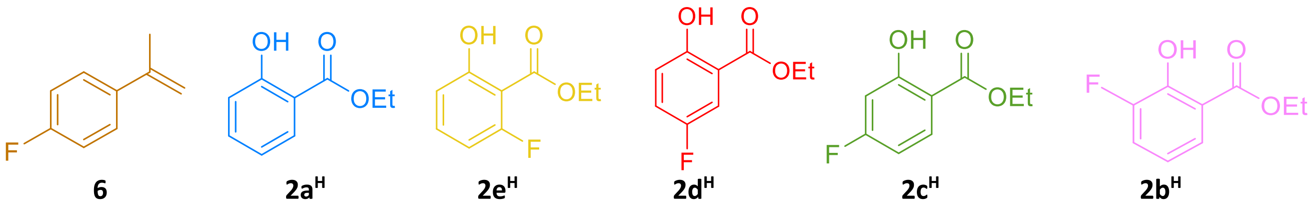

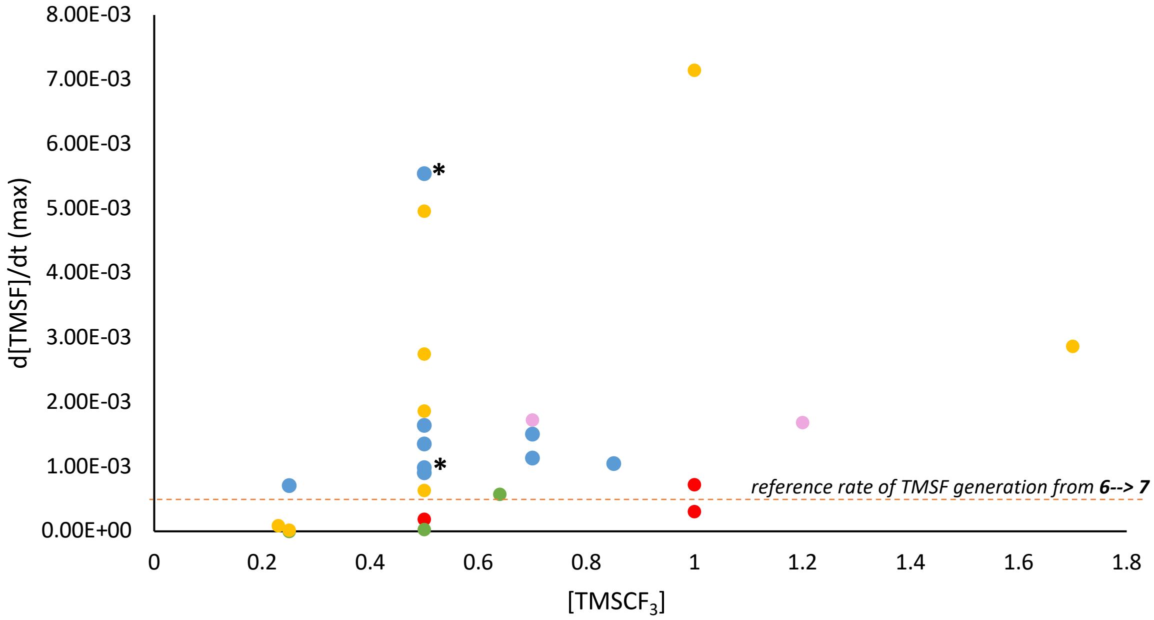

Figure S20. Estimated maximum rates of TMSF generation during stage III at different initial $TMSCF_3$ concentrations for salicylates $\mathbf{2a\text{-}e^H}$, all initially 0.2 M, except the two datapoints indicated (*) which were run at 0.1 M $[\mathbf{2a^H}]_0$. While there is no simple correlation, it is evident that all of the ketals ($\mathbf{3a\text{-}e^{TMS}}$) result in the rate of TMSF generation exceeding that of the background process that generates CF_2 + TMS from $[CF_3]^-$ + $TMSCF_3$, which has a saturation value of $k_F[TBAT]_0/K_2$.[S3] Ketals $\mathbf{3a^{TMS}}$ and $\mathbf{3e^{TMS}}$ results in the most enhanced rate of TMSF generation. The rates were estimated by taking the maximum gradient between pairs of data points in temporal concentration plots for TMSF determined by *in situ* ^{19}F NMR (376 Hz) analysis. The dashed line is the rate of TMSF generation rate when using alkene **6** to trap CF_2 (as difluorocyclopropanation product **7**) and serves as the reference for acceleration over the background process.

S6. Evidence for CF_3 addition in stage II

S6.1. Reactions of reference esters

Evidence for CF_3 addition to the ester of the TMS ethers $\mathbf{2}^{\mathbf{TMS}}$ was obtained by *in situ* NMR analysis, together with comparison of the reactions of related species. The CF_3 addition intermediates were not isolated. Ethyl benzoate (**S1a**) and ethyl 2-methoxybenzoate (**S1b**) were subject to standard reaction conditions, to gain approximate ^{19}F NMR chemical shift references for CF_3 addition as -81.0 and –80.7 ppm respectively; these values are similar to those found for CF_3 addition to ketones and aldehydes.[1S]

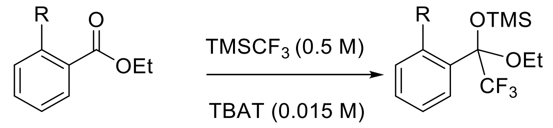

S1a R= H
S1b R= OMe

S2a R= H
S2b R= OMe

(A)

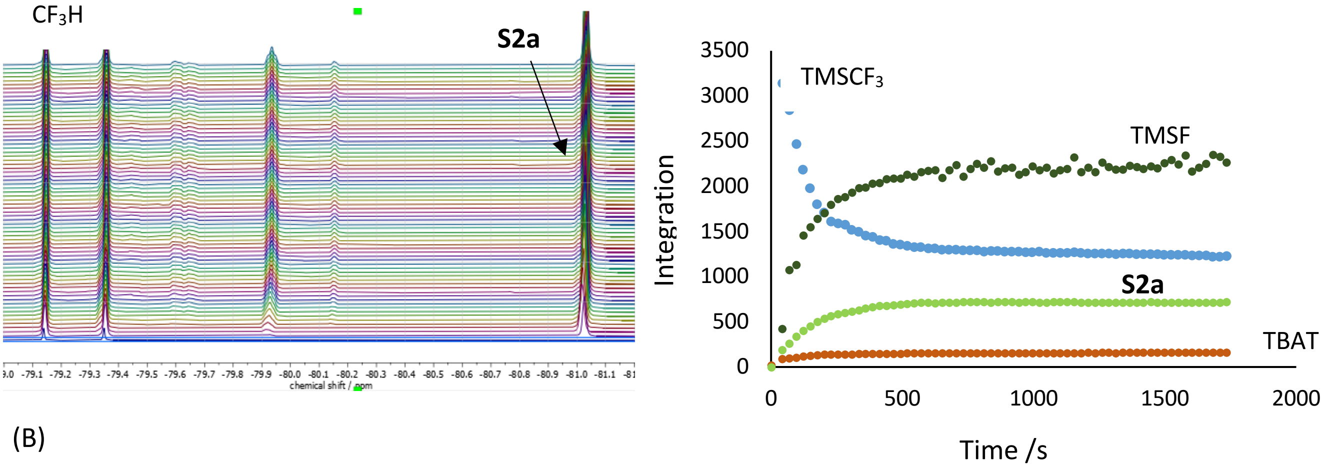

(B)

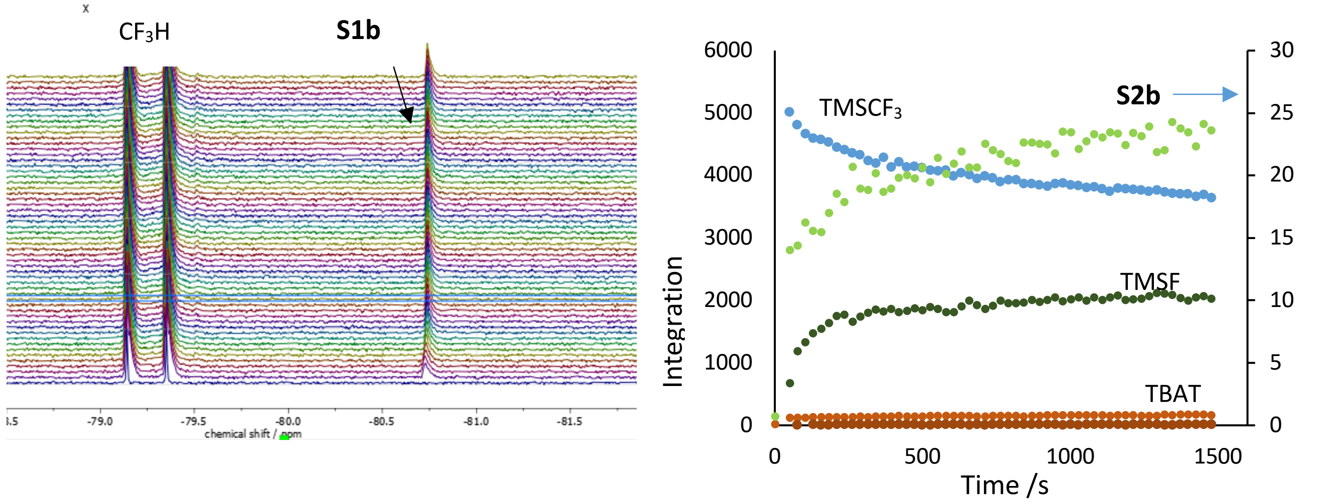

Figure S21. A) Stacked ^{19}F NMR (376 Hz) spectra showing CF_3 addition (-81.0 ppm), and kinetic profiles of reaction of ethyl benzoate (**S1a**, 0.2 M), $TMSCF_3$ (0.5 M) and TBAT (0.015 M). B) stacked spectra showing CF_3 addition (-80.7 ppm), and kinetic profile for (**S1b**, 0.2 M), $TMSCF_3$ (0.5 M) and TBAT (0.015 M). Integration of **S2b** is on secondary y-axis.

Based on these chemical shifts, the ^{19}F NMR data for stage II reactions of TMS ethers $\mathbf{2^{TMS}}$ suggests that CF_3 addition occurs in some cases, with an evident effect that substitution *ortho* to the ester markedly attenuates the CF_3 anion addition. This is also supported by the more extensive generation of **S2a** compared to **S2b**, see Figure S21. The TMSO group is expected to exert a similar or greater shielding effect to MeO, and therefore attenuate the rate of anion addition to the ester. When ethyl 5-fluoro-2-hydroxybenzoate, $\mathbf{2d^{H}}$, is used as the substrate, Figure S22, CF_3 addition (to generate **8d**) is promoted. The overlay of the weighted integrations of the CF_3 group and the aromatic fluorine (Figure S22b) support the assignment.

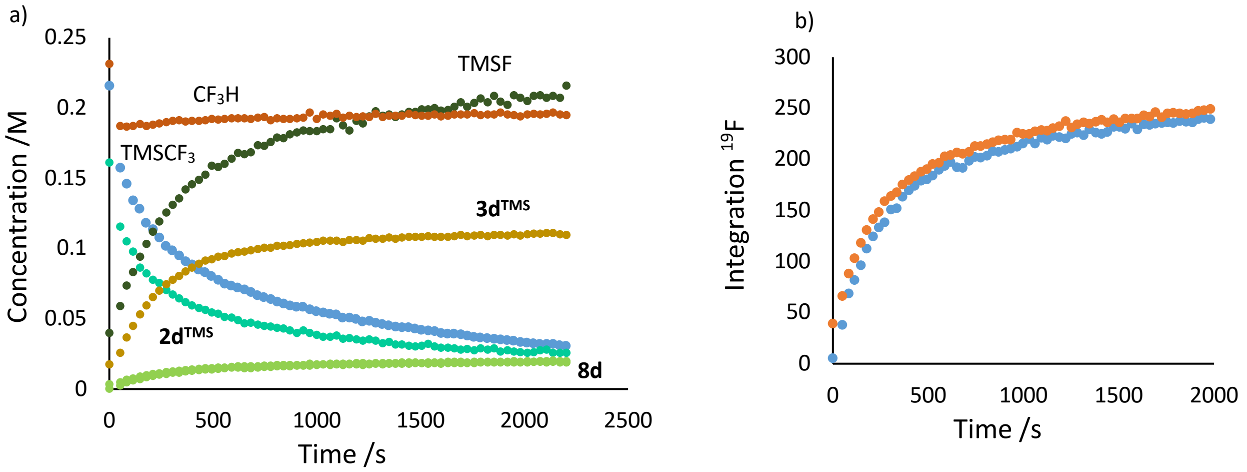

Figure S22. a) Kinetic profile of reaction under standard condition using ethyl 5-fluoro-2-hydroxybenzoate , $\mathbf{2d^{H}}$, as the substrate monitored by ^{19}F NMR (376 Hz). b) Overlap of integration of CF_3 peak (weighted by 1/3) and aromatic fluorine peak, supporting assignment of CF_3 and aromatic F being within the same molecule (**8d**).

S7. Difluoro coumaranone (4) formation – Stage V

S7.1. CO_2 addition

To investigate the influence of the presence of TMSCF3, and by implication, CF_3 anion, on the conversion of the ketal intermediates (**3**^{TMS}) into the difluorinated coumaranones (**4**) we added tested the effect of addition of CO_2, which has been shown to react rapidly with CF_3 anion to generate trifluoroacetate.[S1] For the reaction of **2a**^{H} we removed the NMR tube from the spectrometer once the maximum concentration of intermediate **3a**^{TMS} had been reached, and 20 mL CO_2 gas gently added via a syringe connected to a long narrow needle, so that is bubbled through the reaction mixture over a period of approximately half a minute. Both reaction profiles (Figure S23) show that after CF_3 anion is trapped with CO_2, the ketones (**4**) continue to form. In addition to the ^{29}Si NMR reaction monitoring, this supports the conclusion that ethoxide anion is the chain carrier for the formation of ketone **4** + TMSOEt from corresponding ketal intermediate (**3**^{TMS}).

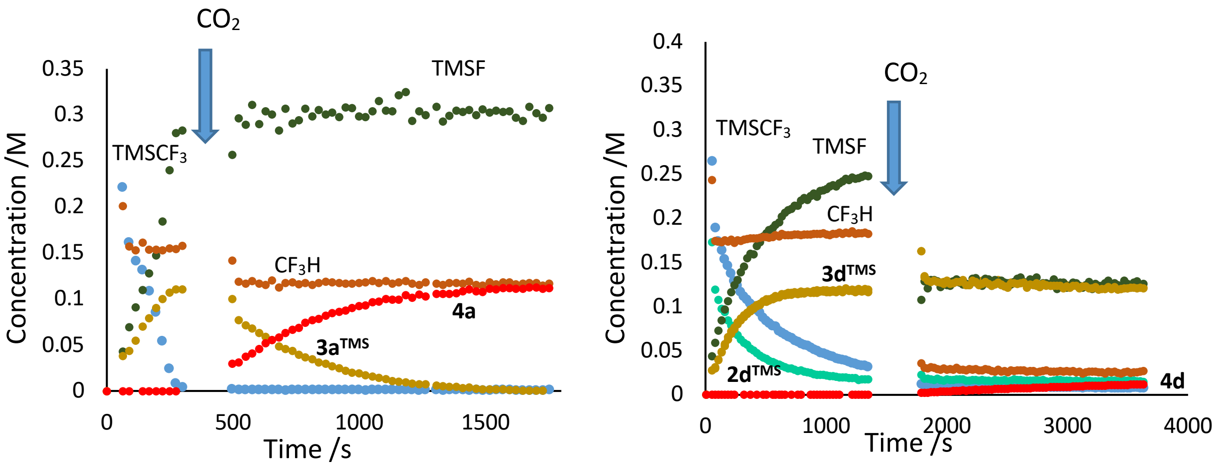

Figure S23. a) Ethyl salicylate (**2a**^{H}), $TMSCF_3$ and TBAT under standard conditions monitored by ^{19}F NMR (376 Hz). CO_2 was briefly bubbled through the reaction mixture at the point indicated. The NMR tube was then shaken, and reinserted into the NMR spectrometer for further monitoring. The absence of data in the temporal concentration plot between 298 and 495 seconds is a result of the addition of CO_2 to the reaction mixture between multi_zgvd commands, as indicated by the vertical arrow. B) Ethyl 5-fluoro-2-hydroxybenzoate (**2d**^{H}), $TMSCF_3$ and TBAT under standard conditions monitored by ^{19}F NMR (376 Hz). CO_2 was briefly bubbled through the reaction mixture at the point indicated. The NMR tube was then shaken, and reinserted into the NMR spectrometer for further monitoring. The CO_2 sparging also partially displaces volatile species such as TMSF and CF_3H. The absence of data in the temporal concentration plot between 1344 and 1785 seconds is a result of the addition of CO_2 to the reaction mixture between multi_zgvd commands, as indicated by the vertical arrow.

S7.2. KOEt addition

To further support the conclusion that ethoxide anion initiates and carries the anionic chain reaction that converts the ketal (**3**^{TMS}) into the difluorinated coumaranone (**4**) KOEt was added to a reaction in which **3d**^{TMS} was in stage IV.

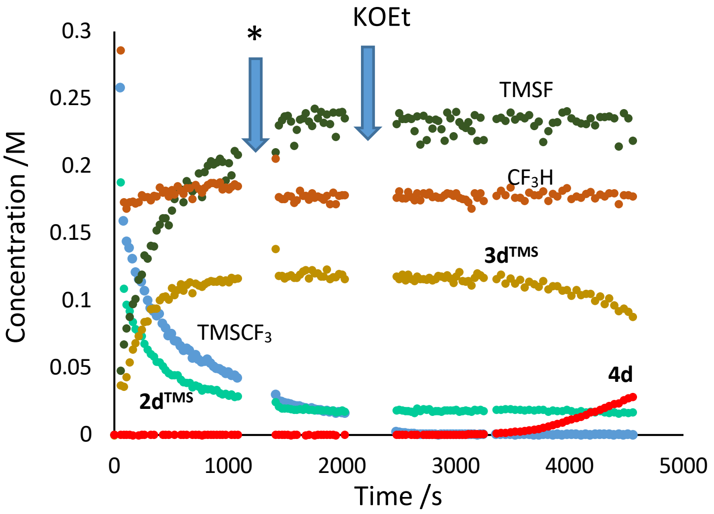

Figure S24. a) Reaction of ethyl 5-fluoro-2-hydroxybenzoate (**2d**^{H}), with $TMSCF_3$ and TBAT under standard conditions monitored by ^{19}F NMR (376 Hz). KOEt (6 mg) was added to the reaction mixture at the point indicated. The NMR tube is then shaken vigorously for 30 seconds, and reinserted into the NMR spectrometer for further monitoring, which showed breakdown of the ketal intermediate to form the coumaranone **4d**. * = The absence of data, or reduced data density in the temporal concentration plot between 1082 and 1417 seconds is a result of unscheduled delays in spectral acquisition during NMR monitoring due to e.g. issues with automated tuning / matching when using the multi_zgvd command. The absence of data in the temporal concentration plot between 2024 and 2475 seconds is a result of the addition of KOEt to the reaction mixture between multi_zgvd commands, as indicated by the vertical arrow.

.

S7.3. H_2O addition

The reaction of ethyl 5-fluoro-2-hydroxybenzoate, **2d^H^** (0.2 M), $TMSCF_3$ (0.5 M) and TBAT (0.015 M) was conducted as usual to accumulate ketal intermediate **3^{TMS}** in stage IV. On addition of water (approximately 34 mg – added via Pasteur pipette), **$3d^{TMS}$** rapidly converts to ketone **4d**. Salicylate **$2d^H$** is generated by hydrolysis of **$2d^{TMS}$**. A sample of the reaction mixture was taken before addition of water and analyzed by TLC, with a reference sample of previously isolated **4d**. Ketal **$3d^{TMS}$** is converted to **4d** during the preparation and/or elution of the TLC sample. Comparison of ^1H/^{29}Si/^{19}F NMR spectra taken before and after the addition of water, shows that the silyl group in **$3d^{TMS}$** is converted to TMSOH on the addition of water.

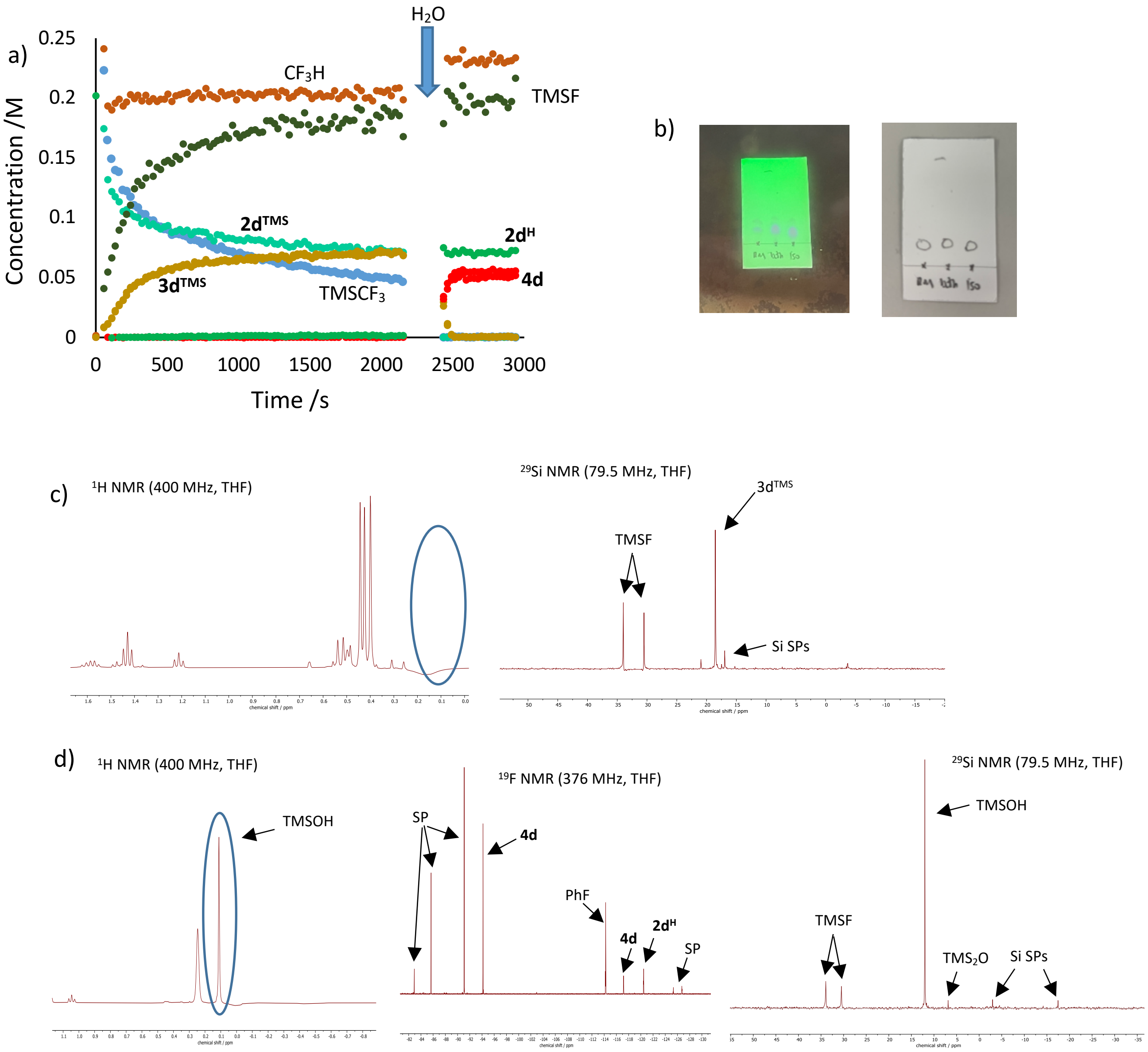

Figure S25. a) Ethyl 5-fluoro-2-hydroxybenzoate (**$2d^H$**) (0.2 M), $TMSCF_3$ (0.5 M) and TBAT (0.015 M) under standard conditions monitored by ^{19}F NMR (376 Hz). The absence of data in the temporal concentration plot between 2149 and 2434 seconds is a result of the addition of H_2O to the reaction mixture between multi_zgvd commands, as indicated by the vertical arrow. b) TLC taken before the addition of H_2O, showing hydrolysis of the ketal **$3d^{TMS}$** to ketone **4d** on the TLC plate c) ^1H and ^{29}Si spectra taken before the addition of H_2O. d) ^1H, ^{19}F and ^{29}Si NMR spectra taken after the addition of H_2O, showing formation of TMSOH, ketone **4d** and traces of TMSOTMS.

S7.4. ^{29}Si monitoring

To further probe the system in Stage IV, the reactions of **2a^{H}** and **2e^{H}** were monitored by in situ ^{29}Si INEPT NMR.

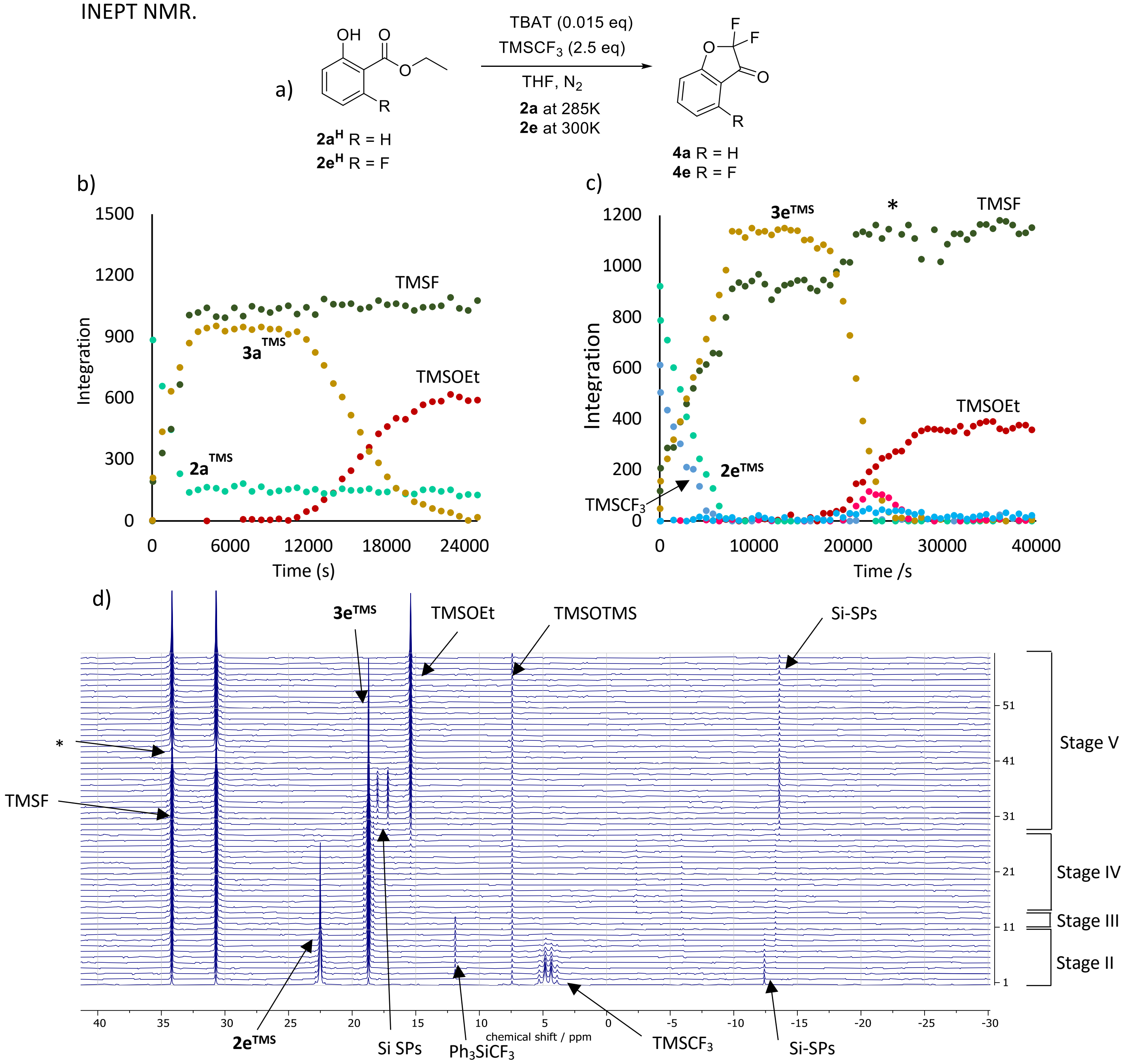

Figure S26. a) Conditions used for ^{29}Si semi-quantitative monitoring **2a^{H}** or **2e^{H}** (0.2 M), $TMSCF_3$ (0.5 M), TBAT (0.015 M) b) ^{29}Si NMR (79.5 Hz) semi-quantitative reaction monitoring of the formation of ketal **3e^{TMS}** and TMSF and breakdown of ketal **3e^{TMS}** to ketone **4e** and TMSOEt. c) Analogous ^{29}Si NMR (79.5 Hz) semi-quantitative reaction monitoring of ketal **3e^{TMS}**. d) Stacked ^{29}Si NMR (79.5 Hz) NMR spectra of the reaction of **2e^{H}** showing breakdown of ketal **3e^{TMS}** and formation of TMSOEt at -15.3 ppm. *= Anion-induced dynamic line broadening in TMSF. A number of Si-containing side products (SPs) were observed but not identified. The species at -12 ppm were not identified however may be aryl silicon species. The assignment of EtOTMS was based on a reference sample prepared by TBAT initiated reaction of excess EtOH with $TMSCF_3$ in THF: ^{29}Si NMR (79.5 Hz) 15.3 ppm, singlet.

S8. Additional Observations

S8.1. Kondo Silylation

Analysis of the reaction of ethyl 4-fluoro-2-hydroxybenzoate (**2c**^{H}), shows that, for this substrate, a significant side reaction is silylation[S2]of the aryl ring of **3c**^{TMS} to generate **9c** + CF_3H

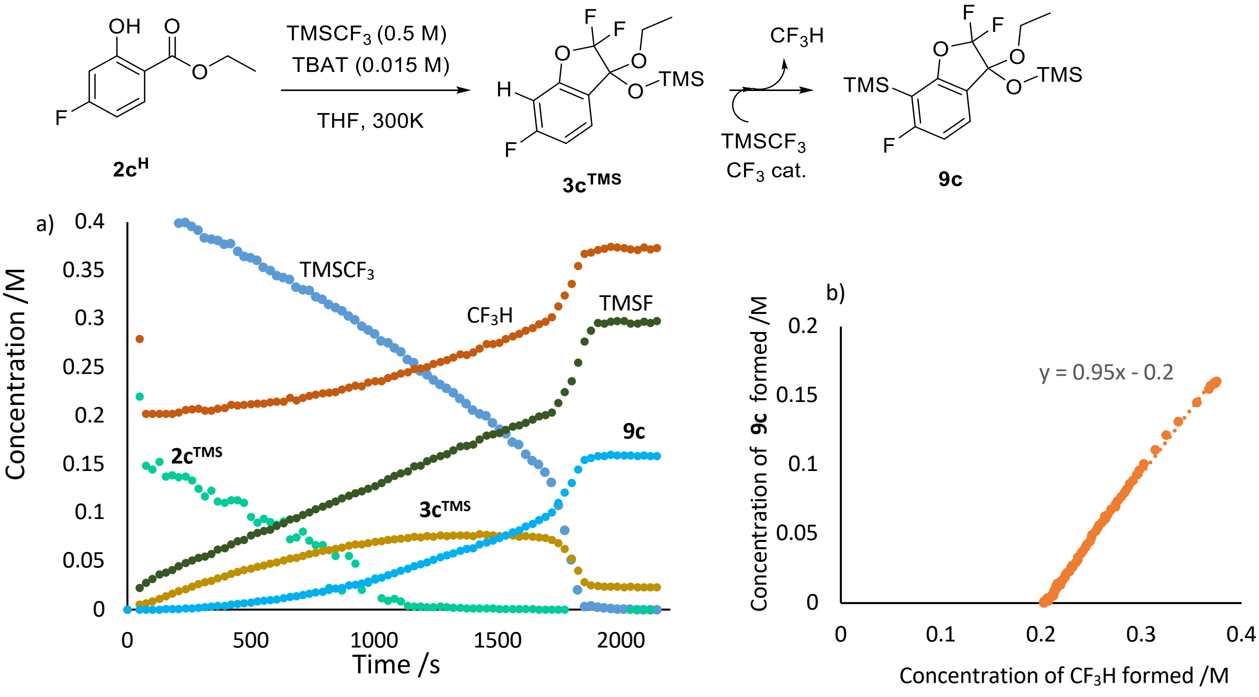

Figure S27. a) Temporal concentration plot of the reaction **2c**^{H} (0.2 M), $TMSCF_3$ (0.65 M) and TBAT (0.015 M) monitored by ^{19}F NMR (376 Hz), showing conversion of **3c**^{TMS} to **9c**. b) Concentration of CF_3H formed vs concentration of silylation product formed **9c**.

Kondo silylation requires the adjacent C-H bond to be sufficiently acidic to allow for deprotonation by the CF_3 anioni(oid). During transition from stage II to stage III and the accompanying acceleration in TMSF generation the rising concentration of CF_3 anion results in accelerated conversion of**3c**^{TMS} to **9c**.

S8.2. D_2O-quenching of Kondo Silylation product 9c.

Attempts to isolate the aryl-silylated ketone corresponding to ketal hydrolysis in **9c**, resulted in isolation of **4c**, leading to the conclusion that solvolytic aryldesilyation is occurs readily. The reaction of **2c**^{H} (0.2 M), $TMSCF_3$ (0.5 M) and TBAT (0.015 M) was monitored ^{19}F NMR (376 Hz) and at the stage that the silylated product **9c** was present in significant concentration, D_2O (~0.1 M) was added. Analysis of the ^{19}F NMR spectrum (THF/D_2O) showed that **9c** was converted to 3-[^{2}H]-**4c**, with residual **3c**^{TMS} being converted to **4c**, leading to approximately approximately 57 % overall aryl deuteration in **4c**, Figure S28.

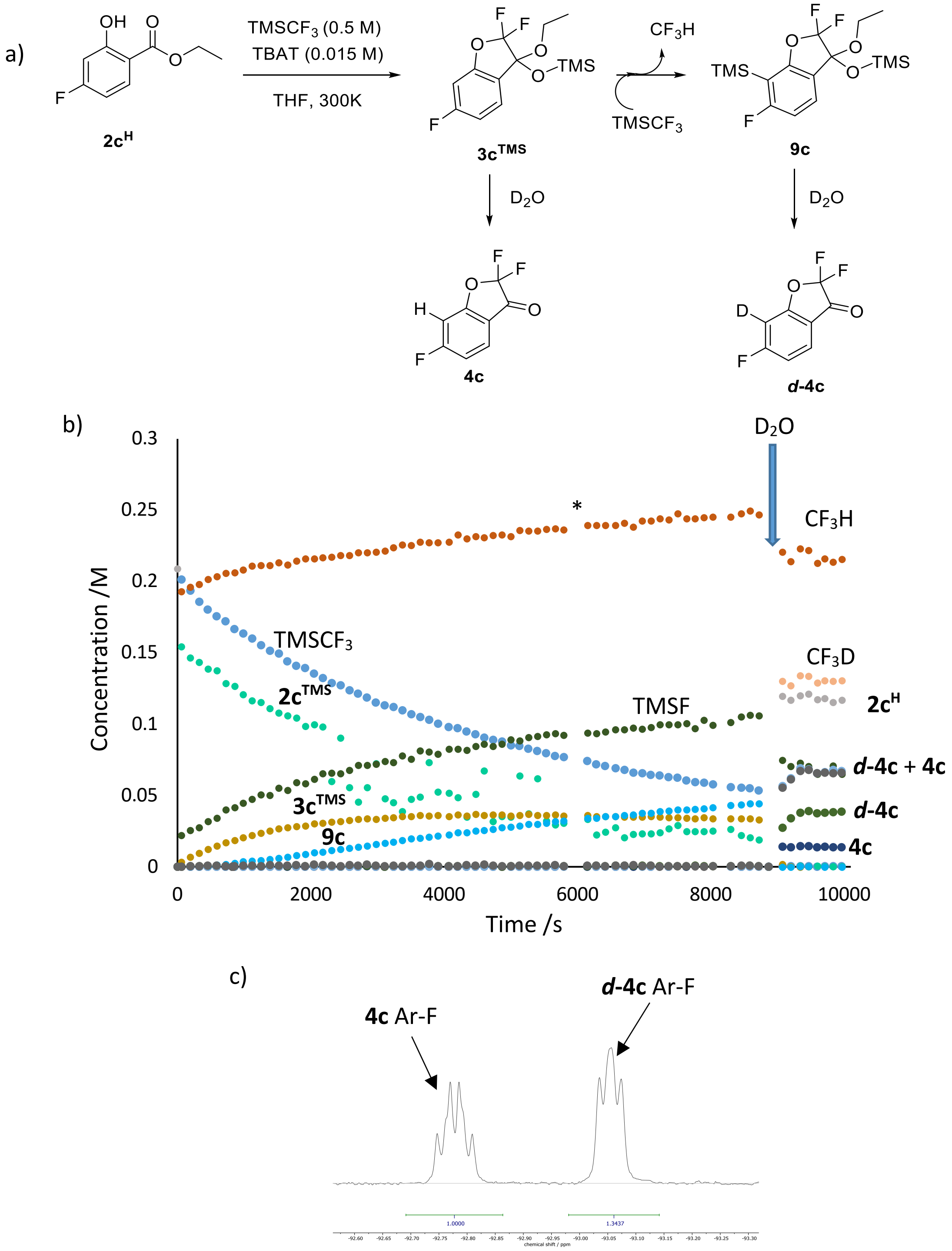

Figure 28. a) Intermediates and products detected in the reaction of **2c^H** (0.2 M), $TMSCF_3$ (0.5 M), TBAT (0.015 M) monitored by ^{19}F NMR (376 Hz). B) temporal concentration plot of the reaction. c) In situ ^{19}F NMR (376 Hz) analysis of the deuteration in **4c** after addition of D_2O. The ^{2}H induces an approximately 0.3 ppm downfield isotope shift in the ^{19}F NMR signal of the *o*-F ***d*-4c** (-93.0 ppm) and protonated **4c** (-92.7 ppm), the integrations indicating approximately 57% D incorporation.*= The absence of data, or reduced data density in the temporal concentration plot between 5794 and 6148 seconds is a result of unscheduled delays in spectral acquisition during NMR monitoring due to e.g. issues with automated tuning / matching when using the multi_zgvd command. The absence of data in the temporal concentration plot between 8719 and 9074 seconds is a result of the addition of D_2O to the reaction mixture between multi_zgvd commands, as indicated by the vertical arrow.

The experiment was repeated after decreasing the initial concentration of **2c**^{H} to 0.1 M to induce greater CF_3 anion concentration, and thus more rapid stage II, III silylation.

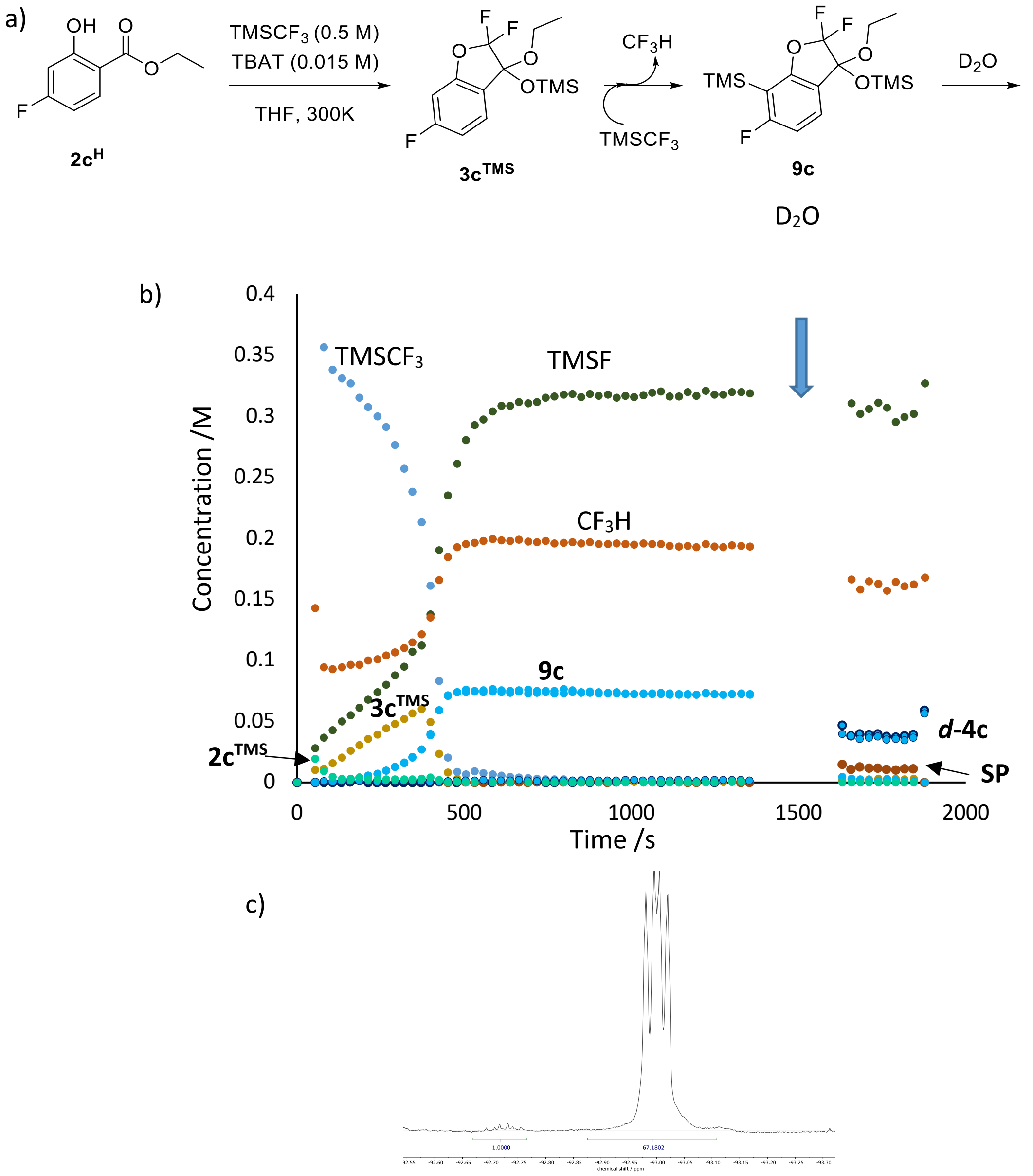

Figure 29. a) Reaction scheme with observed intermediates and products observed in the reaction of ethyl 4-fluoro-2-hydroxybenzoate (0.1 M), $TMSCF_3$ (0.5 M), TBAT (0.015 M). b) Kinetic plot of ethyl 4-fluorohydroxybenzoate (**2c**^{H}, 0.2 M), $TMSCF_3$ (0.5 M), TBAT (0.015 M) monitored by ^{19}F NMR (376 Hz). The absence of data in the temporal concentration plot between 1353 and 1657 seconds is a result of the addition of D_2O to the reaction mixture between multi_zgvd commands, as indicated by the vertical arrow. SP = side product. c) No observed protonated product **4c** forms (92.7 ppm), only ***d*-4c** (-93.0 ppm). Using their respective integrals, over 99% of **3c**^{TMS} was converted to **9c**, and the latter converted to 3-[^{2}H]-**4c** / **4c** (> 98% D) on addition of D_2O. A side product was also formed on addition of D_2O, evident as a singlet in the ^{19}F NMR (376 Hz) spectrum; indicative of a non-aryl fluorine atom.

S8.3. Experiments comparing ethyl and methyl salicylates

To further probe the system, standard reactions were carried out with 5-fluoro substrate, **2d^H^** and the analogous methyl ester (**S3d^H^**), Figures 30, and 31. Using concentration-concentration plots, it is evident that the reaction becomes less efficient when using the methyl ester with the ratio of CF_3 addition to ketal formation increases.

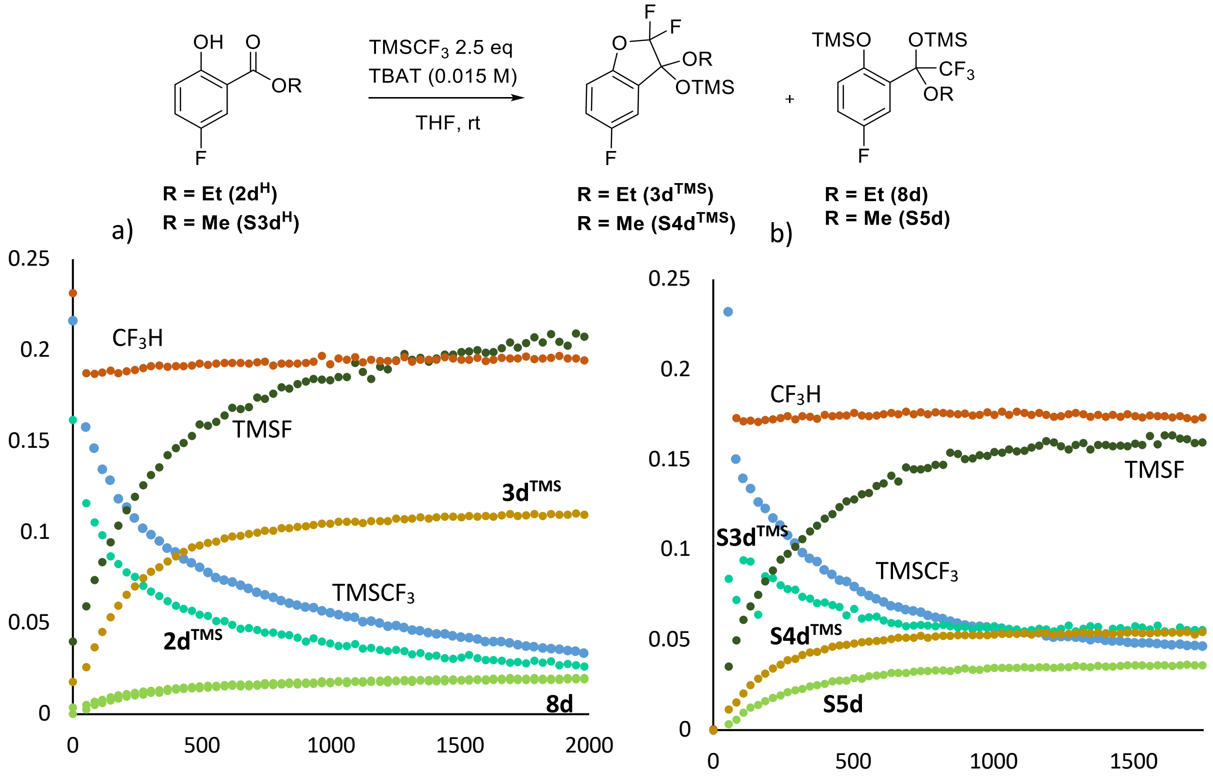

Figure 30. a) Kinetic profile for reaction of ethyl 5-fluoro-2-hydroxybenzoate (**2d^H^**, 0.2 M), $TMSCF_3$ (0.5 M) and TBAT (0.015 M), b) Kinetic profile for methyl 5-fluoro-2-hydroxybenzoate (**S3d^H^**, 0.2 M), $TMSCF_3$ (0.5 M) and TBAT (0.015 M). Monitored *in situ* by ^{19}F NMR (376 Hz) at 300 K.

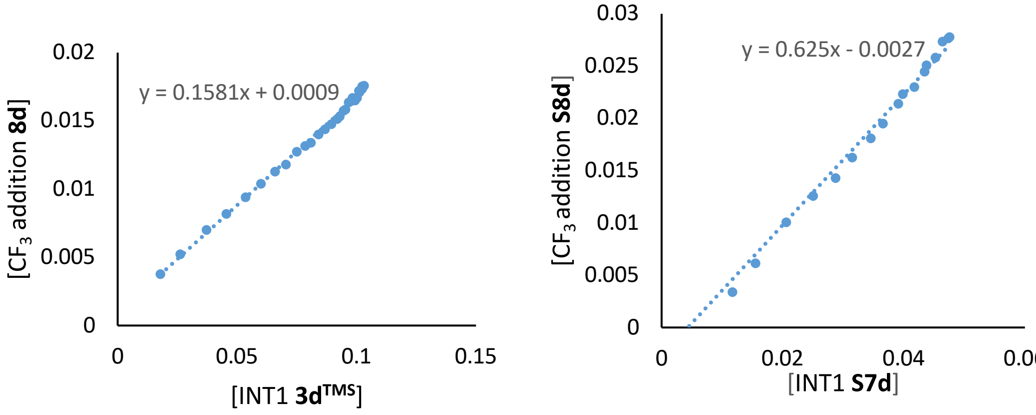

Figure 31. a) concentration-concentration plot for ethyl 5-fluoro-2-hydroxybenzoate (**2d^H^**, 0.2 M), $TMSCF_3$ (0.5 M) and TBAT (0.015 M) reaction monitored by ^{19}F NMR (376 Hz), where CF_3 addition is plotted on the y-axis and formation of the ketal intermediate (**3d^TMS^**) is plotted on the x-axis. b) concentration-concentration plot for methyl 5-fluoro-2-hydroxybenzoate (**2d^H^**, 0.2 M), $TMSCF_3$ (0.5 M) and TBAT (0.015 M) reaction, where CF_3 addition is plotted on the y-axis and formation of the ketal intermediate is plotted on the x-axis.

S9. Relative Rates of Salicylates in Competition

S9.1. Competition reaction, ethyl salicylate ($2a^H$) and ethyl 5-fluoro-2-hydroxybenzoate ($2d^H$)

Competition between ethyl salicylate (**$2a^H$**) and ethyl 5-fluoro-2-hydroxybenzoate (**$2d^H$**) showed **$2d^H$** reacts faster, however it also forms a more stable ketal (**$3d^{TMS}$**) with **$3a^{TMS}$** converting faster to ketone **4a** after an extended period of quasi stasis in stage IV

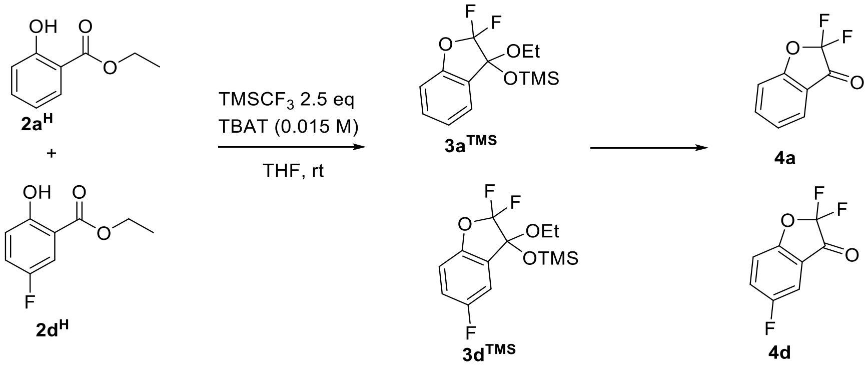

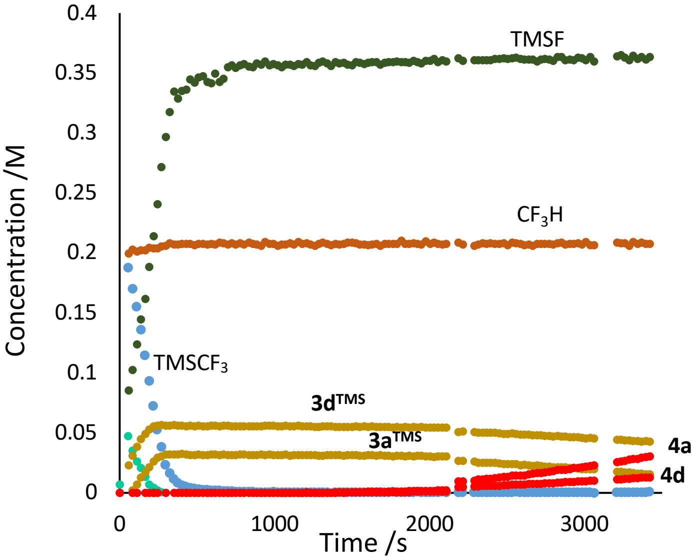

Figure S32. Kinetic profile for the competition of ethyl 5-fluoro-2-hydroxybenzoate (**$2d^H$**, 0.1 M), with ethyl salicylate (**$2a^H$**, 0.1 M), on reaction with $TMSCF_3$ (0.5 M) and TBAT (0.015 M), monitored by ^{19}F NMR (376 Hz). The absence of data, or reduced data density in the temporal concentration plot between 2106 and 2286 seconds is a result of unscheduled delays in spectral acquisition during NMR monitoring due to e.g. issues with automated tuning / matching when using the multi_zgvd command. The absence of data in the temporal concentration plot between 3061 and 3206 seconds is a result of the time taken to initiate another multi_zgvd command, after the prior one has ended, to capture further evolution of the process.

S9.2. Competition between ethyl 4-fluoro-2-hydroxybenzoate ($2c^H$) and ethyl 5-fluoro-2-hydroxybenzoate ($2d^H$)

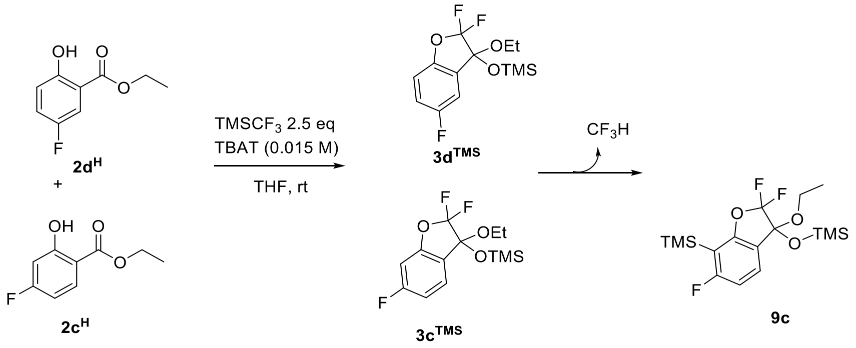

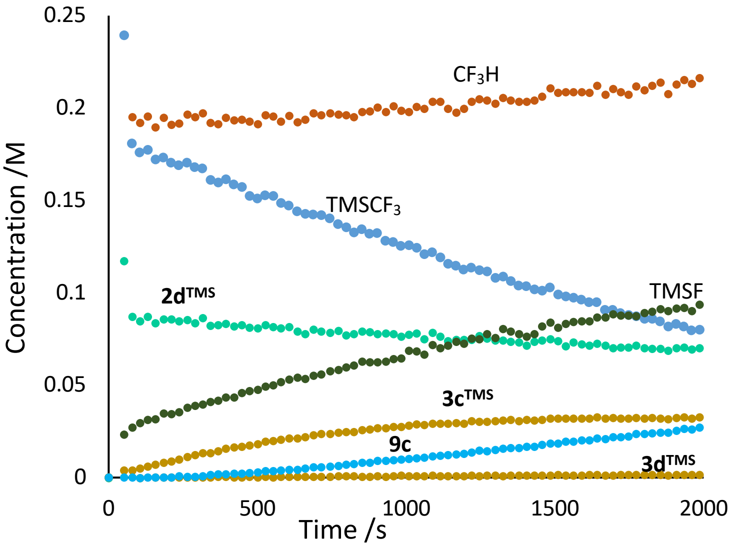

Figure S33: Kinetic profile for competition between ethyl 5-fluoro-2-hydroxybenzoate (**$2d^H$**, 0.1 M), ethyl 4-fluoro-2-hydroxybenzoate (**$2c^H$**, 0.1 M), on reaction with $TMSCF_3$ (0.5 M) and TBAT (0.015 M) monitored by ^{19}F NMR (376 Hz) at 300 K. Formation of both intermediates (**$3c^{TMS}$** and **$3d^{TMS}$**) is observed, with extensive silylation of **$3c^{TMS}$** to generate **9c**.

S9.3. Competition between salicylate silyl ethers, 2a^{TMS} and 2d^{TMS}.

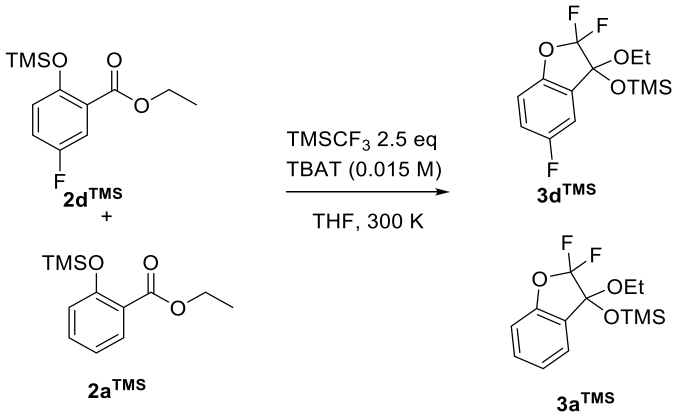

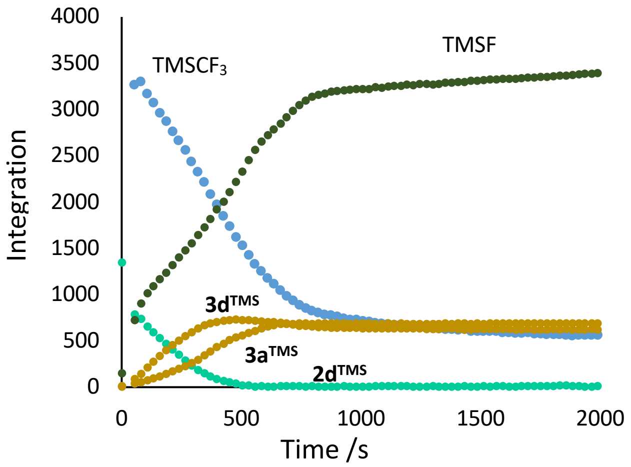

Figure S34. Kinetic profile for competition between **2d^{TMS}** (0.1 M), and **2a^{TMS}** (0.1 M), on reaction with TMSCF$_3$ (0.5 M) and TBAT (0.015 M) monitored by ^{19}F NMR (376 Hz). Ketal intermediate (**3d^{TMS}**) is generated faster than **3a^{TMS}**, as also observed in Figure S28.

S9.4. Competition between salicylates, 2b^H and 2e^H.

Competition experiment between ethyl 3-fluoro-2-hydroxybenzoate, **2b^H** and ethyl 6-fluoro-2-hydroxybenzoate **2e^H**, shows that 3-fluoro substrate **2b^H** reacts faster in competition whereas the 6-fluoro substrate **2e^H** reacts faster in isolation.

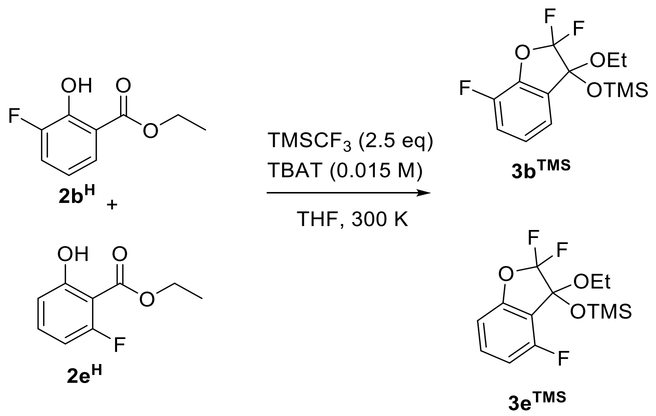

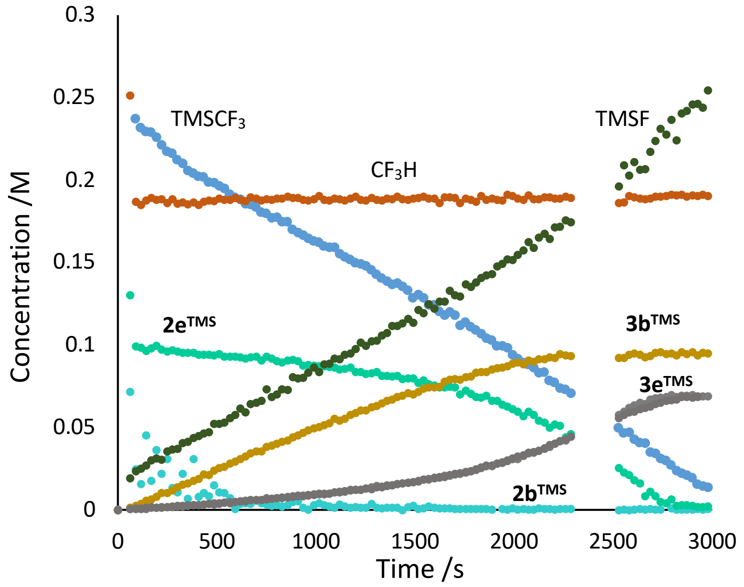

Figure S35. Kinetic profile for competition between **2b^H** (0.1 M), and **2e^H** (0.1 M), on reaction with TMSCF$_3$ (0.5 M) and TBAT (0.015 M) monitored by ^{19}F NMR (376 Hz). Ketal intermediate (**3b^{TMS}**) is generated faster than **3e^{TMS}**. The absence of data in the temporal concentration plot between 2285 and 2526 seconds is a result of the time taken to initiate another multi_zgvd command, after the prior one has ended, to capture further evolution of the process.

S9.5. Addition of $TMSCF_3$ During Stage IV

Once the accelerating TMSF generation under standard conditions (stage III) had ceased, i.e., reached stage IV with near-complete consumption of $TMSCF_3$, we tested whether the system contains 'active anions' during stage IV by addition of further $TMSCF_3$ (1 equiv.). Transient autoaccelerating TMSF generation is observed, thus the system re-enters stage III then returns to stage IV.

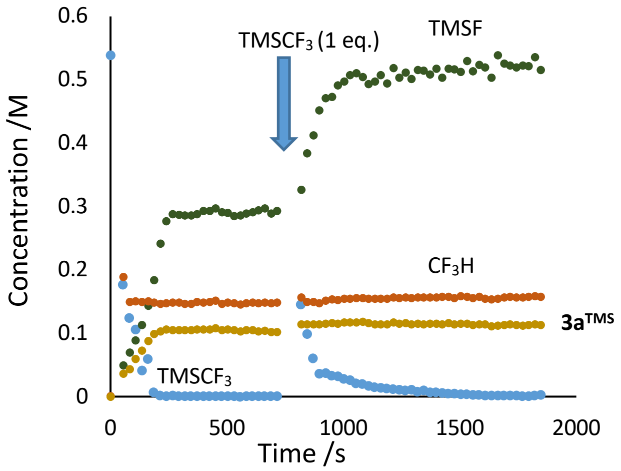

Figure S36. Kinetic profile of ethyl salicylate (**2a**^{H}, 0.2 M), $TMSCF_3$ (0.5 M) and TBAT (0.015 M) monitored by ^{19}F NMR (376 Hz). $TMSCF_3$ (0.25 M) was added once autoacceleration had finished. The absence of data in the temporal concentration plot between 714 and 817 seconds is a result of the addition of $TMSCF_3$ (1 eq.) to the reaction mixture between multi_zgvd commands, as indicated by the vertical arrow.

S9.6. Addition of $TESCF_3$ During Stage II/III of Reaction Initiated with$TMSCF_3$

Conversely, addition of $TESCF_3$ during stages II/III slowed the formation of ketals **3a**^{TMS} and **S2a**, and converted some TMSF to TESF, as well as generating TESF.

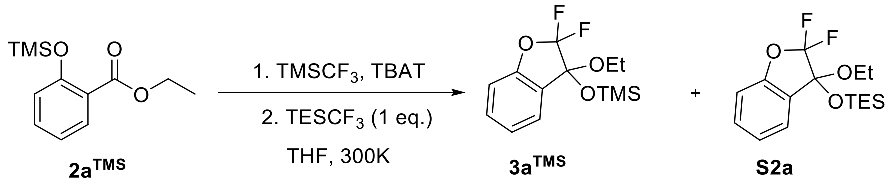

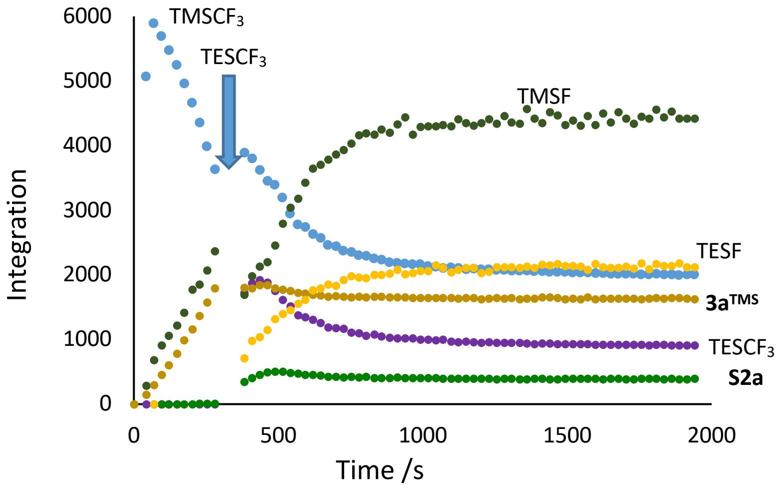

Figure S37. Kinetic profile of reaction of **2a**^{TMS} (0.2 M), with $TMSCF_3$ (0.5 M) and TBAT (0.015 M) monitored by ^{19}F NMR (376 Hz). $TESCF_3$ (0.25 M) was added during the reaction at the indicated point. The absence of data in the temporal concentration plot between 280 and 409 seconds is a result of the addition of $TESCF_3$ (1 eq.) to the reaction mixture between multi_zgvd commands, as indicated by the vertical arrow.

S10. Kinetic Simulations of the Reported Mechanism.[S4]

Kinetic models were fitted to experimental data using a standard numerical methods approach[S5] implemented through Excel with a proprietary scale-up systems add-in.

Various limiting regimes of the mechanism originally proposed[S4] were explored to confirm that the mechanism (**2^{H}** + $TMSCF_3$ + F^- --> **2^{TMS}** + CF_3H + F^- --> **3^{TMS}** + F^- --> **4** + TMSF) requires stoichiometric TBAT, cannot account for the accelerating TMSF generation in stage III, and does not proceed to give the reported >85% conversion of **2^{H}** to **4**. The steps in the mechanism used for the simulation were taken directly from the original work (scheme S1) but also includes generation of TMSF on initiation by TBAT.[S4] Four scenarios (S10A, S10B, S10C, and S10D) were considered in the simulated plots (Tables S1 to S4). Each began with **2a^{H}** (0.4 M), $TMSCF_3$ (1 M) and TBAT (0.03 M), unless noted.

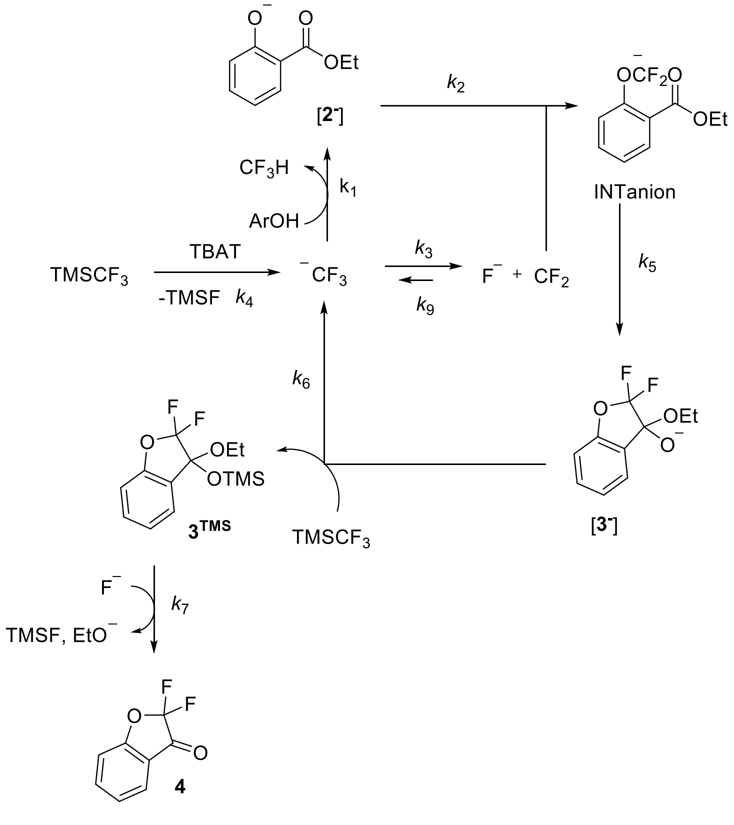

Scheme S1. The previously proposed mechanism[S4] for (**2^{H}** --> **2^{TMS}** --> **3^{TMS}** --> **4**) used for the four sets of kinetic simulations discussed in Section S10.

In the first set of simulations, S10A, the initial rate of CF_2 generation by α-elimination was set to equal the initial rate of deprotonation of the phenol. In this regime, 3-coumaranone **4** reaches a maximum concentration of 0.10 M, and a large proportion of $TMSCF_3$ (0.8 M) and **2^{H}** (0.29 M) remain. Because there is no CF_3 anion regeneration (in the model) when CF_2 is produced the system is progressively depleted in active anion and stalls. In this system the maximum yield is approximately 25%. The right hand plot details the formation of phenoxide and **3^{TMS}**, the intermediate ketal generated in the reaction, and subsequent breakdown of the ketal to form the product.

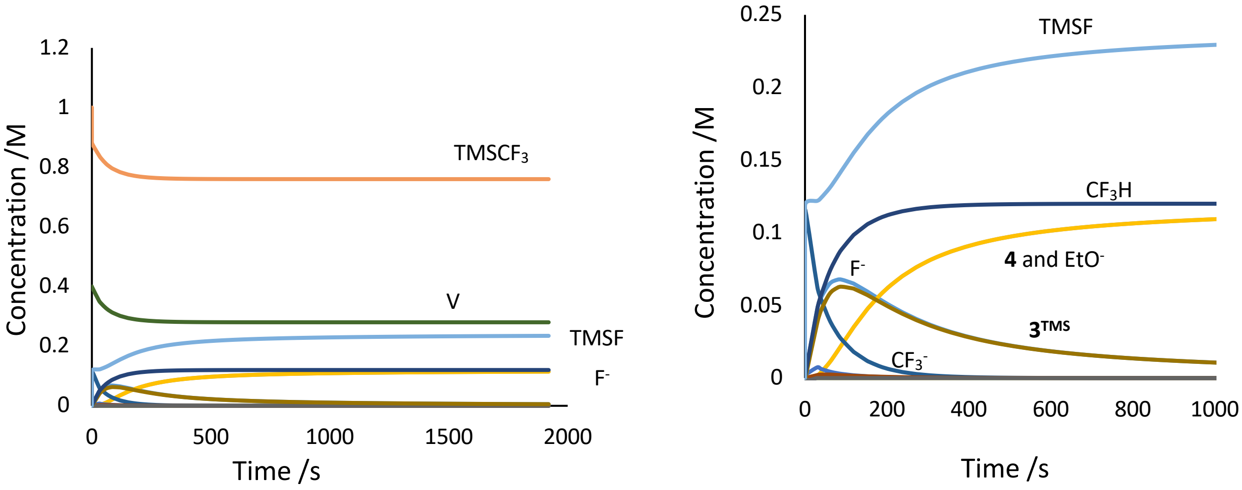

Figure S38. Simulated kinetic data (S10A) of the previously proposed mechanism,[S4] with the initial rate of carbene generation and deprotonation are set to be equal. A large proportion of **2**^{H} remains unreacted. Inset: key intermediates forming and decaying at low concentrations.

Table S1. The rate coefficients and steps used in simulation S10A. Rates were adjusted so that the overall process evolved on the approximate timescale previously reported.[S4]

rate coefficient	value	units	Step
k_1	0.05	L/mol.s	**2**^{H} + CF_3anion > [**2**$^-$] + CF_3H
k_2	1e^8	L/mol.s	[**2**$^-$] + CF_2 > INTanion
k_3	0.02	1/s	CF_3anion > F^- + CF_2
k_4	200	L/mol.s	TBAT + $TMSCF_3$ > CF_3anion + TMSF
k_5	200	1/s	INTanion > [**3**$^-$]
k_6	0.2	L/mol.s	[**3**$^-$] + $TMSCF_3$ > **3**^{TMS} + CF_3anion
k_7	0.1	L/mol.s	**3**^{TMS} + F^- > TMSF + **4** + EtO
k_8	1	L/mol.s	F^- + CF_2 > CF_3anion

In simulation S10B the rate of deprotonation forming the phenoxide is set to be 10-fold faster than the rate of CF_2 generation, Table S2. The simulation shows the system does not regenerate CF_3 anion and quickly stalls as the rate of carbene generation falls, with residual [**2**$^-$] present, and only a low concentration of the ketal intermediate **3**^{TMS} being formed. Although 0.1 M of the **2**^{H} is deprotonated, only 0.01 M of the final ketone **4** forms as a result of the reduced rate of carbene generation. The maximum yield of this reaction is ca. 3.5%.

Consequently, the mechanism still cannot be correct due to the reduced yield on comparison with experimental data, and the simulated reaction mechanism is not catalytic as the authors proposed.

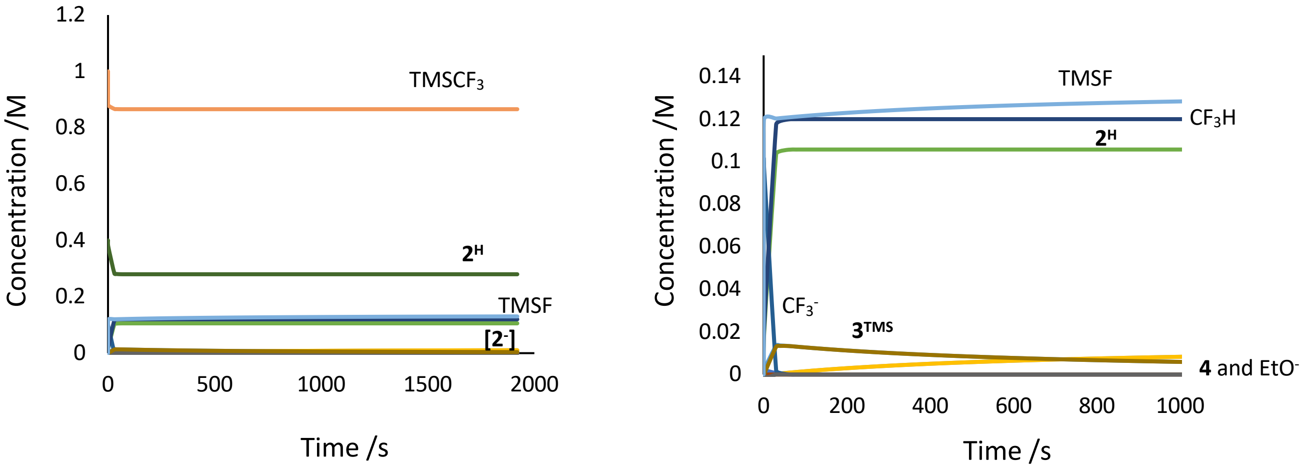

Figure S39. Simulated kinetic data (S10B) of the previously proposed mechanism,[S4] with the initial rate of deprotonation set 10-fold faster than the rate of carbene generation in. As a result less product **4** forms than Simulation S10A, Figure S38, and again the reaction stalls Inset: key intermediates forming and decaying at low concentrations.

Table S2. The rate coefficients and steps used in simulation S10B. Rates were adjusted so that the overall process evolved on the approximate timescale previously reported.[S4]

rate coefficient	value	units	Step
k_1	0.5	L/mol.s	$\mathbf{2}^{\mathbf{H}}$ + CF_3anion > [**2**$^-$] + CF_3H
k_2	1e^8	L/mol.s	[**2**$^-$] + CF_2 > INTanion
k_3	0.02	1/s	CF_3anion > F^- + CF_2
k_4	200	L/mol.s	TBAT + $TMSCF_3$ > CF_3anion + TMSF
k_5	200	1/s	INTanion > [**3**$^-$]
k_6	0.2	L/mol.s	[**3**$^-$] + $TMSCF_3$ > $\mathbf{3}^{\mathbf{TMS}}$ + CF_3anion
k_7	0.1	L/mol.s	$\mathbf{3}^{\mathbf{TMS}}$ + F^- > TMSF + **4** + EtO
k_8	1	L/mol.s	F^- + CF_2 > CF_3anion

In simulation S10C the rate of deprotonation forming the phenoxide is set to be 10-fold slower than the rate of CF_2 generation, Table S3. This results in accumulation of fluoride anion and CF_2. The CF_2 is unable to react further as there is insufficient phenoxide [**2**$^-$] generated. The total concentration of $\mathbf{2}^{\mathbf{H}}$ remaining in the reaction is 0.38 M and the maximum yield of **4** is 2.9%.

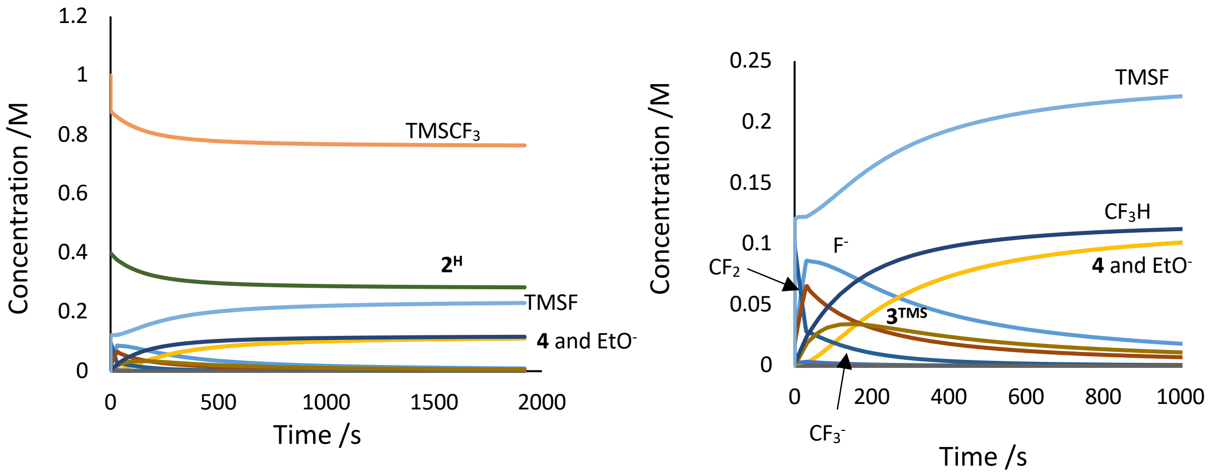

Figure S40. Simulated kinetic data (S10C) of the previously proposed mechanism,[S4] with the initial rate of deprotonation set 10-fold slower than the rate of carbene generation, Table S3. Inset: key intermediates forming and decaying at low concentrations.

Table S3. The rate coefficients and steps used in simulation S10C. Rates were adjusted so that the overall process evolved on the approximate timescale previously reported.[S4]

rate coefficient	value	units	Step
k_1	0.05	L/mol.s	$\mathbf{2}^{\mathrm{H}}$ + CF_3anion > [$\mathbf{2}^-$] + CF_3H
k_2	$1e^8$	L/mol.s	[$\mathbf{2}^-$] + CF_2 > INTanion
k_3	0.2	1/s	CF_3anion > F^- + CF_2
k_4	200	L/mol.s	TBAT + $TMSCF_3$ > CF_3anion + TMSF
k_5	200	1/s	INTanion > [$\mathbf{3}^-$]
k_6	0.2	L/mol.s	[$\mathbf{3}^-$] + $TMSCF_3$ > $\mathbf{3}^{\mathrm{TMS}}$ + CF_3anion
k_7	0.1	L/mol.s	$\mathbf{3}^{\mathrm{TMS}}$ + F^- > TMSF + **4** + EtO
k_8	1	L/mol.s	F^- + CF_2 > CF_3anion

Finally, in simulation S10D the initial TBAT concentration was raised to 0.4 M. This is four times higher than that used in the reported study.[S4] In this simulation (S10D), a high yield of **4** is observed (ca. 95%). However the process is now stoichiometric in TBAT.

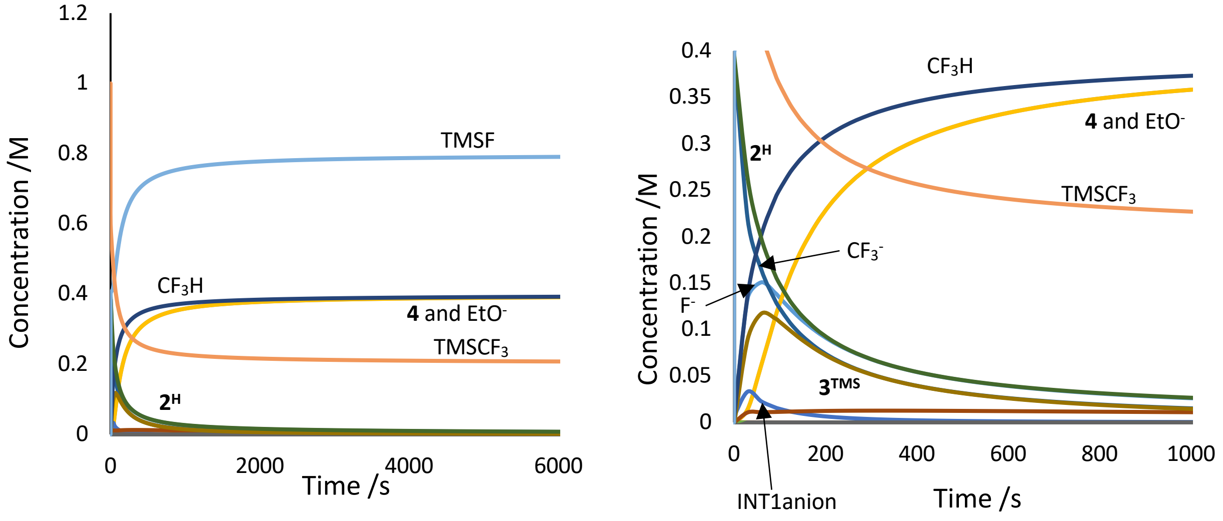

Figure S41. Simulated kinetic data of the reaction mechanism proposed by Zhao. This simulation increases the concentration of TBAT. Inset: key intermediates forming and decaying.

Table S4. The rate coefficients and steps used in simulation S10D. Rates were adjusted so that the overall process evolved on the approximate timescale previously reported.[S4]

rate coefficient	value	units	Step
k_1	0.05	L/mol.s	**2**^{H} + CF_3anion > [**2**$^-$] + CF_3H
k_2	1e^8	L/mol.s	[**2**$^-$] + CF_2 > INTanion
k_3	0.02	1/s	CF_3anion > F^- + CF_2
k_4	200	L/mol.s	TBAT + $TMSCF_3$ > CF_3anion + TMSF
k_5	200	1/s	INTanion > [**3**$^-$]
k_6	0.2	L/mol.s	[**3**$^-$] + $TMSCF_3$ > **3**^{TMS} + CF_3anion
k_7	0.1	L/mol.s	**3**^{TMS} + F^- > TMSF + **4** + EtO
k_8	1	L/mol.s	F^- + CF_2 > CF_3anion

In the first three simulations (S10A-C), *substoichiometric* TBAT is unable to initiate a sufficiently productive anionic chain reaction to completely convert salicylate into ketal or ketone. Accumulation of fluoride and other anions result in no CF_3 anion regeneration and thus chain termination. Indeed, the active anion depletes by half in each revolution of the process. The simulations also result in TMSF generation in parallel with ketone **4**. However in the reported ^{19}F NMR study,[S4] no TMSF is generated in stage V. Moreover, the loss of fluoride from the CF_3 anion to generate CF_2 has previously been shown not to be *via* a direct α-elimination but *via* a process involving $TMSCF_3$.[S3]

S11. Kinetic Analyses of the Expanded Mechanism

S11.1. Kinetic Simulations

The discussion below explores simulation of the kinetics of the mechanism proposed in the main manuscript (Figure 5) for the reaction of **2e**^{H}. This model is the conclusion of a large array of models explored, with processes eliminated by testing, including equilibria between the ketal intermediate **3e**^{TMS} and anion [**2e**$^{-}$], and homoconjugation [**2**$\cdots$**2e**^{H}]$^{-}$. The kinetic data that has been modelled was obtained by in situ ^{19}F NMR spectroscopic analysis as detailed in section S1. The initial concentrations used in the model were determined from the ^{19}F NMR spectra at the points before and after initiation by TBAT, except for $TMSCF_3$ which due to extensive line broadening in stages II,III was estimated from the endpoint species; $[TMSCF_3]_0 = [TMSF]_{end} + [CF_3H]_{end}$, and the phenol **2e**^{H} which was estimated from the average of the **2e**^{TMS} and CF_3H formed in stage I: [**2e**^{H}] = (([**2e**^{TMS}] + $[CF_3H]$)/2). The initial concentration of TBAT initiator was based on the concentration and volume of the stock solution added. The model presented is the simplest system we were able to develop for the reasonably satisfactory simulation of the kinetics of the reaction starting with 6-fluoro-2-hydroxybenzoate **2e**^{H}, or its TMS ether **2e**^{TMS}, into the ketal intermediate **3e**^{TMS}, CF_3H, and TMSF with the appropriate acceleration in stage III.

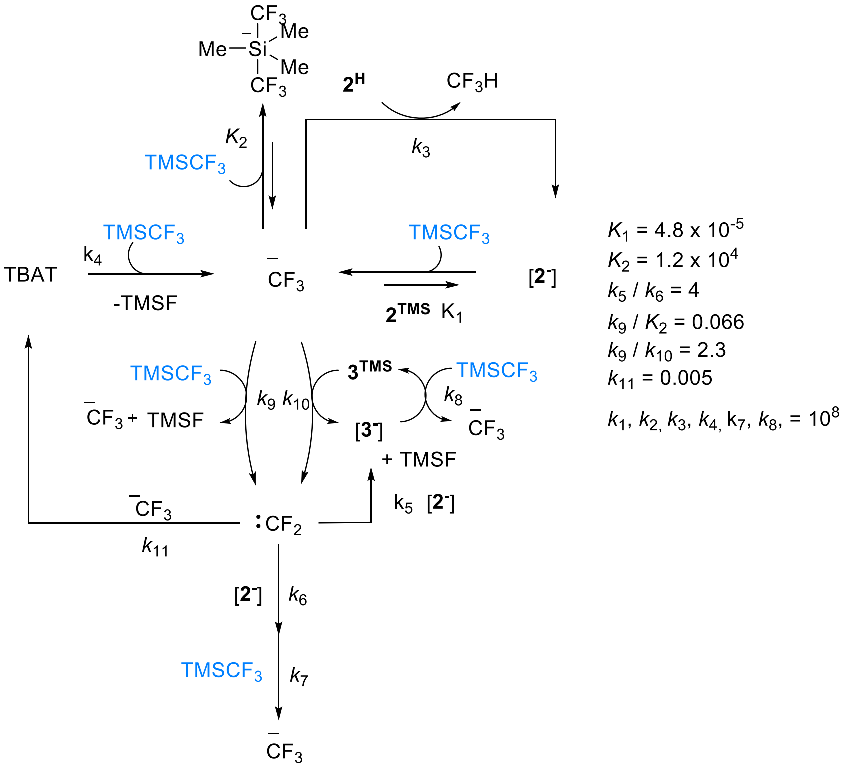

Scheme S2. Model used in kinetic simulations of CF_3H, **2e**^{TMS}, TMSF and **3e**^{TMS} shown in Figure S42. The model was fitted to experimental data using a standard numerical methods approach[S5] implemented through Excel with a proprietary scale-up systems add-in.

S11.2. Additional notes:

i. The model contains numerous simplifications, telescoped steps, and non-balanced steps (which do not corrupt the temporal concentrations of the species analyzed) and none of the values should be used in isolation.

ii. Numerous steps are set to be fast (10^8) and do not affect the fitting, but are essential for material balance / productivity.

iii. A complex series of inhibition pathways and regeneration of TBAT have been simplified to one reaction (k_{11}) where CF_2 + CF_3anion > TBAT.

iv. The rate of CF_2 generation (k_9/K_2)[CF_3][TMSCF_3] is that previously established.[S3]

v. The acceleration in stage III is very sensitive to the phenolate / phenol silyl ether equilibrium K_1, and required approximately 40% flexibility: 5.9 (+/- 1.1) × 10^{-5}, for fitting datasets 2, 3, and 4.

vi. Due to the anion-induced dynamic line broadening of TMSCF_3 during ^{19}F NMR monitoring, the temporal concentration data for TMSCF_3 was not included in the fitting.

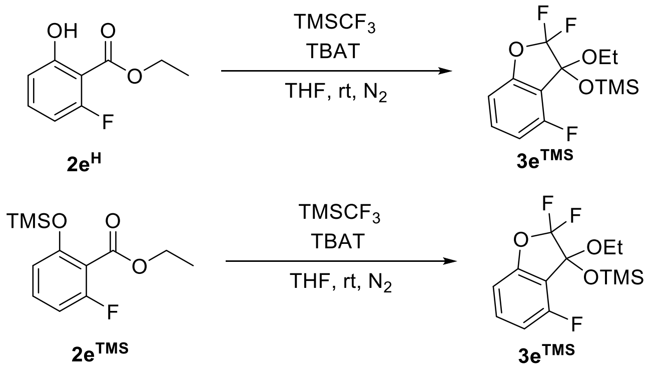

Dataset 1: [TMSCF_3]$_0$ = 0.49 M, 2e [**2e^{H}**]$_0$ = 0.175 M, [TBAT]$_0$ = 0.015 M

Dataset 2: [TMSCF_3]$_0$ = 0.47 M, 2e [**2e^{H}**]$_0$ = 0.18 M, [TBAT]$_0$ = 0.02 M

Dataset 3: [TMSCF_3]$_0$ = 0.25 M, 2e [**2e^{H}**]$_0$ = 0.19 M, [TBAT]$_0$ = 0.016 M

Dataset 4: [TMSCF_3]$_0$ = 0.23 M, 3e [**2e^{TMS}**]$_0$ = 0.19 M, [TBAT]$_0$ = 0.015 M

Dataset 5: [TMSCF_3]$_0$ = 0.71 M, 2e [**2e^{H}**]$_0$ = 0.19 M, [TBAT]$_0$ = 0.015 M

Dataset 6: [TMSCF_3]$_0$ = 0.42 M, 2e [**2e^{H}**]$_0$ = 0.1 M, [TBAT]$_0$ = 0.015 M

Dataset 7: [TMSCF_3]$_0$ = 0.46 M, 3e [**2e^{TMS}**]$_0$ = 0.18 M, [TBAT]$_0$ = 0.015 M

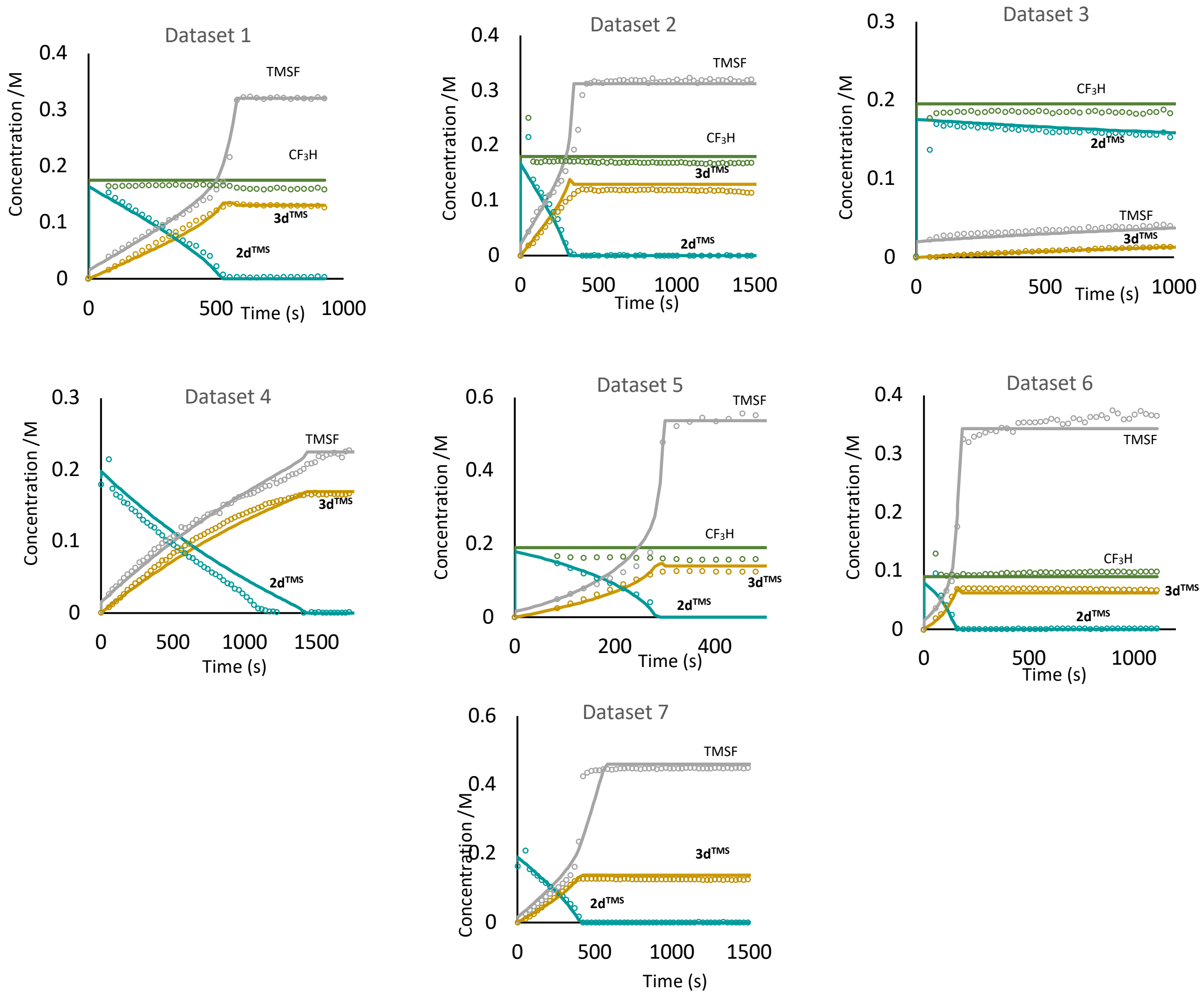

Figure S42. Kinetic simulations (solid lines) of CF_3H, **2e**^{TMS}, TMSF and **3e**^{TMS} across a variety of initial concentrations of reaction components **2e**^{H}, **2e**^{TMS}, $TMSCF_3$, and TBAT. These simulations were compared to experimental data (round data points) obtained by *in situ* ^{19}F NMR spectroscopic analysis, using PhF as the internal standard.

S11.3. Derivation of Steady State, Rapid Pre-Equilibrium Approximations

As shown in Scheme 9 in main manuscript, K_1 and K_2 are defined:

$$K_1 = \frac{[\mathrm{CF_3}^-][\mathbf{2}^{\mathrm{TMS}}]}{[\mathbf{2}^-][\mathbf{1}]} \; and\ thus\ [\mathbf{2}^-] = \frac{[\mathrm{CF_3}^-][\mathbf{2}^{\mathrm{TMS}}]}{K_1[\mathbf{1}]} \quad \text{(eq. S1)}$$

$$K_2 = \frac{[\mathbf{5}^-]}{[\mathrm{CF_3}^-][\mathbf{1}]} \; and\ thus\ [\mathbf{5}^-] = K_2\,[\mathrm{CF_3}^-][\mathbf{1}] \quad \text{(eq. S2)}$$

Assuming all TBAT is converted into active anions ($[\mathrm{TBAT}]_0 = [\mathrm{CF_3}^-] + [\mathbf{2}^-] + [\mathbf{5}^-]$):
From equations S1 and S2:

$$[\mathrm{TBAT}]_0 \approx [\mathrm{CF_3}^-] + \frac{[\mathrm{CF_3}^-][\mathbf{2}^{\mathrm{TMS}}]}{K_1[\mathbf{1}]} + K_2\,[\mathrm{CF_3}^-][\mathbf{1}] = [\mathrm{CF_3}^-]\left[1 + \frac{[\mathbf{2}^{\mathrm{TMS}}]}{K_1[\mathbf{1}]} + K_2\,[\mathbf{1}]\right] \quad \text{(eq. S3)}$$

$from\ eq.S3$:

$$[\mathrm{CF_3}^-] \approx \frac{[\mathrm{TBAT}]_0}{\left[1 + \frac{[\mathbf{2}^{\mathrm{TMS}}]}{K_1[\mathbf{1}]} + K_2\,[\mathbf{1}]\right]} = \frac{[\mathrm{TBAT}]_0 K_1[\mathbf{1}]}{K_1[\mathbf{1}] + [\mathbf{2}^{\mathrm{TMS}}] + K_1K_2[\mathbf{1}]^2} \quad \text{(eq. S4)}$$

$from\ eq.S1\ and\ S4$:

$$[\mathbf{2}^-] \approx \frac{[\mathbf{2}^{\mathrm{TMS}}]}{K_1[\mathbf{1}]}\frac{[\mathrm{TBAT}]_0 K_1[\mathbf{1}]}{K_1[\mathbf{1}] + [\mathbf{2}^{\mathrm{TMS}}] + K_1K_2[\mathbf{1}]^2} = \frac{[\mathrm{TBAT}]_0[\mathbf{2}^{\mathrm{TMS}}]}{K_1[\mathbf{1}] + [\mathbf{2}^{\mathrm{TMS}}] + K_1K_2[\mathbf{1}]^2} \quad \text{(eq. S5)}$$

As shown in Scheme 8 in main manuscript
Assuming steady state [CF_2]:

$$\frac{\mathrm{d}[\mathrm{CF_2}]}{\mathrm{d}t} \approx 0 = k_{\mathrm{F}}[\mathrm{CF_3}^-][\mathbf{1}] - [\mathrm{CF_2}](k_{\mathrm{O}}[\mathbf{2}^-] + k_{\mathrm{C}}[\mathrm{CF_3}^-]) \quad \text{(eq. S6)}$$

$$[\mathrm{CF_2}] \approx \frac{k_{\mathrm{F}}[\mathrm{CF_3}^-][\mathbf{1}]}{(k_{\mathrm{O}}[\mathbf{2}^-] + k_{\mathrm{C}}[\mathrm{CF_3}^-])} \quad \text{(eq. S7)}$$

Assuming steady state [$\mathbf{3}^-$]:

$$\frac{\mathrm{d}[3]^-}{\mathrm{d}t} \approx 0 = [\mathrm{CF_2}](k_{\mathrm{O}}[\mathbf{2}^-]) - k_{rxn}[\mathbf{3}^-][\mathbf{1}] \quad \text{(eq. S8)}$$

$$\frac{\mathrm{d}[\mathbf{3}^{\mathrm{TMS}}]}{\mathrm{d}t} \approx k_{rxn}[\mathbf{3}^-][\mathbf{1}] \quad \text{(eq. S9)}$$

From equations S8 and S9:

$$\frac{\mathrm{d}[\mathbf{3}^{\mathrm{TMS}}]}{\mathrm{d}t} \approx [\mathrm{CF_2}](k_{\mathrm{O}}[\mathbf{2}^-]) \quad \text{(eq. S10)}$$

From equations S7 and S10:

$$\frac{\mathrm{d}[\mathbf{3}^{\mathrm{TMS}}]}{\mathrm{d}t} \approx \frac{k_{\mathrm{F}}[\mathrm{CF_3}^-][\mathbf{1}]}{(k_{\mathrm{O}}[\mathbf{2}^-] + k_{\mathrm{C}}[\mathrm{CF_3}^-])}(k_{\mathrm{O}}[\mathbf{2}^-]) = \frac{k_{\mathrm{F}}[\mathrm{CF_3}^-][\mathbf{1}]}{(1 + \frac{k_{\mathrm{C}}[\mathrm{CF_3}^-]}{k_{\mathrm{O}}[\mathbf{2}^-]})} = \frac{1}{(1 + \frac{k_{\mathrm{C}}[\mathrm{CF_3}^-]}{k_{\mathrm{O}}[\mathbf{2}^-]})}\,k_{\mathrm{F}}[\mathrm{CF_3}^-][\mathbf{1}] \quad \text{(eq. S11)}$$

defining fractionation, *f*:

$$f = \frac{1}{\left(1+\frac{k_{\mathrm{C}}[\mathrm{CF_3}^-]}{k_{\mathrm{O}}[\mathbf{2}^-]}\right)} = \frac{1}{\left(1+\frac{k_{\mathrm{C}}K_1[\mathbf{1}]}{k_{\mathrm{O}}[\mathbf{2}^{\mathrm{TMS}}]}\right)} \; based\ on \frac{equation\ S4}{equation\ S5} \qquad \text{(eq. S12)}$$

from equations S4, S11 and S12:

$$\frac{\mathrm{d}[\mathbf{3}^{\mathbf{TMS}}]}{\mathrm{d}t} \approx f\, k_{\mathrm{F}}[\mathrm{CF_3}^-][\mathbf{1}] = f\frac{k_{\mathrm{F}}[\mathrm{TBAT}]_0 K_1[\mathbf{1}]^2}{K_1[\mathbf{1}] + [\mathbf{2}^{\mathrm{TMS}}] + K_1K_2[\mathbf{1}]^2} = f\frac{\frac{k_{\mathrm{F}}[\mathrm{TBAT}]_0}{K_2}}{\frac{1}{K_2[\mathbf{1}]} + \frac{[\mathbf{2}^{\mathrm{TMS}}]}{K_1K_2[\mathbf{1}]^2} + 1} \qquad \text{(eq. S13)}$$

$$\frac{\mathrm{d}[\mathbf{3}^{\mathbf{TMS}}]}{\mathrm{d}t} \approx f\frac{\frac{k_{\mathrm{F}}}{K_2}[\mathrm{TBAT}]_0}{\frac{[\mathbf{2}^{\mathrm{TMS}}]}{K_1K_2[\mathbf{1}]^2} + 1} \; when\ 1/K_2 \ll 1 \qquad \text{(equation 1 in main text)} \qquad \text{(eq. S14)}$$

Relative rates of conversion of silyl ethers **2*i***$^{\mathbf{TMS}}$ versus **2*ii***$^{\mathbf{TMS}}$ into **3*i***$^{\mathbf{TMS}}$ and **3*ii***$^{\mathbf{TMS}}$ respectively:

$$k_{rel\ i/ii} = \frac{\frac{\mathrm{d}[\mathbf{3}i^{\mathrm{TMS}}]}{\mathrm{d}t}}{\frac{\mathrm{d}[\mathbf{3}ii^{\mathrm{TMS}}]}{\mathrm{d}t}} \qquad \text{(eq. S15)}$$

Relative rates of conversion in a pair of *independent* reactions of **2*i***$^{\mathbf{TMS}}$ versus **2*ii***$^{\mathbf{TMS}}$:
From equation S14:

$$k_{rel\ i/ii}^{indep} = \frac{\frac{\mathrm{d}[\mathbf{3}i^{\mathrm{TMS}}]}{\mathrm{d}t}}{\frac{\mathrm{d}[\mathbf{3}ii^{\mathrm{TMS}}]}{\mathrm{d}t}} \approx \frac{f_{\mathrm{i}}\left[\frac{\frac{k_{\mathrm{F}}}{K_2}[\mathrm{TBAT}]_0}{1+\frac{[\mathbf{2}i^{\mathrm{TMS}}]}{K_{1i}K_2[\mathbf{1}]^2}}\right]}{f_{\mathrm{ii}}\left[\frac{\frac{k_{\mathrm{F}}}{K_2}[\mathrm{TBAT}]_0}{1+\frac{[\mathbf{2}ii^{\mathrm{TMS}}]}{K_{1ii}K_2[\mathbf{1}]^2}}\right]} = \frac{f_{\mathrm{i}}}{f_{\mathrm{ii}}}\left[\frac{1+\frac{[\mathbf{2}ii^{\mathrm{TMS}}]}{K_{1ii}K_2[\mathbf{1}]^2}}{1+\frac{[\mathbf{2}i^{\mathrm{TMS}}]}{K_{1i}K_2[\mathbf{1}]^2}}\right]\left[\frac{[\mathrm{TBAT}]_0^i}{[\mathrm{TBAT}]_0^{ii}}\right] \qquad \text{(eq. S16)}$$

$$\text{let } r = \frac{K_2[\mathbf{1}]^2}{\mathbf{2}i^{\mathrm{TMS}}} \qquad \text{(eq. S17)}$$

$$k_{rel\frac{i}{ii}}^{indep} \approx \left[\frac{r+\frac{1}{K_{1ii}}}{r+\frac{1}{K_{1i}}}\right] when\ \frac{f_{\mathrm{i}}}{f_{\mathrm{ii}}} = \frac{[\mathbf{2}i^{\mathrm{TMS}}]}{[\mathbf{2}ii^{\mathrm{TMS}}]} = \frac{[\mathrm{TBAT}]_0^i}{[\mathrm{TBAT}]_0^{ii}} = 1, \text{ (equation 2 in main text)} \qquad \text{(eq. S18)}$$

Relative rates of conversion in a single *competition* reaction between silyl ethers **2*i***$^{\mathbf{TMS}}$ versus **2*ii***$^{\mathbf{TMS}}$:
from equations S5 and S10:

$$k_{rel\frac{i}{ii}}^{comp} = \frac{\frac{\mathrm{d}[\mathbf{3}i^{\mathrm{TMS}}]}{\mathrm{d}t}}{\frac{\mathrm{d}[\mathbf{3}ii^{\mathrm{TMS}}]}{\mathrm{d}t}} \approx \frac{[\mathrm{CF_2}]\, k_{Oi}[\mathbf{2}i^-]}{[\mathrm{CF_2}]k_{Oii}[\mathbf{2}ii^-]} = \frac{k_{Oi}}{k_{Oii}}\frac{\frac{[\mathrm{CF_3}^-][\mathbf{2}i^{\mathrm{TMS}}]}{K_{1i}[\mathbf{1}]}}{\frac{[\mathrm{CF_3}^-][\mathbf{2}ii^{\mathrm{TMS}}]}{K_{1ii}[\mathbf{1}]}} = \frac{k_{Oi}}{k_{Oii}}\frac{K_{1ii}}{K_{1i}}\frac{[\mathbf{2}i^{\mathrm{TMS}}]}{[\mathbf{2}ii^{\mathrm{TMS}}]} \qquad \text{(eq. S19)}$$

$$k_{rel\frac{i}{ii}}^{comp} = \frac{k_{Oi}}{k_{Oii}}\frac{K_{1ii}}{K_{1i}}, \quad when\ \frac{[\mathbf{2}i^{\mathrm{TMS}}]}{[\mathbf{2}ii^{\mathrm{TMS}}]} = 1 \qquad \text{(equation 3 in main text)} \qquad \text{(eq. S20)}$$

Rate of CF_2 generation with additional surrogate fluoride acceptance by **2**$^{\mathbf{TMS}}$:

$$\frac{\mathrm{d}[\mathbf{3}^{\mathbf{TMS}}]}{\mathrm{d}t} \approx f\,(k_{\mathrm{F}}[\mathrm{CF_3}^-][\mathbf{1}] + k_{\mathrm{SF}}[\mathrm{CF_3}^-][\mathbf{2}^{\mathbf{TMS}}]) \approx f\,\frac{[\mathrm{TBAT}]_0(\frac{k_F}{K_2}[\mathbf{1}] + \frac{k_{SF}}{K_2}\frac{[\mathbf{2}^{\mathrm{TMS}}]}{[\mathbf{1}]})}{\left[1+\frac{[\mathbf{2}^{\mathrm{TMS}}]}{K_1\,K_2[\mathbf{1}]^1}\right]} \qquad \text{(eq. S21)}$$

When $\frac{k_F}{k_{\mathrm{SF}}}$ < 20 the quality of the fitting of the calc. versus exp. data shown in Figure 4 in the main text is corrupted. Linear regression using the three variables ($\frac{k_{SF}}{K_2}$; $\frac{k_F}{K_2}$; $K_1\ K_2$) raises $\frac{k_F}{k_{\mathrm{SF}}}$ to 166, indicative of negligible contribution from surrogate fluoride acceptance (k_{SF}) by **2b,e**$^{\mathbf{TMS}}$ in stage II.

S11.4. Analysis of Initial Rate of Generation of 3b,e^{TMS} and Correlation with Steady State Approximation; Figure 4 in main manuscript.

Reactions of **2b**^{H}, **2e**^{H}, and **2b**^{TMS} were set up at various initial concentrations, Table S1, using the standard methodologies described in Section S1, and then analyzed by *in situ* ^{19}F NMR at 300 K immediately after initiation with TBAT. The rate of evolution of **3b,e**^{TMS} and the concentrations of [**1**]$_t$ and [**2b,e**^{TMS}]$_t$ in earliest stable phase of stage II were estimated by integration and normalization against the internal standard, Table S5, entries 1 to 15. The rate reported in entry 3 was found to be an outlier from the correlation and excluded from the fitting process.

Table S5 Initial conditions, stage II conditions, and experimental and calculated rates, for the reactions of **2b**^{H}, **2e**^{H}, and **2b**^{TMS} with $TMSCF_3$ (**1**) in THF at 300 K analyzed by *in situ* ^{19}F NMR immediately after initiation with TBAT.

entry	Salicylate	[**1**]$_0$ / M	[**2**^{H}]$_0$ / M	[**2**^{TMS}]$_0$ / M	[TBAT]$_0$ / M	[**1**]$_t$ / M	[**2**^{TMS}]$_t$ / M	d[**3**^{TMS}]/dt exp. /Ms^{-1} x 10^{-4}	d[**3**^{TMS}]/dt calc. /Ms^{-1} x 10^{-4}
1	**2b**	0.48	0.20	-	0.015	0.3	0.2	2.6	2.6
2	**2b**	0.65	0.20	-	0.015	0.44	0.2	4.0	3.7
3	**2b**	0.23	0.20	-	0.015	0.04	0.18	1.1	0.1
4	**2b**	0.50	0.20	-	0.022	0.3	0.18	3.9	4.0
5	**2b**	0.50	0.10	-	0.015	0.4	0.1	4.6	4.4
6	**2b**	1.00	0.20	-	0.015	0.8	0.2	5.0	5.1
7	**2b**	0.50	-	0.2	0.015	0.36	0.16	3.4	3.4
8	**2b**	0.24	-	0.2	0.015	0.23	0.2	1.8	1.8
9	**2b**	1.80	0.20	-	0.015	1.5	0.2	5.8	5.8
10	**2b**	0.50	0.10	0.2	0.015	0.22	0.3	1.4	1.3
11	**2b**	0.65	-	0.2	0.015	0.4	0.2	3.3	3.4
12	**2e**	0.50	0.20	-	0.015	0.22	0.18	0.4	0.4
13	**2e**	0.70	0.20	-	0.015	0.45	0.2	1.3	1.2
14	**2e**	1.20	0.20	-	0.015	0.86	0.2	2.7	2.8
15	**2e**	0.50	0.10	-	0.015	0.3	0.06	1.7	1.6

Linear regression of the rates calculated using equation S14 (equation 4 in main manuscript), with k_F/K_2 (entries 1,2,4 to 15), $K_{1b}K_2$ (entries 1,2,4 to 11) and $K_{1e}K_2$ (entries 12 to 15) as fitting variables, afforded the correlation shown in Figure 4. The fitting was conducted to minimize the non-weighted sum square error across the set, and the two fitting parameters individually varied to minimize the sum square error for each entry and estimate the standard deviations in the fitting parameters k_F/K_2 = 4.2 (+/- 0.2) x 10^{-2} s^{-1}, $K_{1b}K_2$ = 1.5 (+/- 0.2) M^{-1} and $K_{1e}K_2$ = 0.2 (+/- 0.02) M^{-1}. The standard deviation in the ratio d[**3**^{TMS}]/dt calc. / d[**3**^{TMS}]/dt using the holistic fitting parameters was then used to calculate the overall correlation as: d[**3**^{TMS}]/dt calc. = 0.99 +/- 0.05 d[**3**^{TMS}]/dt exp.

S12. Experimental

General Procedure for Esterification:

Salicylic acid dissolved in ethanol in oven dried flask. Concentrated H_2SO_4 was added to the solution, and the mixture refluxed at 88 °C for 16 h. The reaction mixture was neutralised with NaOH (1 M) and concentrated *in vacuo*. The organic layer was washed with water and the aqueous layer was extracted with EtOAc. The combined organic phases were washed with brine, dried over $MgSO_4$, filtered and concentrated *in vacuo*. The resulting crude product was purified by distillation to afford the desired ethyl ester.

Ethyl 6-fluoro-2-hydroxybenzoate, 2e^H

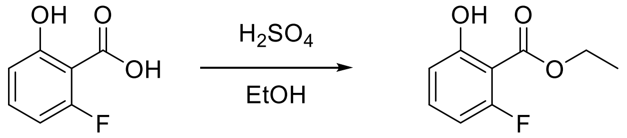

Following the general procedure, 6-fluorosalicylic acid (3 g, 20 mmol) was dissolved in ethanol (30 mL) and concentrated H_2SO_4 (6 mL) was added. The resulting crude product was purified by distillation (55 °C, 13 mbar). This afforded the title compound (2 g, 10.8 mmol, 54%) as a colourless oil.

^{1}H NMR (400 MHz, $CDCl_3$) δ: 11.35 (s, 1H), 7.4-7.35 (td, *J* = 8.4, 6.0 Hz, 1H), 6.81-6.78 (dt, *J* = 8.4, 1.0 Hz, 1H), 6.64-6.59 (ddd, *J* = 8.3, 1.1 Hz, 1H), 4.48 (q, *J* = 7.2 Hz, 2H), 1.45 (t, *J* = 7.2 Hz, 3H).

^{19}F NMR (376 MHz $CDCl_3$) δ: -104.8 (dd, *J* = 6.0, 4.9 Hz).

^{13}C{^{1}H} NMR (101 MHz, $CDCl_3$) δ: 169.4 (s), 163.0 (m), 161.1 (m), 135.2 (dd, *J* = 161, 12 Hz), 114.1 (s), 112.5 (s), 107 (m), 62.0 (t, *J* = 151 Hz), 14.0 (q, *J* = 126 Hz).

IR υ_{max} (film, cm^{-1}): 2962, 2904, 1731, 1614, 1464, 1304, 1252, 1235, 1103, 1040, 842, 792, 754, 702.

HRMS (ESI) m/z: $[M + H]^+$ calc'd for $C_9H_9O_3F$: 184.0536; found: 184.0528

Ethyl 5-fluoro-2-hydroxybenzoate, 2d^H

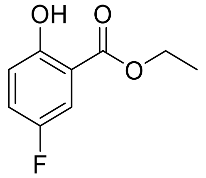

Following the general procedure, 5-fluorosalicylic acid (12.5 g, 80 mmol) was dissolved in ethanol (150 mL) and concentrated H_2SO_4 (22 mL) was added. The resulting crude product was purified by distillation (95 °C, 10 mbar). This afforded the title compound (10.9 g, 59.8 mmol, 77%) as a colourless oil.

^{1}H NMR (400 MHz, $CDCl_3$) δ: 10.62 (s, 1H), 7.55-7.52 (dd, J = 8.8, 3.2 Hz, 1H), 7.43-7.38 (m, 1H), 7.03-7.00 (dd, J = 9.15, 4.57 Hz), 4.36 (q, *J* = 7.2 Hz, 2H), 1.33 (t, *J* = 7.2 Hz, 3H).

^{19}**F NMR** (376 MHz $CDCl_3$) δ: -124.34 (m, 1H).

^{13}**C NMR** (101 MHz, $CDCl_3$) δ: 169.4 (m), 157.92 (m), 155.1 (m), 123.2 (ddd, J = 161, 25.2, 6.6 Hz), 118.8 (dt, J = 164, 7.1 Hz), 115.1 (ddd, J = 164, 23.4, 7.1 Hz), 112.6 (s), 61.8 (t, J = 150 Hz), 14.1 (d, J = 127 Hz).

IR $υ_{max}$ (film, cm^{-1}): 3171, 2986, 1678, 1619, 1481, 1404, 1373, 1329, 1279, 1252, 1192, 1071, 1015, 945, 884, 826, 779, 764, 679.

HRMS (ESI) m/z: $[M + H]^+$ calc'd for $C_9H_9O_3F$: 184.0536; found: 184.0527

Ethyl 4-fluoro-2-hydroxybenzoate, 2c^{H} [S6]

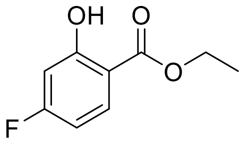

Following the general procedure, 4-fluorosalicylic acid (3.8 g, 25 mmol) was dissolved in ethanol (75 mL) and concentrated H_2SO_4 (12 mL) was added. The resulting crude product was purified by distillation (40 °C, 15 mbar). This afforded the title compound (3.11 g, 16.8 mmol, 68%) as a colourless oil.

^{1}**H NMR** (400 MHz, $CDCl_3$) δ: 11.1 (s, 1H), 7.88-7.84 (dd, *J* = 8.8, 6.6 Hz, 1H), 6.70-6.66 (dd, *J* = 10.4, 2.4 Hz, 1H), 6.63-6.57 (dt, *J* = 8.2, 2.4 Hz, 1H), 4.42 (q, *J* = 7.0 Hz, 2H), 1.43 (t, *J* = 7.0 Hz, 3H).

^{19}**F NMR** (376 MHz $CDCl_3$) δ: -101.5 (q, *J* = 7.3 Hz, 1H).

^{13}**C NMR** (101 MHz, $CDCl_3$) δ: 170.2 (s), 167.4 (d, *J* = 253 Hz), 164.1 (d, *J* = 14 Hz), 133.1 (d, *J* = 10 Hz), 108.7 (d, *J* = 2.2 Hz). 106.8 (d, *J* = 23 Hz), 104.1 (d, *J* = 25 Hz), 62.1 (s), 14.1 (s).

This compound is consistent with the data reported in the literature.[S6]

Ethyl 3-fluoro-2-hydroxybenzoate, 2b^{H}

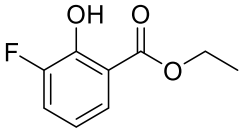

Following the general procedure, 3-fluorosalicylic acid (3 g, 20 mmol) was dissolved in ethanol (50 mL) and concentrated H_2SO_4 (10 mL) was added. The resulting crude product was purified by distillation (44 °C, 5 mbar). This afforded the title compound (2.15 g, 11.6 mmol, 58%) as a colourless oil.

^{1}**H NMR** (400 MHz, $CDCl_3$) δ: 10.9 (s, 1H), 7.67-7.64j (dt, *J* = 8.1, 1.5 Hz, 1H), 7.32-7.26 (m), 6.86-6.81 (td, *J* = 8.1, 4.6 Hz), 4.47 (q, *J* = 7.1 Hz, 2H), 1.45 (t, *J* = 7.1 Hz, 3H).

^{19}**F NMR** (376 MHz, $CDCl_3$) δ:-136.4 (ddd, *J* = 10.7, 4.6, 1.4 Hz)

^{13}**C{^{1}H} NMR** (101 MHz, $CDCl_3$) δ: 169.7, 152.7, 150.4, 124.8, 121.2, 118.2, 61.8, 34.3.

HRMS (ESI) m/z: $[M + H]^+$ calc'd for $C_9H_9O_3F$: 184.0536; found: 184.0527

IR υ_{max} (film, cm^{-1}): 3086, 3050, 2986, 1674, 1619, 1469, 1400, 1374, 1325, 1288, 1253, 1177, 1152, 1069, 1021, 842, 740, 636.

6-fluoro-2-trimethylsilanyloxy-benzoic acid ethyl ester, $2e^{TMS}$

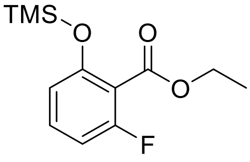

To a solution of ethyl 2-fluoro-6-hydroxybenzoate (0.46 g, 2.5 mmol) and Et_3N (0.28 g, 2.7 mmol) in THF (5 mL) under N_2 was added TMSCl (0.3 g, 3 mmol). The mixture was stirred at r.t. for 16 h. The reaction mixture was filtered through sintered glass and distilled under vacuum (93 °C, 5 mbar). This produced the title compound (0.2 g, 0.78 mmol, 32%) as a colourless oil.

^{1}H NMR (400 MHz, $CDCl_3$) δ: 7.27-7.21 (td, *J* = 8.3, 6.6 Hz, 1H), 6.76-6.72 (m, 1H), 6.67-6.64 (dt, *J* = 8.3, 0.8 Hz, 1H), 4.40 (q, *J* = 6.6 Hz, 2H), 1.40 (t, *J* = 7.2 Hz, 3H), 0.29 (s, 9H).

^{19}F NMR (376 MHz $CDCl_3$) δ: -113.7 (ddd, *J* = 8.9, 6.5, 0.7 Hz).

^{29}Si NMR (79.5 MHz, $CDCl_3$) δ: 22.6 (s).

^{13}C{^{1}H} NMR (101 MHz, $CDCl_3$) δ: 164.0, 161.6, 159.1, 131.1, 115.5, 108.8, 61.5, 14.3, 0.4.

IR υ_{max} (film, cm^{-1}): 2962, 1731, 1615, 1574, 1465, 1366, 1305, 1253, 1235, 1173, 1104, 1059, 1040, 842.

HRMS (ESI) m/z: $[M + H]^+$ calc'd for $C_{12}H_{17}O_3F^{28}Si$: 256.0931; found: 256.0925

5-fluoro-2-Trimethylsilanyloxy-benzoic acid ethyl ester, $2d^{TMS}$

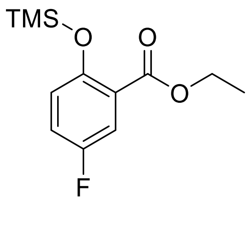

To a solution of ethyl 3-fluoro-6-hydroxybenzoate (0.19 g, 1 mmol) and Et_3N (0.11 g, 1.1 mmol) in THF (5 mL) under N_2 was added TMSCl (0.11 g, 1.05 mmol). The mixture was stirred at r.t. for 16 h. The reaction mixture was filtered through sintered glass and distilled under vacuum (96 °C, 3 mbar). This produced the title compound (0.15 g, 0.59 mmol, 59%) as a colourless oil.

^{1}H NMR (400 MHz, $CDCl_3$) δ: 7.53-7.50 (dd, *J* = 8.9, 3.2 Hz, 1H), 7.12-7.07 (ddd, *J* = 7.56, 7.52 Hz, 1H), 6.85-6.82 (dd, *J* = 8.9, 4.5 Hz, 1H), 4.36 (q, *J* = 7.1 Hz, 2H), 1.40 (t, *J* = 7.1 Hz, 3H), 0.28 (s, 9H).

^{19}F NMR (376 MHz $CDCl_3$) δ: -123.2 (ddd, *J* = 7.5, 4.5, 1.4 Hz)

^{29}Si NMR (79.5 MHz, $CDCl_3$) δ: 21.8

^{13}C{^{1}H} NMR (101 MHz, $CDCl_3$) δ: 165.2, 157.9, 155.6, 151.2, 122.7, 119.9, 117.6, 60.9, 14.1, 0.2.

IR υ_{max} (film, cm^{-1}): 2960, 1730, 1707, 1487, 1424, 1414, 1365, 1302, 1278, 1250, 1228, 1066, 894, 840.

HRMS (ESI) m/z: $[M + H]^+$ calc'd for $C_{12}H_{17}O_3F^{28}Si$: 256.0931; found: 256.0927

2-Trimethylsilanyloxy-benzoic acid ethyl ester, 2a^{TMS}

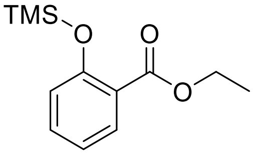

To a solution of ethyl 2-hydroxybenzoate (1.64 g, 10 mmol) and Et_3N (1.11 g, 11 mmol) in THF (5 mL) under N_2 was added TMSCl (1.14 g, 10.5 mmol). The mixture was stirred at r.t. for 16 h. The reaction mixture was filtered through sintered glass and distilled under vacuum (93 °C, 5 mbar). This produced the title compound (1.79 g, 7.5 mmol, 75%) as a colourless oil.

^{1}H NMR (400 MHz, $CDCl_3$) δ: 7.83-7.80 (dd, *J* = 7.6, 1.8 Hz, 1H), 7.4-7.35 (m, 1H), 7.03-6.99 (dt, *J* = 7.6, 1.1 Hz, 1H), 6.89-6.87 (dd, *J* = 8.2, 1.1 Hz, 1H), 4.38-4.33 (q, *J* = 7.0 Hz, 2H), 1.41-1.38 (t, *J* = 7.65 Hz, 3H) 0.28 (s, 9H).

^{29}Si NMR (79.5 MHz, $CDCl_3$) δ: 20.8 (s).

^{13}C{^{1}H} NMR (101 MHz, $CDCl_3$) δ: 153.5, 123.9, 122.5, 121.1, 119.4, 118.9, 117.8, 62.3, 13.8, 0.6.

IR υ_{max} (film, cm^{-1}): 2957, 1724, 1601, 1485, 1448, 1283, 1233, 1157, 1125, 1078, 1041, 918, 840, 757, 703, 668, 502.

HRMS (ESI) m/z: $[M + H]^+$ calc'd for $C_{12}H_{18}O_3{}^{28}Si$: 238.1025; found: 238.1019

2,2-difluoro-1-benzofuran-3(2H)-one, 4a [4]

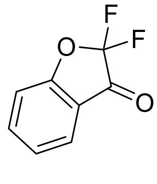

Under a N_2 atmosphere, TBAT (810 mg, 1.5 mmol) was added to ethyl salicylate (830 mg, 5 mmol) and $TMSCF_3$ (1.78 g, 12.5 mmol) in THF (150 mL). The reaction was stirred for 2 h. The reaction mixture was concentrated in vacuo yielding the desired product **4a**. The crude material was purified using column chromatography (eluent: Petroleum ether: DCM, 50:1) to afford **4a** as a pale yellow oil (323 mg, 1.7 mmol, 34%).

^{1}H NMR (400 MHz, $CDCl_3$) δ: 7.78 (m, 2H), 7.32-7.30 (dd, *J* = 8.2, 0.7 Hz, 1H), 7.23-7.20 (dt, *J* = 8.2, 0.7 Hz, 1H).

^{19}F NMR (376 MHz $CDCl_3$) δ: -94.1 (s).

^{13}C{^{1}H} NMR (101 MHz, $CDCl_3$) δ: 165.2, 142.1, 136.2, 134.9, 129.7, 127.7, 111.7, 109.2.

This compound is consistent with the data reported in the literature.[4]

5-fluoro-2,2-difluoro-1-benzofuran-3(2H)-one, 4e

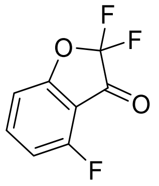

Under a N_2 atmosphere, TBAT (810 mg, 1.5 mmol) was added to ethyl 6-fluoro-2-hydoxybenzoate (600 mg, 3.25 mmol) and $TMSCF_3$ (1.15 g, 8.15 mmol) in THF (75 mL). The reaction was stirred for 2 h. The reaction mixture was concentrated in vacuo yielding the crude product **4e**. The crude material was purified using column chromatography (eluent: Petroleum ether: DCM, 60:40) to afford **4e** as a colourless oil (124 mg, 0.66 mmol, 20%).

^{1}**H NMR** (400 MHz, $CDCl_3$) δ: 7.61-7.59 (d, J = 7.0 Hz, 1H), 7.46-7.37 (m, 2H).

^{19}**F NMR** (376 MHz $CDCl_3$) δ:-92.6 (s, 2F), -104.4 (s, 1F).

^{13}**C{^{1}H} NMR** (101 MHz, $CDCl_3$) δ: 187.1, 167.0, 141.4, 132.5, 117.7, 114.1, 110.8, 106.6.

IR υ_{max} (film, cm^{-1}): 3071, 2958, 1759, 1631, 1606, 1497, 1448, 1325, 1309, 1193, 1114, 1015, 912, 842, 799, 770, 690.

HRMS (ESI) m/z: $[M + H]^+$ calc'd for $C_8H_3O_2F_3$: 188.0085; found: 188.0082

5-fluoro-2,2-difluoro-1-benzofuran-3(2H)-one, 4d

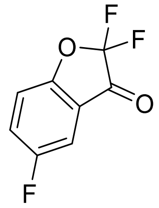

Under a N_2 atmosphere, TBAT (50 mg, 0.094 mmol) was added to ethyl 5-fluoro-2-hydroxybenzoate (200 mg, 1.25 mmol) and $TMSCF_3$ (444 mg, 3.12 mmol) in THF (3 mL). The reaction was stirred for 1.5 h. The reaction mixture was concentrated in vacuo yielding the crude product **4d**. The crude material was purified using column chromatography (eluent: Petroleum ether: DCM, 60:40) to afford **4d** as a yellow oil (230 mg, 1.25 mmol, 98%).

^{1}**H NMR** (400 MHz, $CDCl_3$) δ: 7.66-7.64 (m, 1H), 7.46-7.38 (m, 2H).

^{19}**F NMR** (376 MHz $CDCl_3$) δ:-93.4 (s, 2F), -115.7 (s, 1F).

^{13}**C{^{1}H} NMR** (101 MHz, $CDCl_3$) δ: 157.9, 135.4, 129.9, 127.8, 123.2, 118.8, 115.1, 111.7.

IR υ_{max} (film, cm^{-1}): 3071, 2980, 1770, 1679, 1623, 1482, 1429, 1329, 1243, 1209, 1144, 1114, 1070, 946, 827, 782, 698.

HRMS (ESI) m/z: $[M + H]^+$ calc'd for $C_8H_3O_2F_3$: 188.0085; found: 188.0082

Ethoxytrimethylsilane

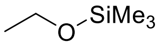

$TMSCF_3$ (3 mL, 20 mmol) was added to ethanol (2 mL, 34 mmol) and TBAT (1 g, 1.8 mmol) in THF (50 mL). A NMR spectra was recorded *in situ* to afford TMSOEt (188 mg, 1.6 mmol, 79%). The crude product was purified by vacuum distillation (22 °C, 700 mbar).

Due to the similar boiling point of TMSOEt and THF, both distilled together and therefore NMR spectra were recorded in THF.

^{1}H NMR (400 MHz, THF, solvent signals suppressed by standard pulse sequences) δ: 1.51 (q, *J* = 6.9 Hz, 2H), 1.33 (t, *J* = 6.9 Hz, 3H), 0.47 (s, 9H)

^{13}C{^{1}H} NMR (101 MHz, THF) δ: 57.1, 18.5, -0.99

^{29}Si-INEPT NMR (79.5 MHz, THF) δ: 15.3 (s)

HRMS (ESI) m/z: $[M + H]^+$ calc'd for $C_5H_{14}O^{28}Si$: 118.0814; found: 118.0726

S13. References

S1. C. P. Johnston, T. H. West, R. E. Dooley, M. Reid, A. B. Jones, E. J. King, A. G. Leach, and G. C. Lloyd-Jones, Anion-Initiated Trifluoromethylation by $TMSCF_3$: Deconvolution of the Siliconate-Carbanion Dichotomy by Stopped-Flow NMR/IR., *J. Am. Chem. Soc.,* **2018**, *140*, 11112-11124.

S2. A. García-Domínguez, P. H. Helou de Oliveira, G. T. Thomas, A. R. Sugranyes and G. C. Lloyd-Jones, Mechanism of Anion-Catalyzed C-H Silylation using $TMSCF_3$: Kinetically-Controlled CF_3-anionoid Partitioning as a Key Parameter., *ACS Catal.,***2021***, 11*, 3017–3025.

S3. A. García-Domínguez, T. H. West, J. J. Primozic, K. M. Grant, C. P. Johnston, G. G. Cumming, A. G. Leach and G. C. Lloyd-Jones, Difluorocarbene Generation from $TMSCF_3$: Kinetics and Mechanism of NaI-Mediated and Si-Induced Anionic Chain Reactions., *J. Am. Chem. Soc.* **2020**, *142*, 14649–14663.

S4. Y. Cai, W. Zhu, S. Zhao, C. Dong, Z. Xu and Y. Zhao, difluorocarbene-Mediated Cascade Cyclization: The Multifunctional Role of Ruppert–Prakash Reagent., *Org. Lett.* **2021**, *23*, 3546–3551.

S5. Ben-Tal, Y.; Boaler, P. J.; Dale, H. J. A; Dooley, R. E.; Fohn, N. A.; Gao, Y.; García-Domínguez, A.; Grant. K. M.; Hall, A. M. R.; Hayes, H. D. L.; Kucharski, M. M.; Wei, R.; Lloyd-Jones, G. C. Mechanistic Analysis by NMR Spectroscopy: a Users Guide, *Prog. Nucl. Magn. Reson. Spectrosc*., **2022,** 129, 28–106.

S6. Y. Yang, Y. Lin and Y. Rao., Ruthenium(II)-Catalyzed Synthesis of Hydroxylated Arenes with Ester as an Effective Directing Group., *Org. Lett.* **2012**, *14*, 2874–2877.

S14. ^{1}H, ^{13}C, $^{13}C\{^{1}H\}$, ^{19}F, ^{29}Si NMR Spectra and IR spectra:

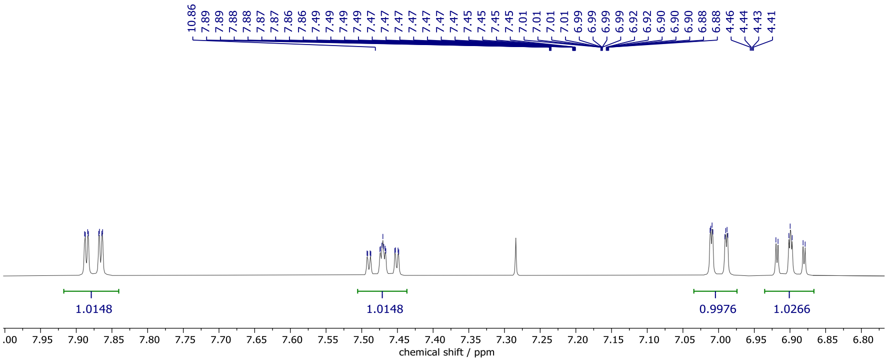

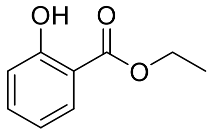

(2a^{H})

^{1}H NMR (400 MHz, $CDCl_3$)

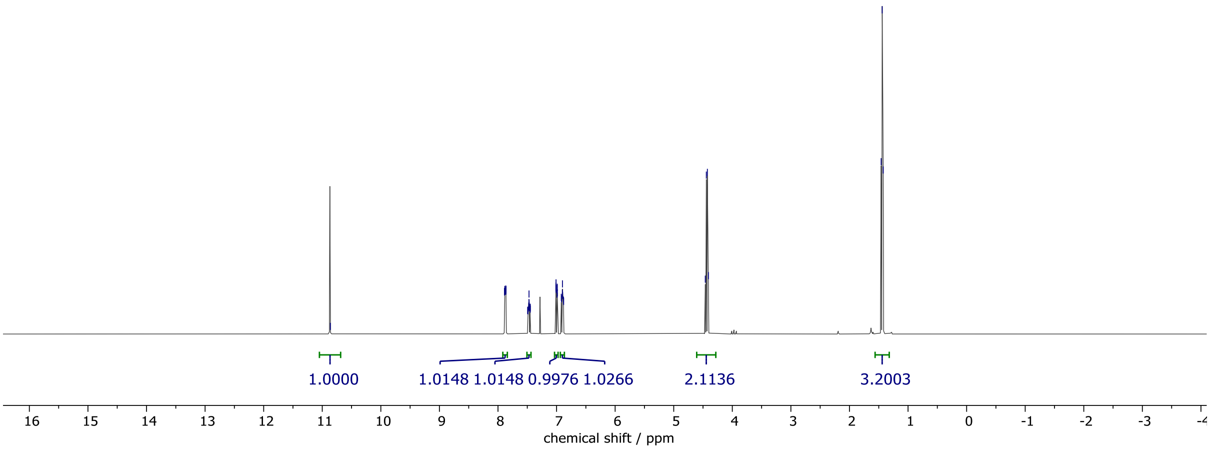

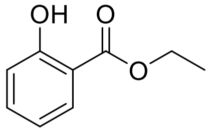

(2a^{H}) $^{13}C\{^{1}H\}$ NMR (101 MHz, $CDCl_3$)

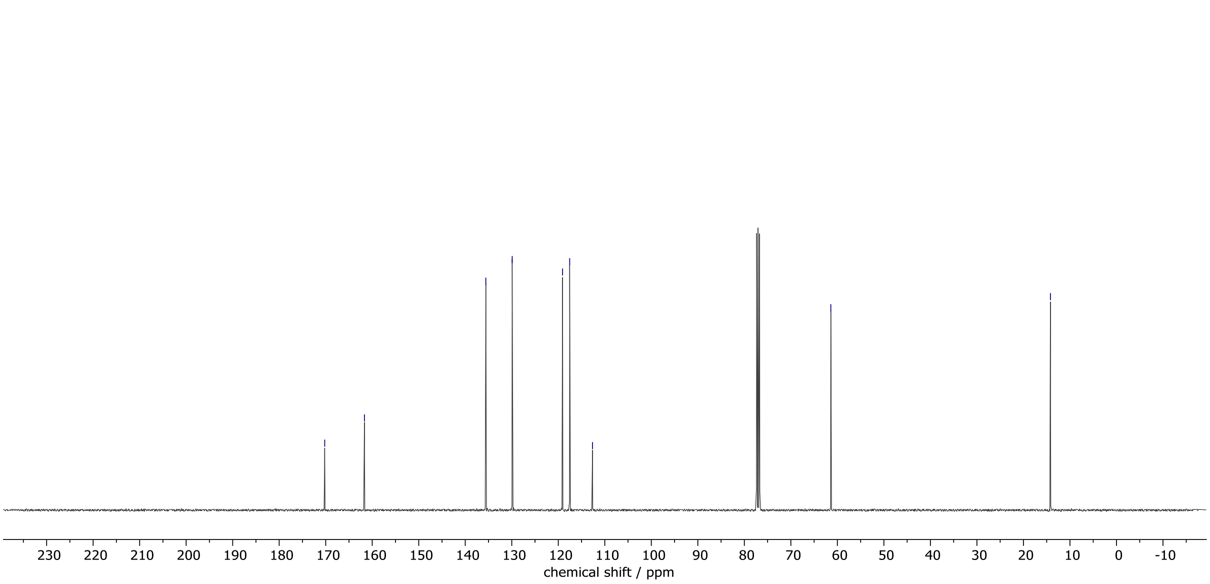

(2e^H) ¹H NMR (400 MHz, CDCl₃)

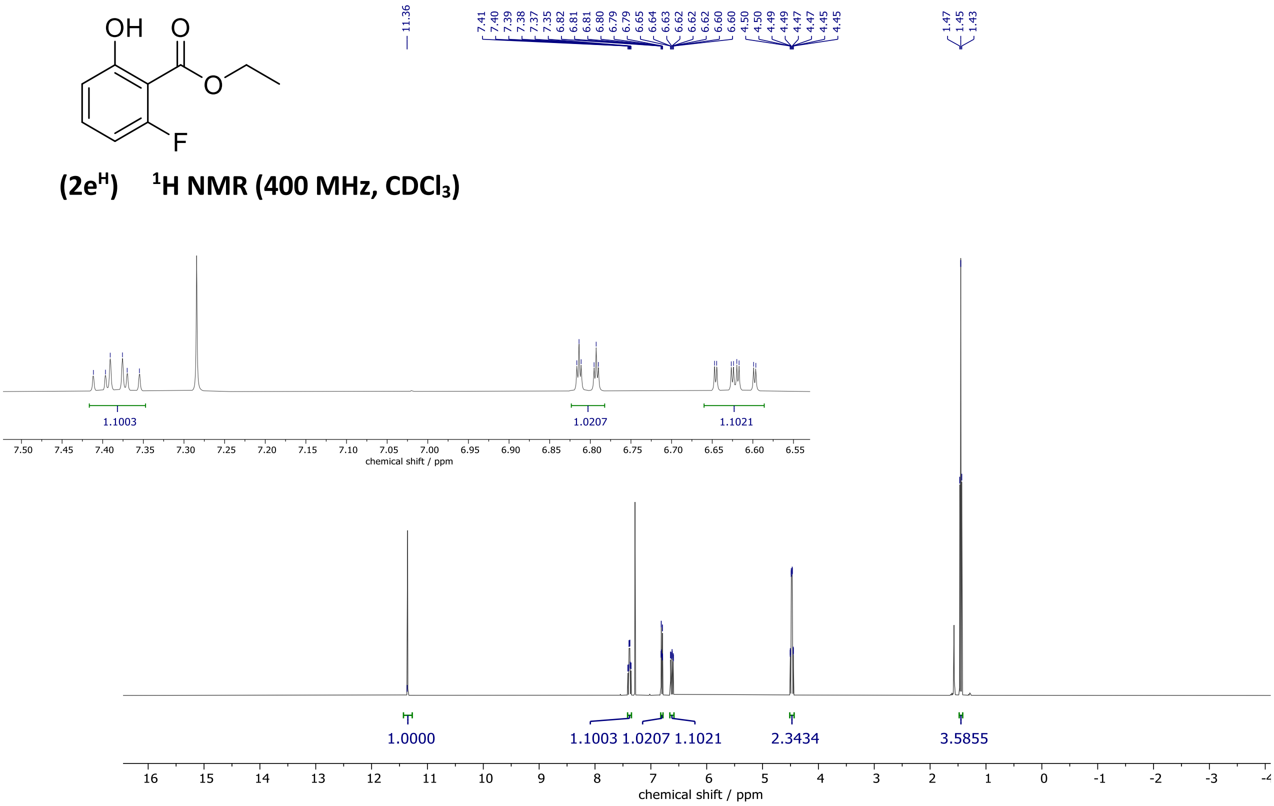

(2e^H) ¹⁹F NMR (376 MHz, CDCl₃)

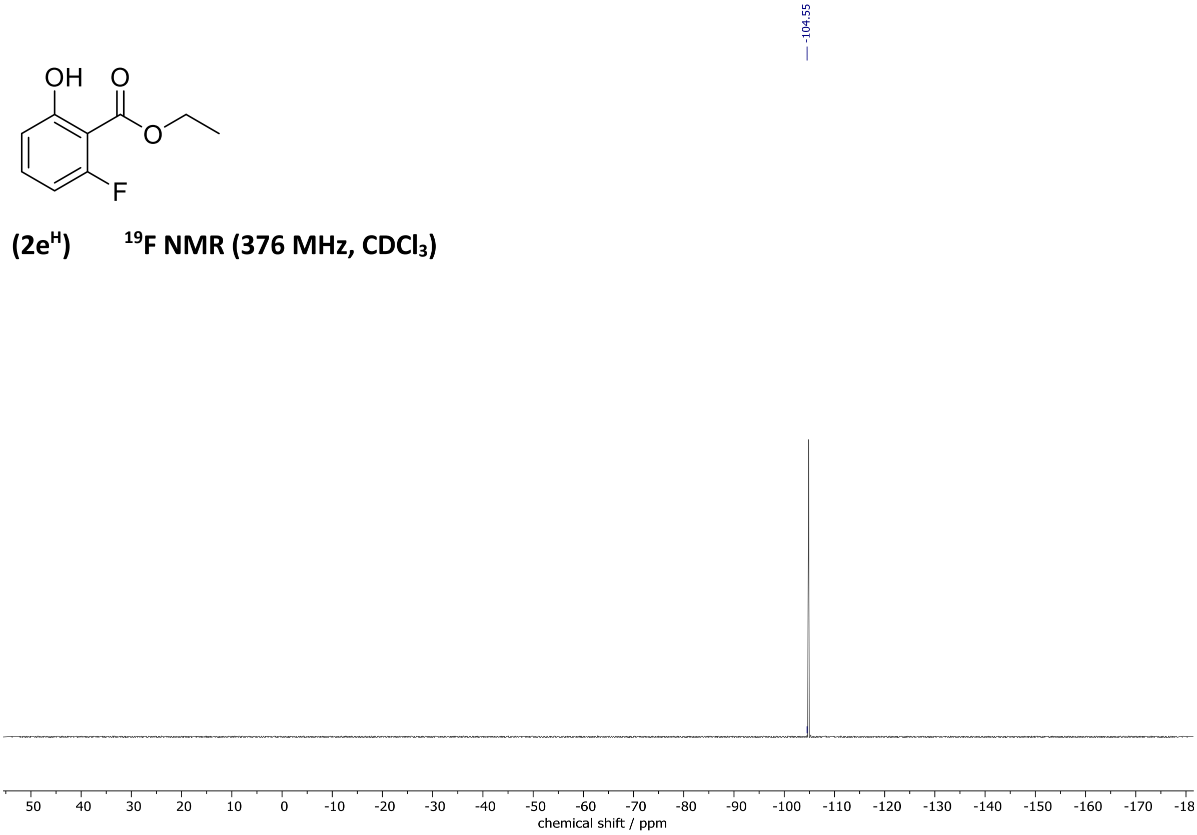

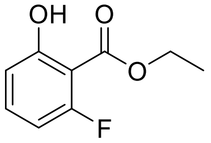

(2e^{H}) $^{13}C\{^{1}H\}$ NMR (101 MHz, $CDCl_3$)

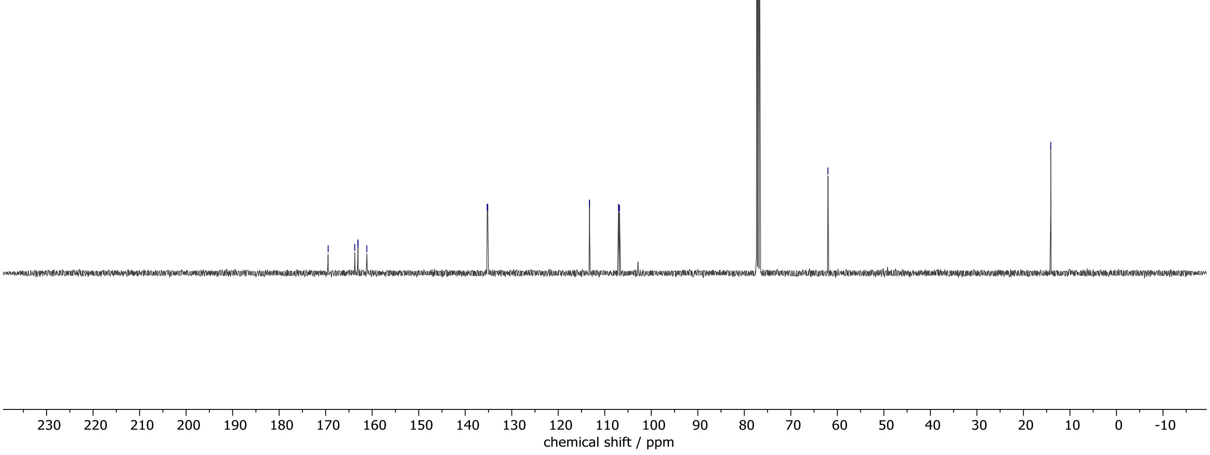

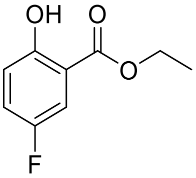

(2d^{H}) ^{1}H NMR (400 MHz, $CDCl_3$)

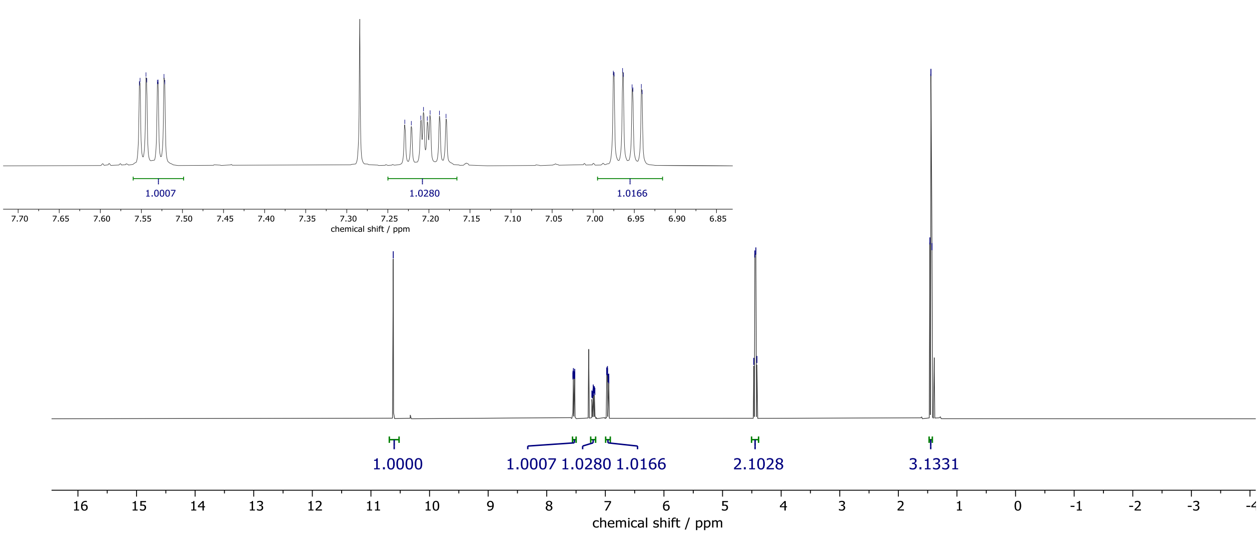

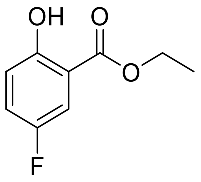

(2d^{H}) **^{19}F NMR (376 MHz, CDCl$_3$)**

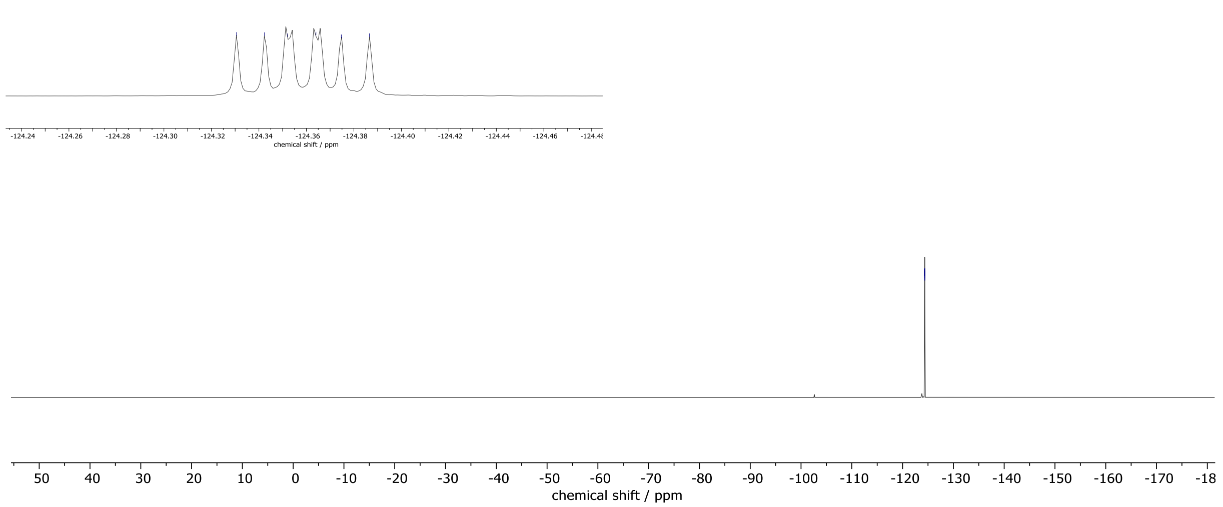

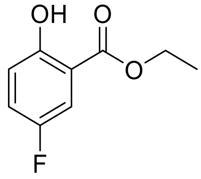

(2d^{H}) **^{13}C NMR (101 MHz, CDCl$_3$)**

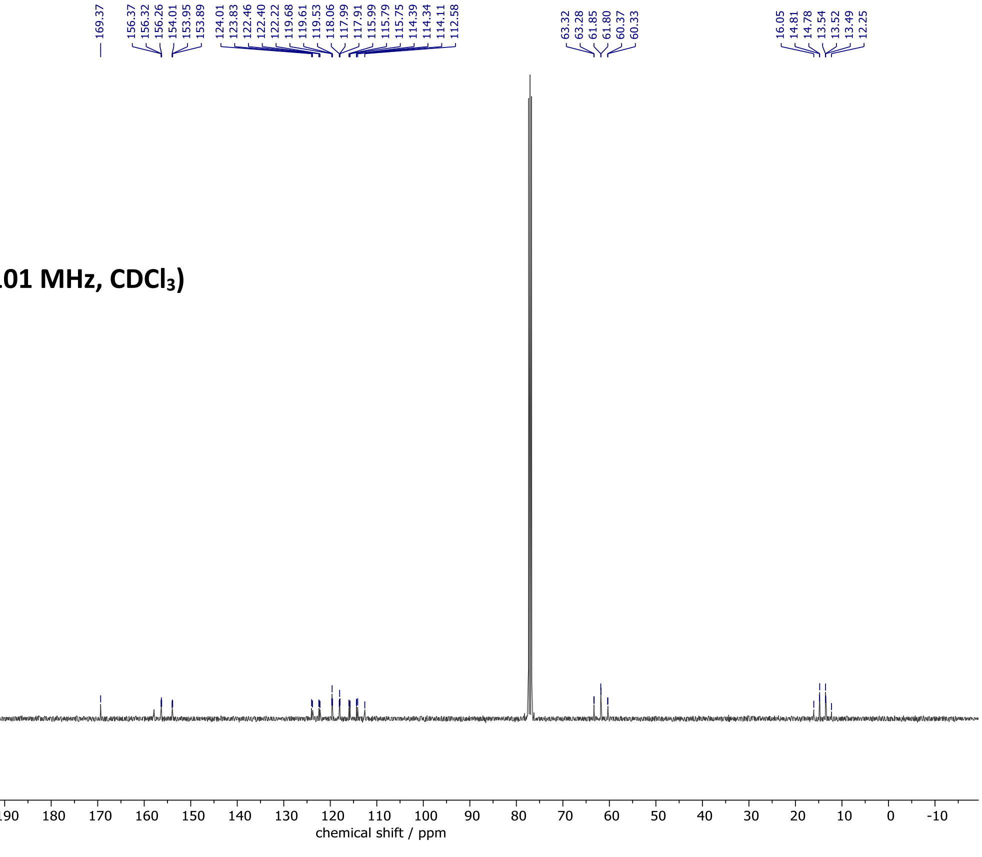

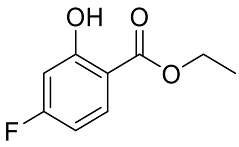

(2c^{H}) **^{1}H NMR (400 MHz, CDCl$_3$)**

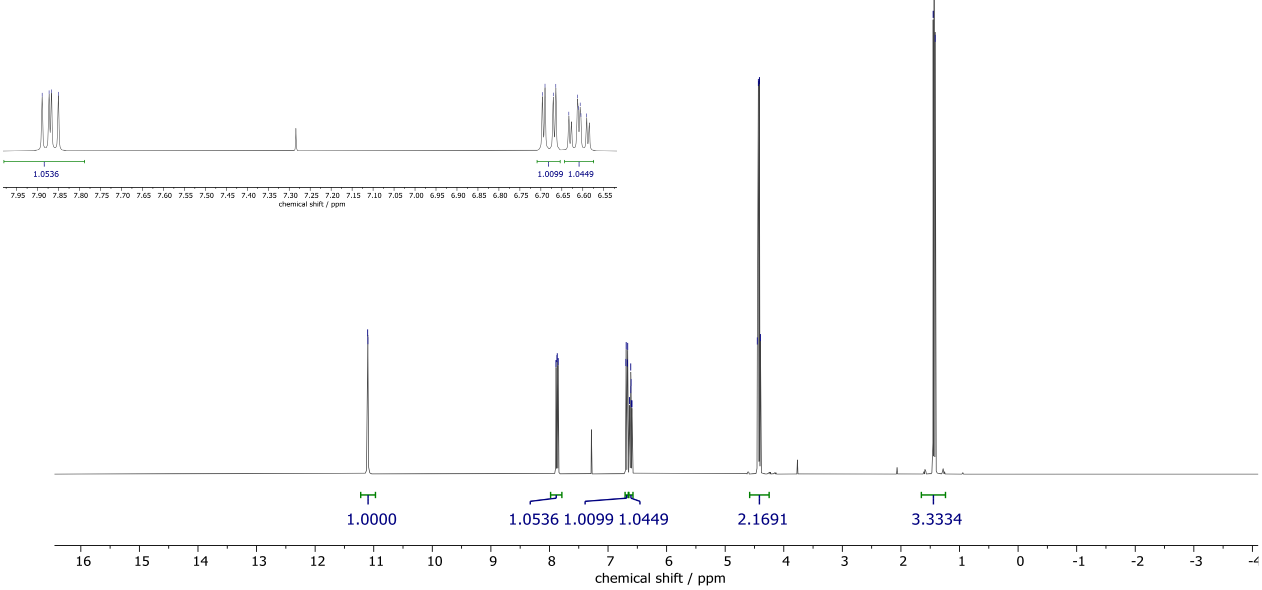

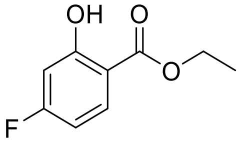

(2c^{H}) **^{19}F NMR (376 MHz, CDCl$_3$)**

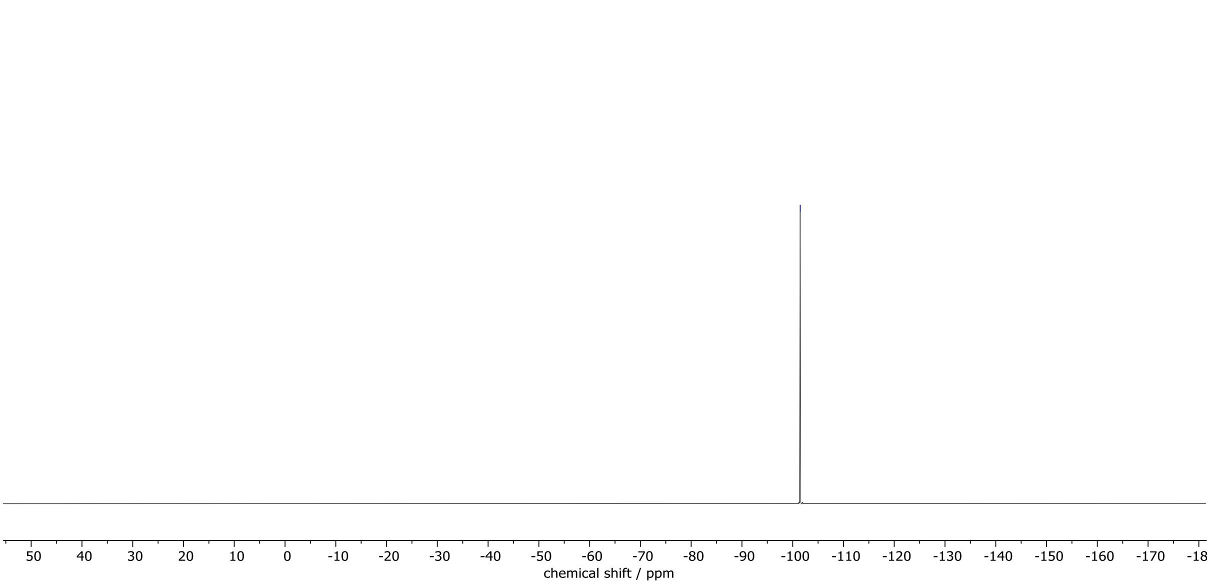

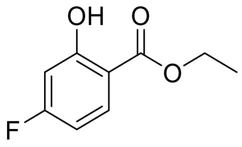

(2c^{H}) ^{13}C NMR (101 MHz, $CDCl_3$)

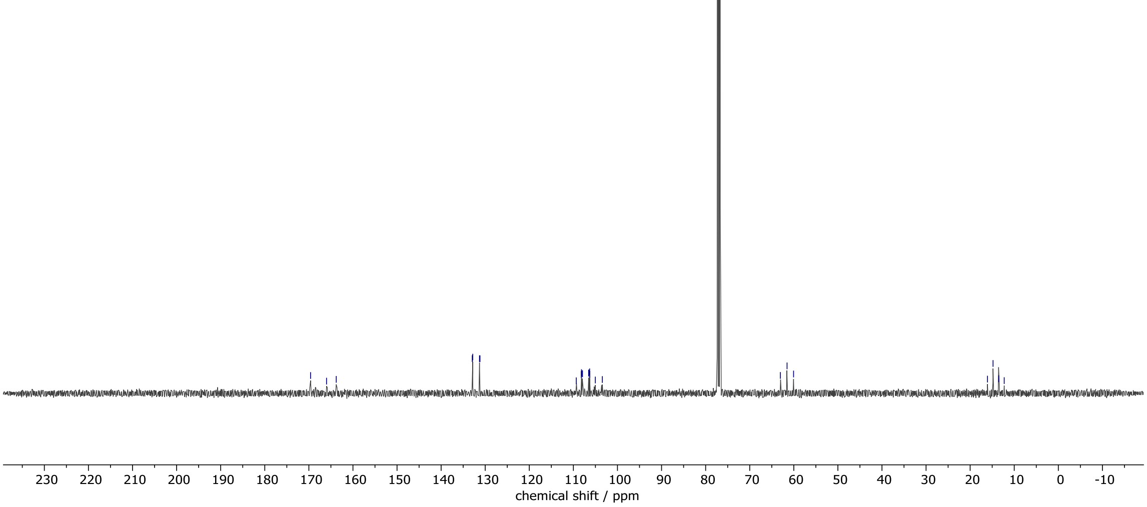

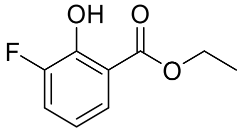

(2b^{H}) ^{1}H NMR (400 MHz, $CDCl_3$)

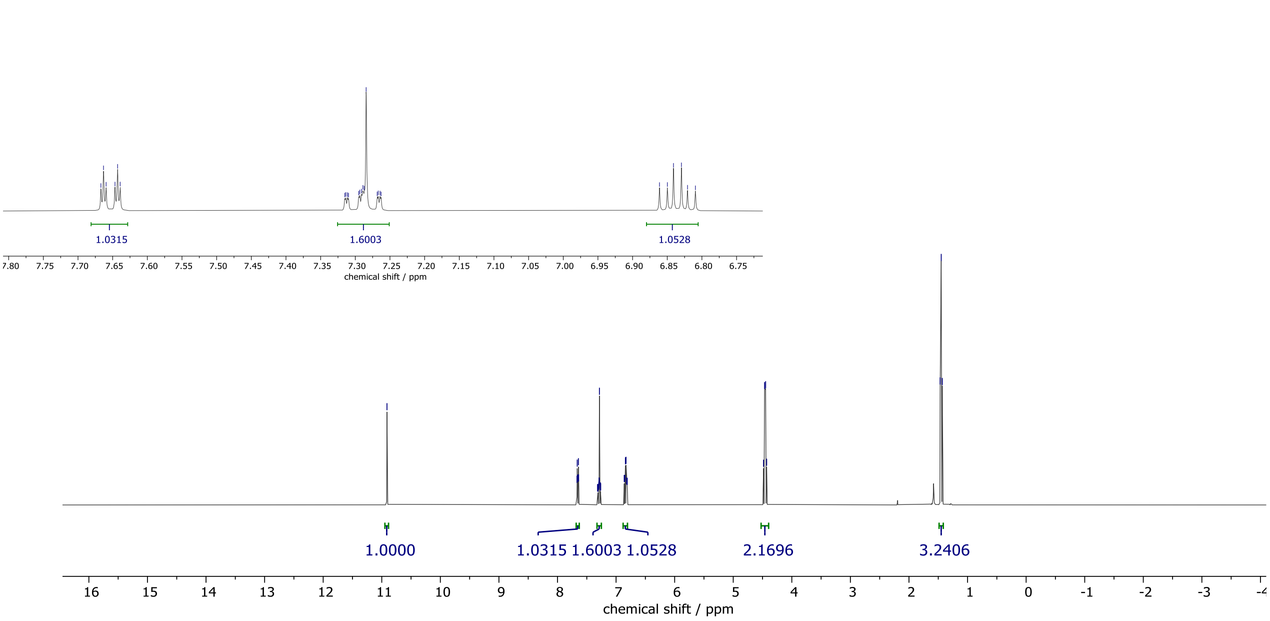

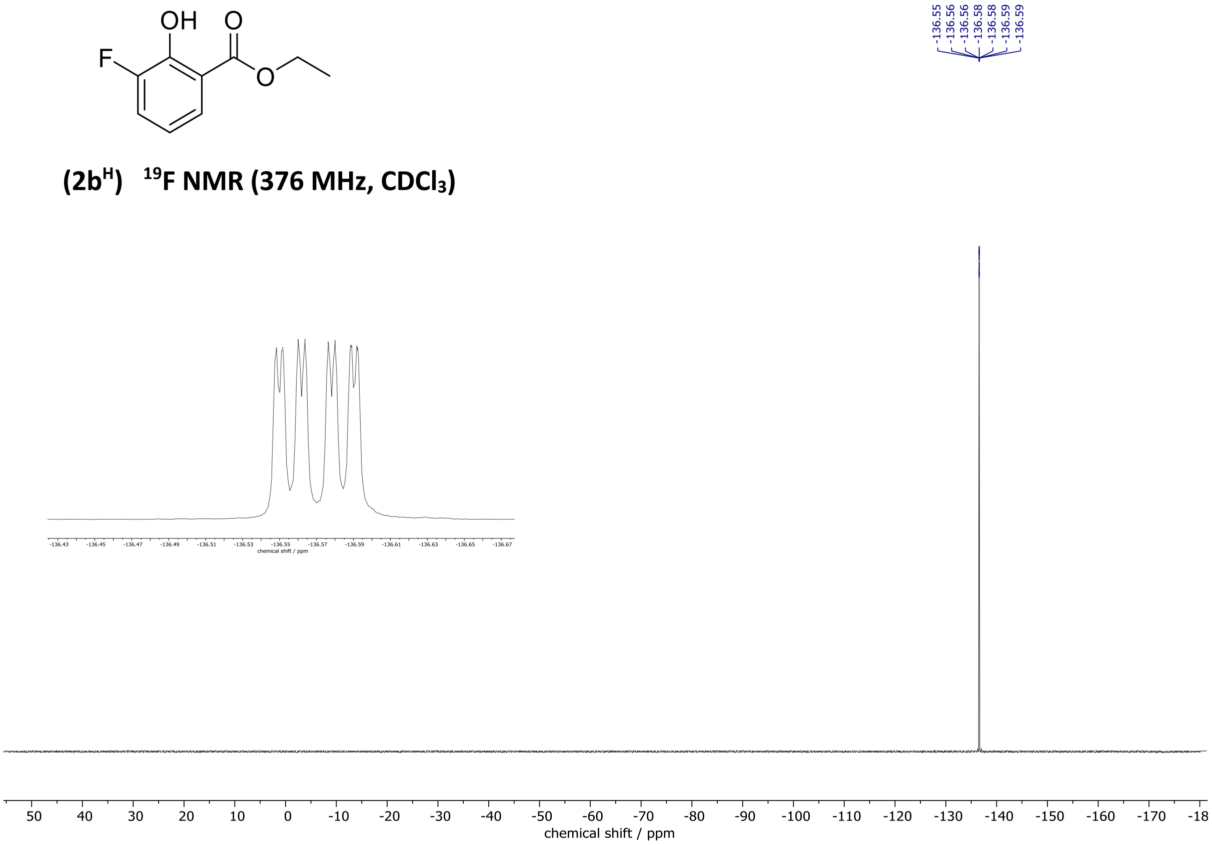

(2b^H) ^{19}F NMR (376 MHz, $CDCl_3$)

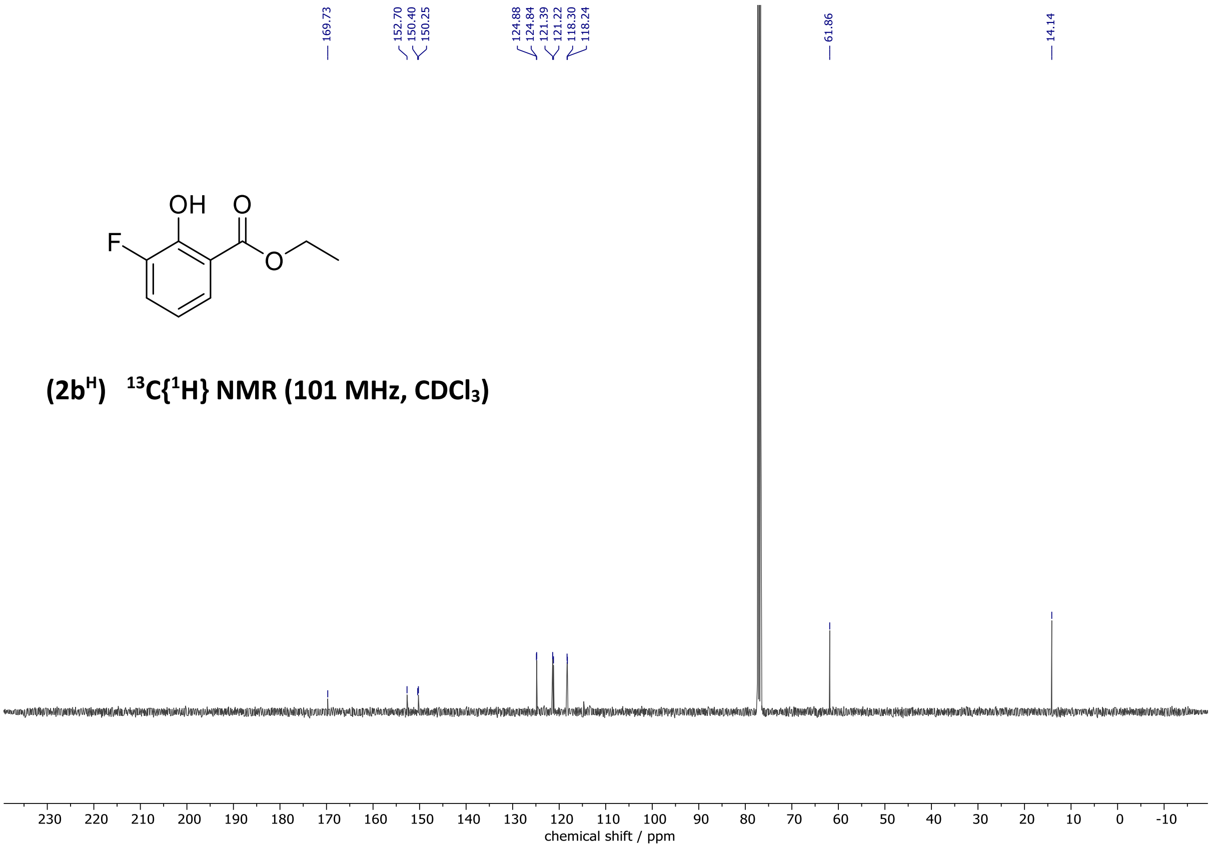

(2b^H) $^{13}C\{^1H\}$ NMR (101 MHz, $CDCl_3$)

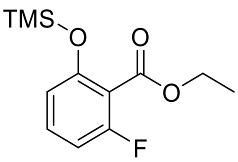

(2e^TMS) ^{1}H NMR (400 MHz, CDCl$_3$)

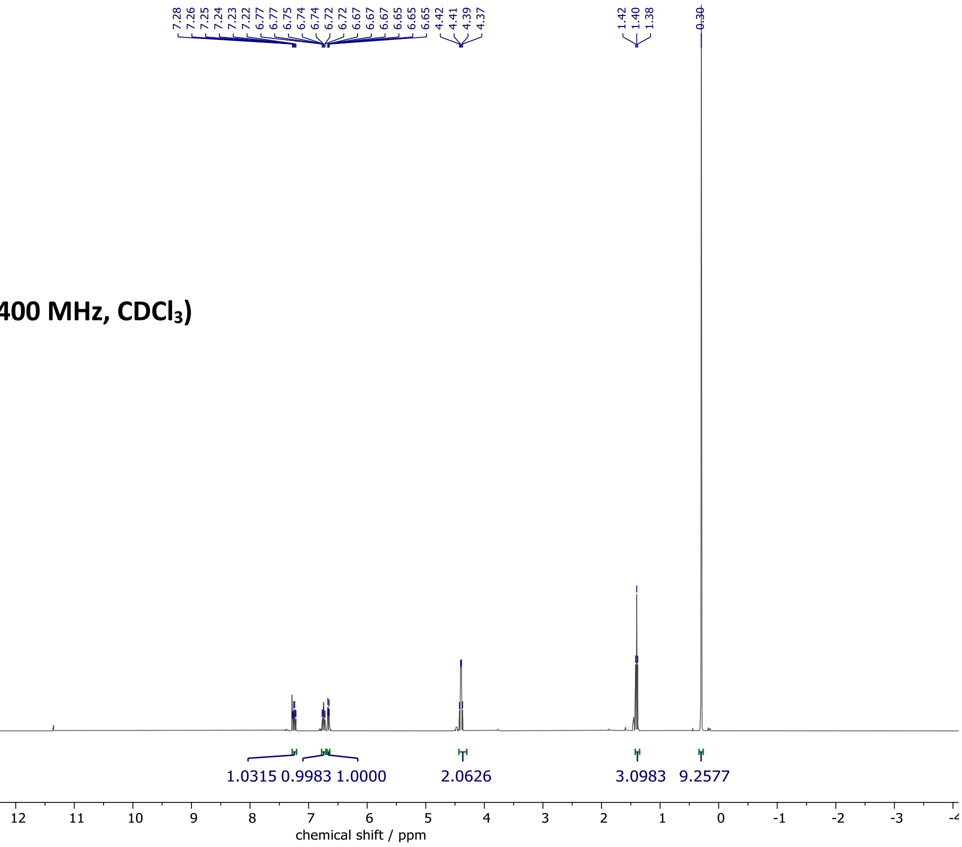

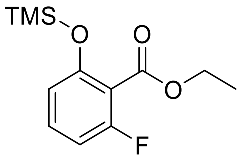

(2e^TMS) ^{19}F NMR (376 MHz, CDCl$_3$)

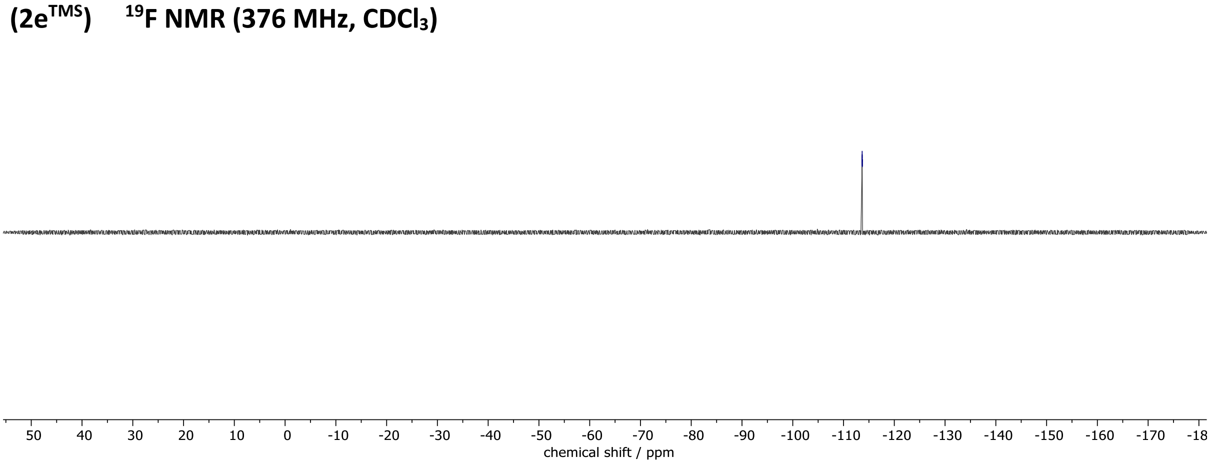

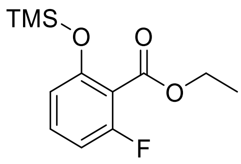

(2e[TMS]) ^{29}Si-INEPT NMR (79.5 MHz, CDCl$_3$)

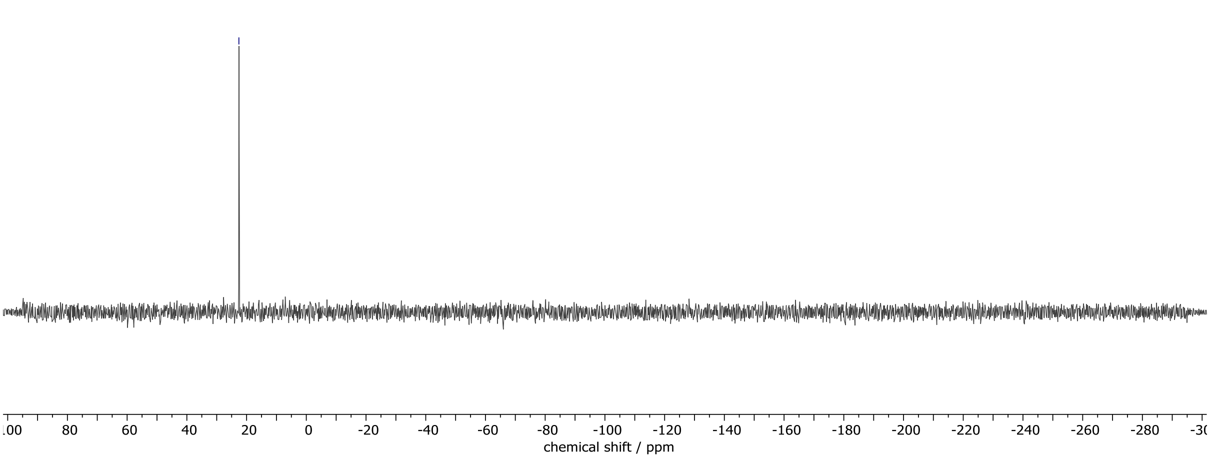

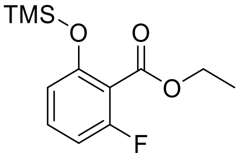

(2e[TMS]) ^{13}C{^{1}H} NMR (101 MHz, CDCl$_3$)

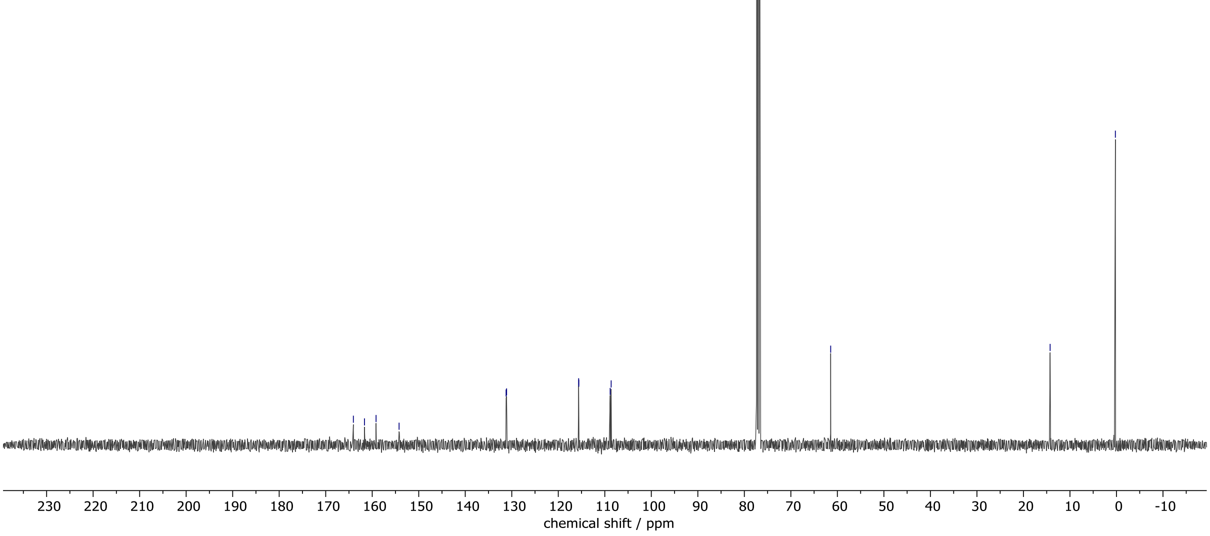

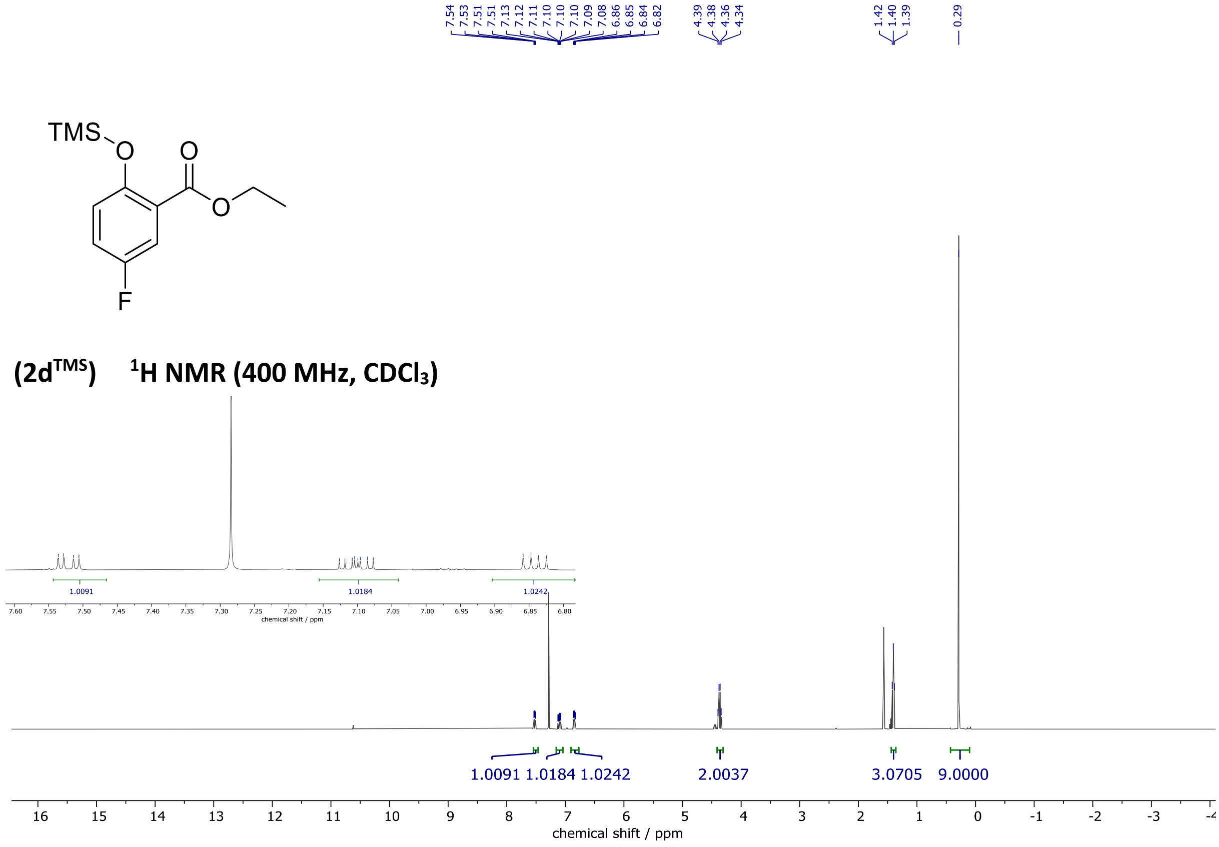

(2d[TMS]) ^{1}H NMR (400 MHz, $CDCl_3$)

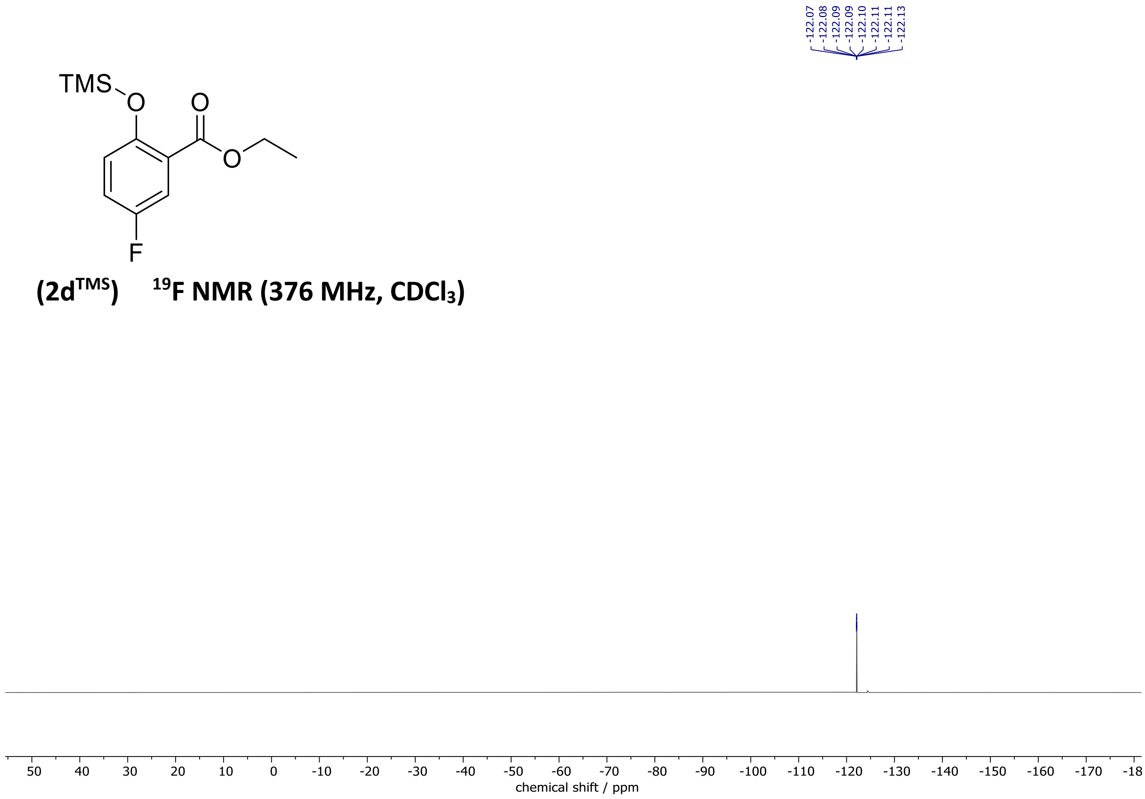

(2d[TMS]) ^{19}F NMR (376 MHz, $CDCl_3$)

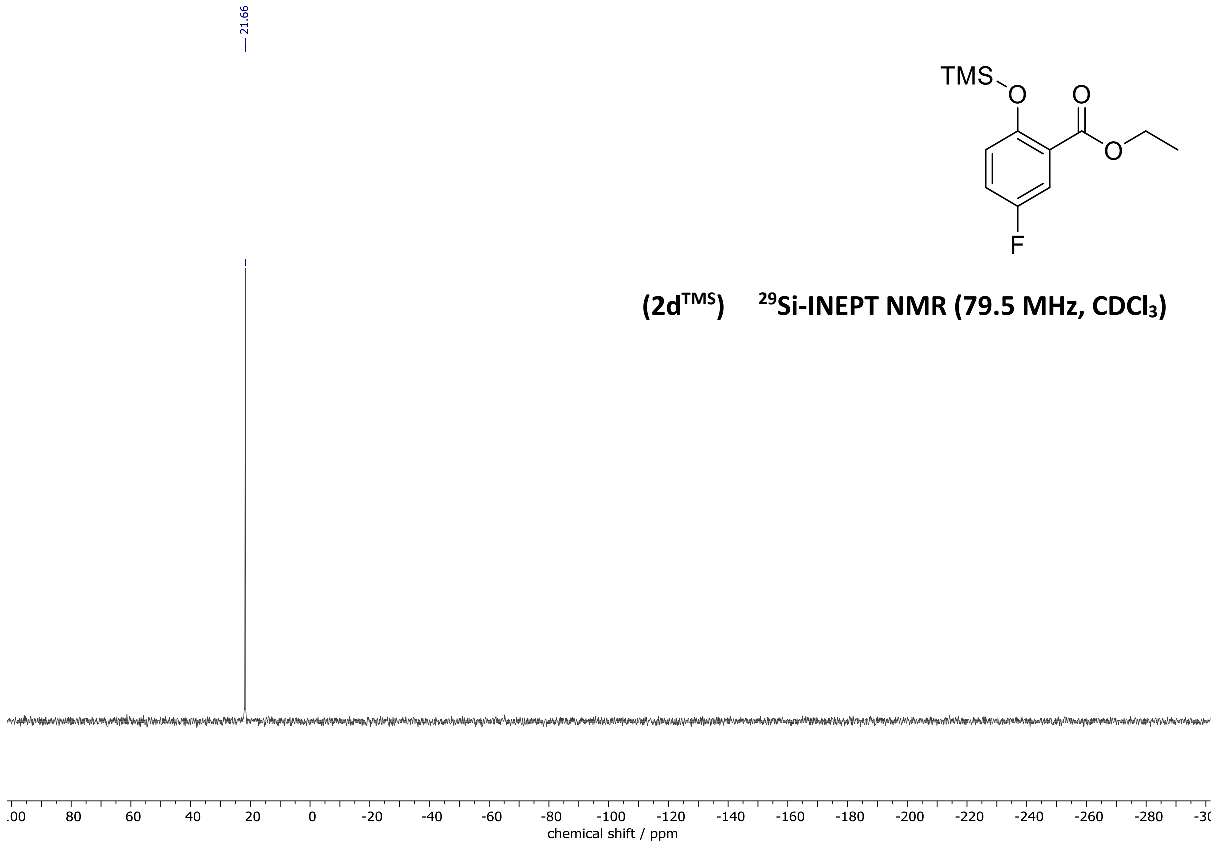

(2d^{TMS}) ^{29}Si-INEPT NMR (79.5 MHz, $CDCl_3$)

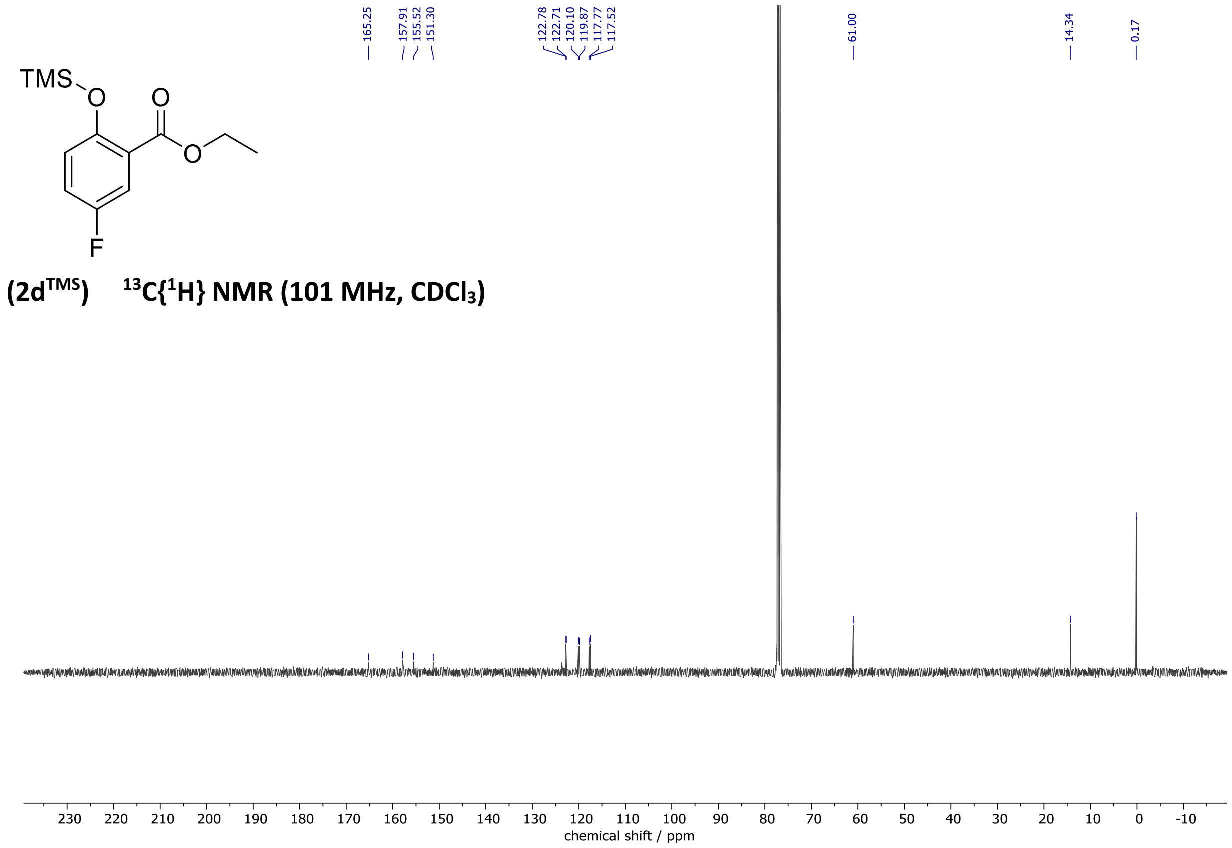

(2d^{TMS}) ^{13}C{^{1}H} NMR (101 MHz, $CDCl_3$)

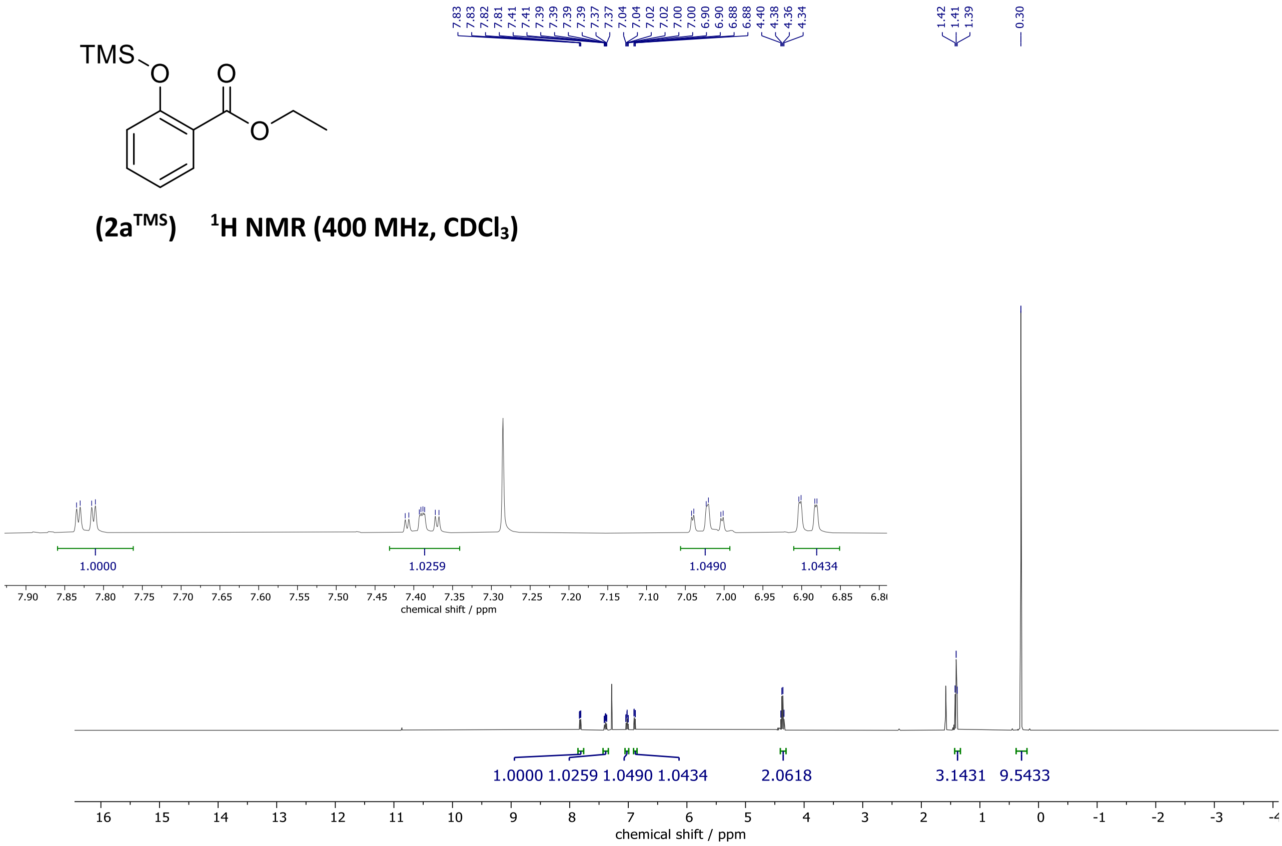

(2a^TMS) ^{1}H NMR (400 MHz, CDCl$_3$)

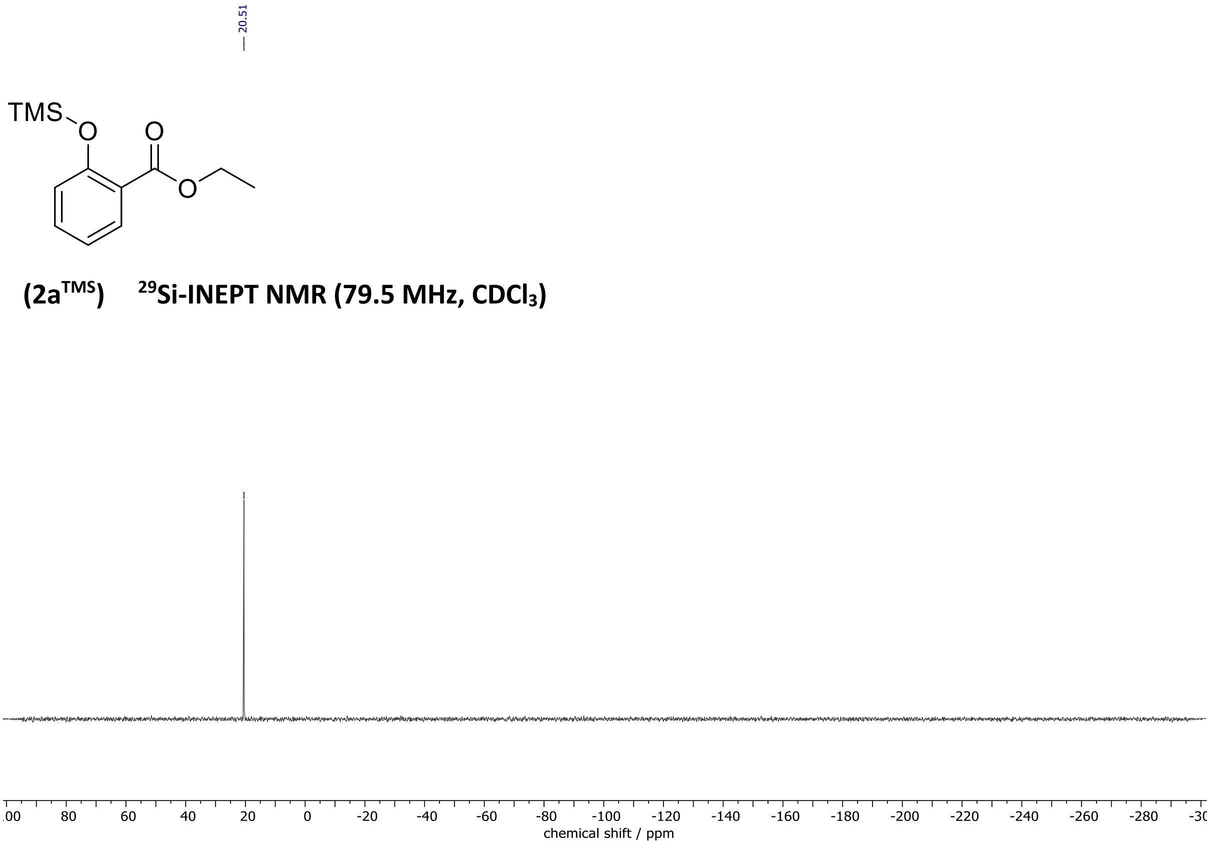

(2a^TMS) ^{29}Si-INEPT NMR (79.5 MHz, CDCl$_3$)

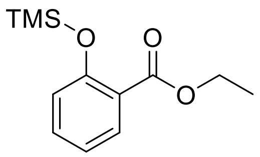

(2a^{TMS}) $^{13}C\{^1H\}$ NMR (101 MHz, $CDCl_3$)

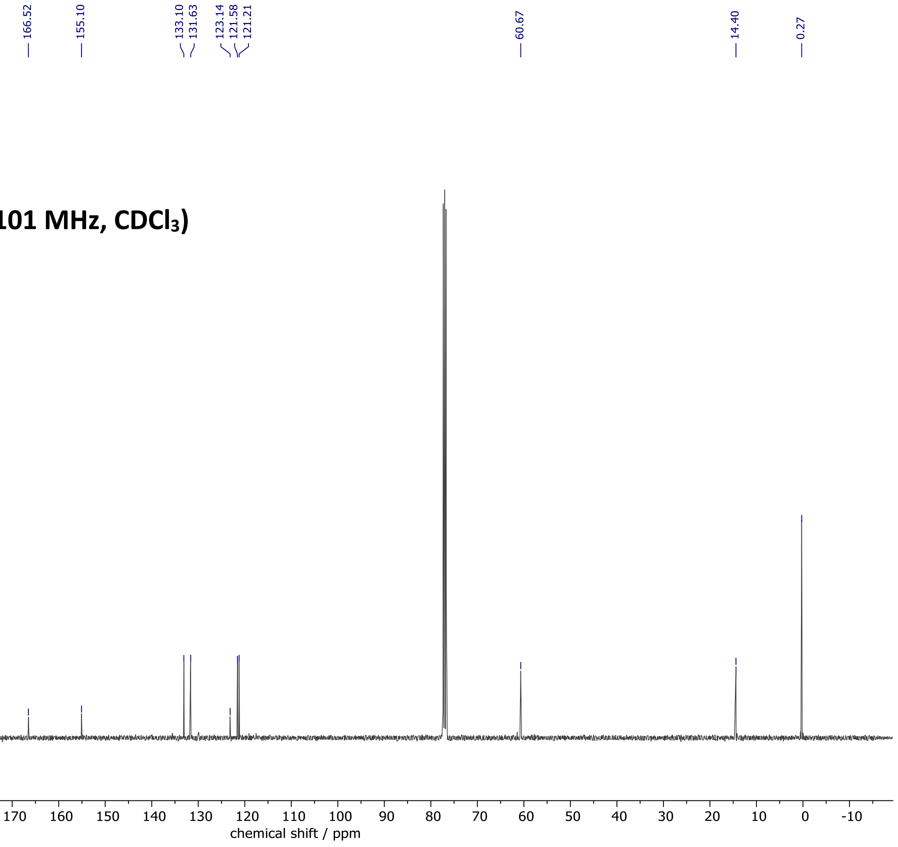

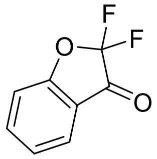

(4a) ^1H NMR (400 MHz, $CDCl_3$)

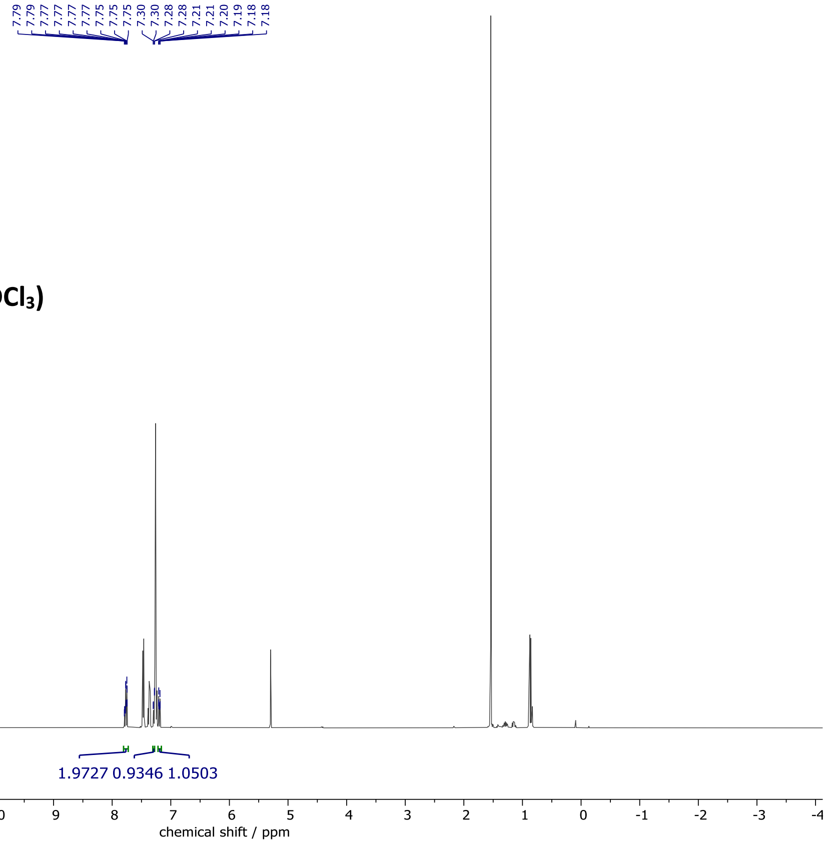

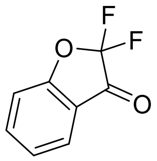

(4a) ^{19}F NMR (376 MHz, $CDCl_3$)

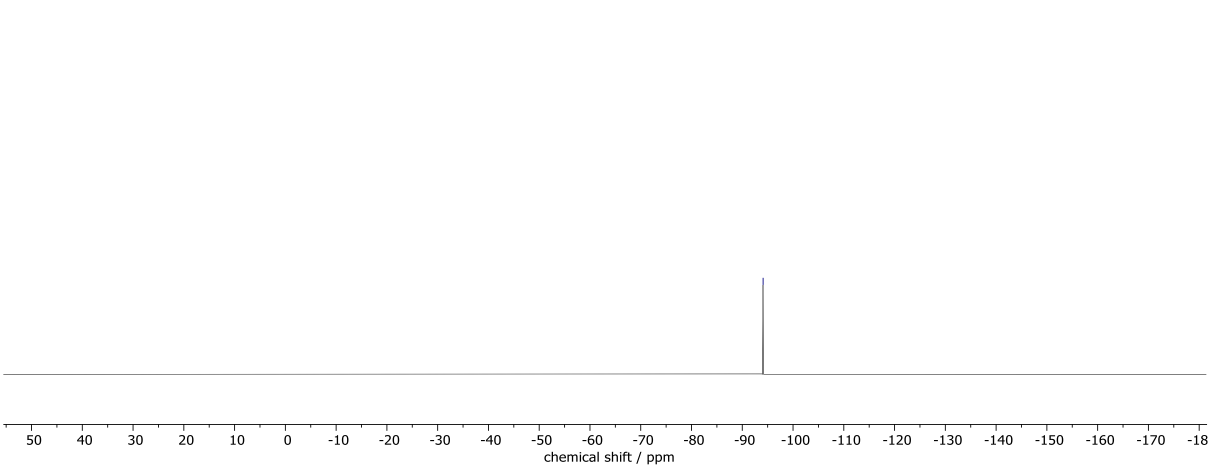

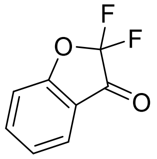

(4a) $^{13}C\{^1H\}$ NMR (101 MHz, $CDCl_3$)

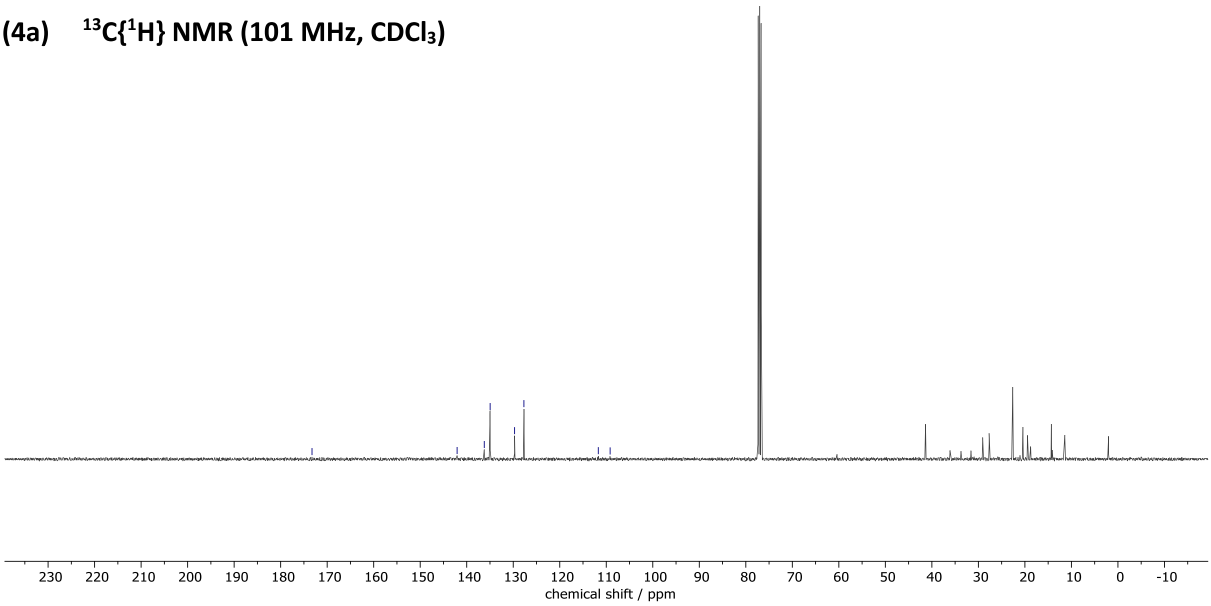

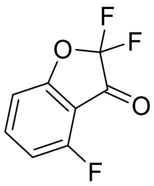

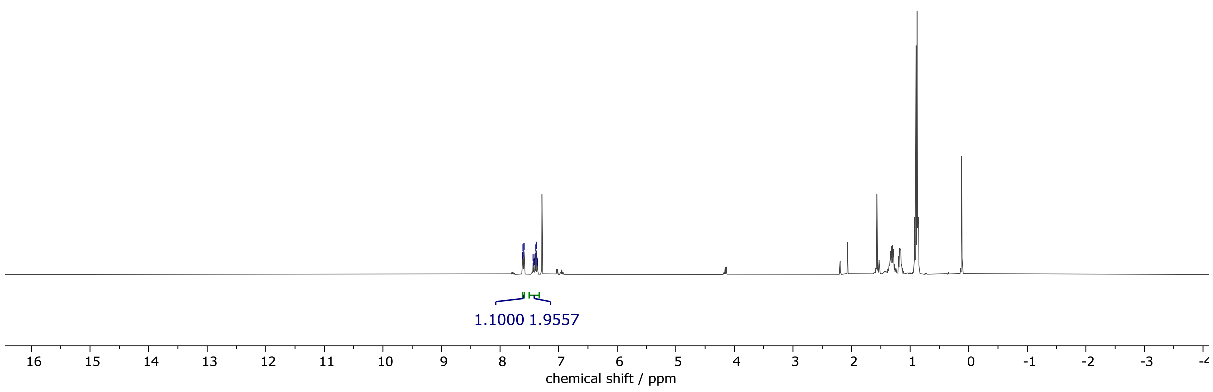

(4e) ^{1}H NMR (400 MHz, $CDCl_3$)

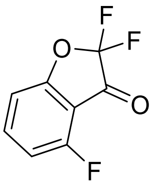

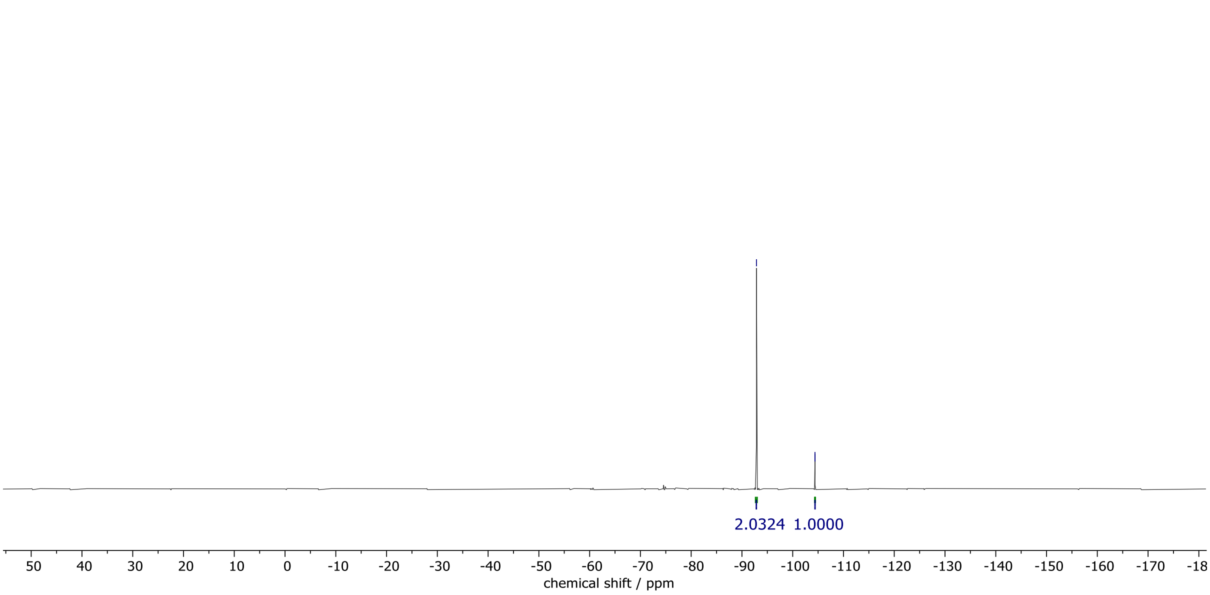

(4e) ^{19}F NMR (376 MHz, $CDCl_3$)

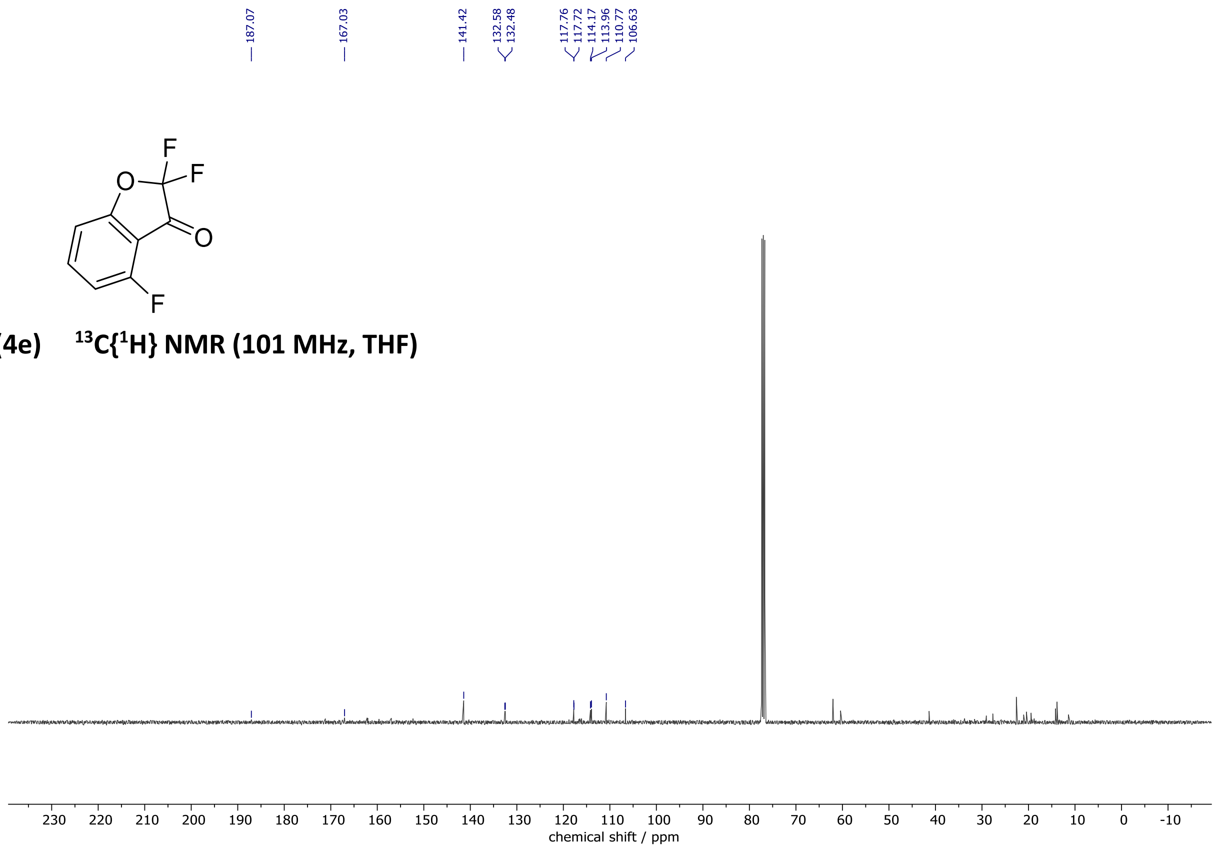

(4e) $^{13}C\{^{1}H\}$ NMR (101 MHz, THF)

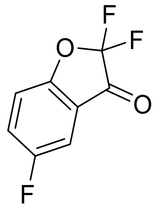

(4d) ^{1}H NMR (400 MHz, $CDCl_3$)

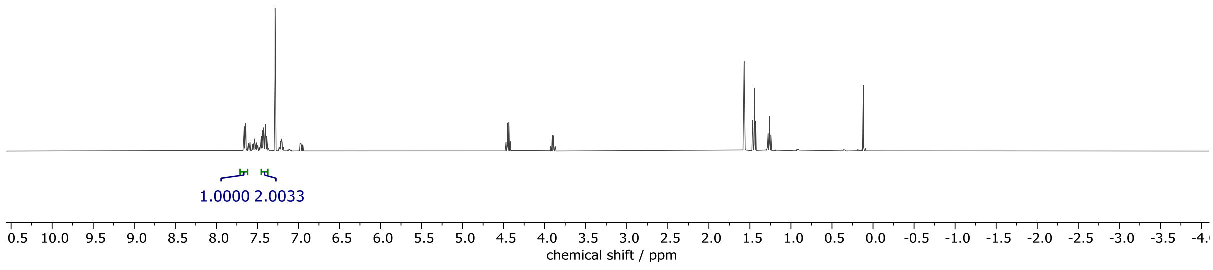

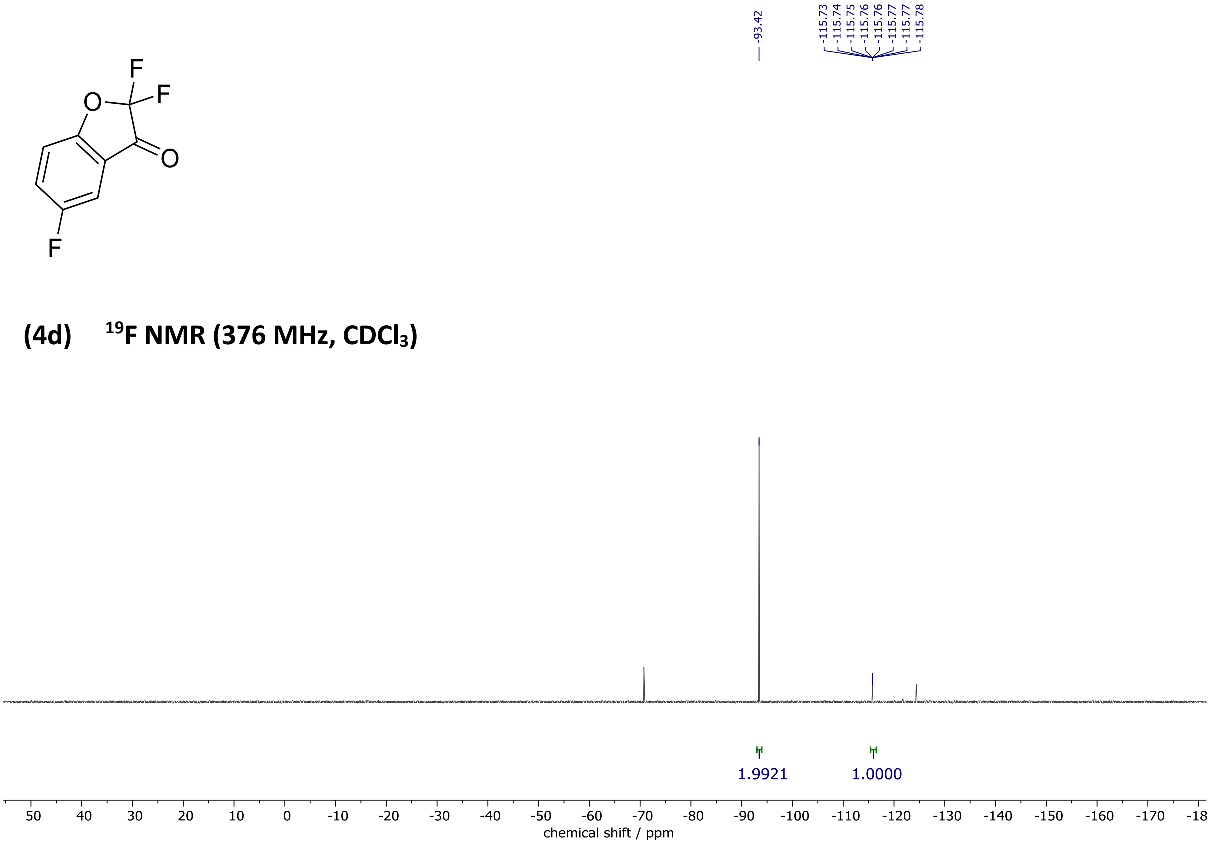

(4d) ^{19}F NMR (376 MHz, $CDCl_3$)

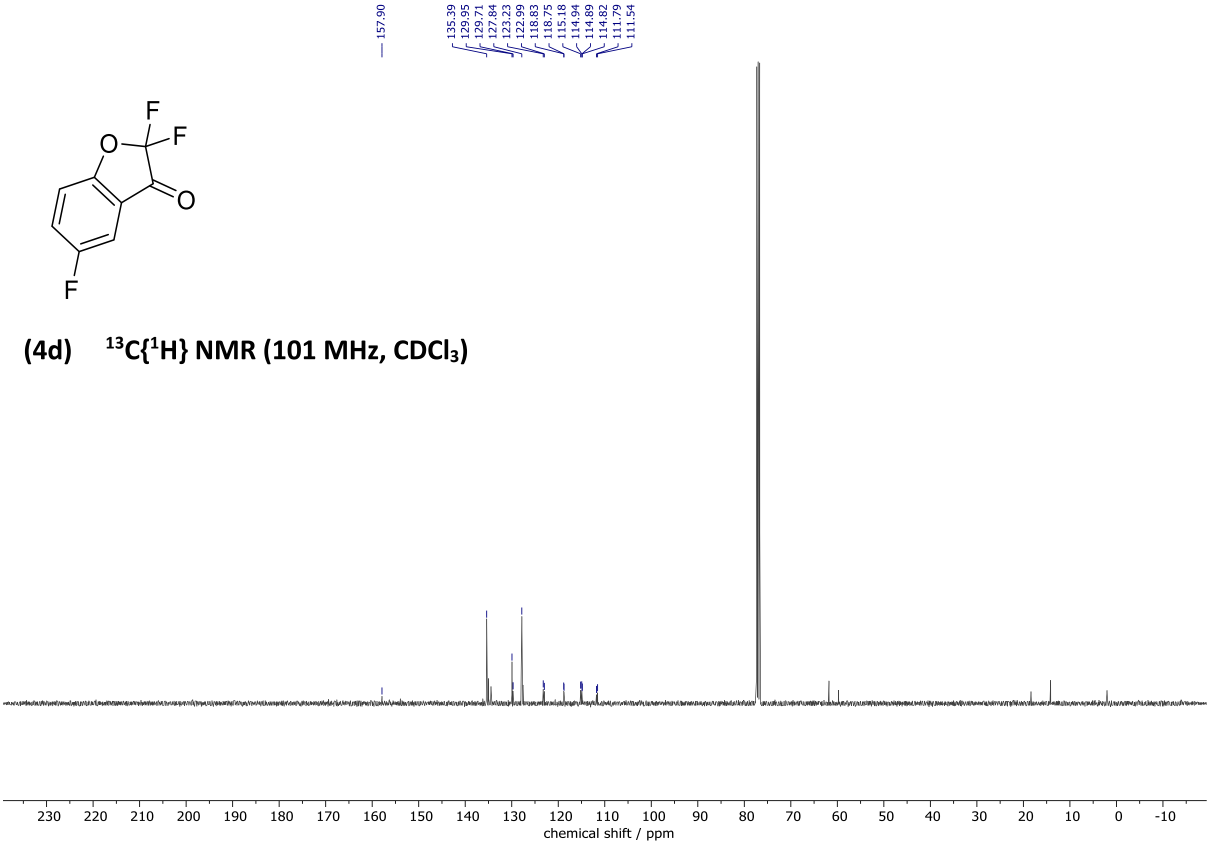

(4d) $^{13}C\{^1H\}$ NMR (101 MHz, $CDCl_3$)

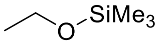

(EtOTMS) ^{1}H NMR (400 MHz, THF, solvent suppressed)

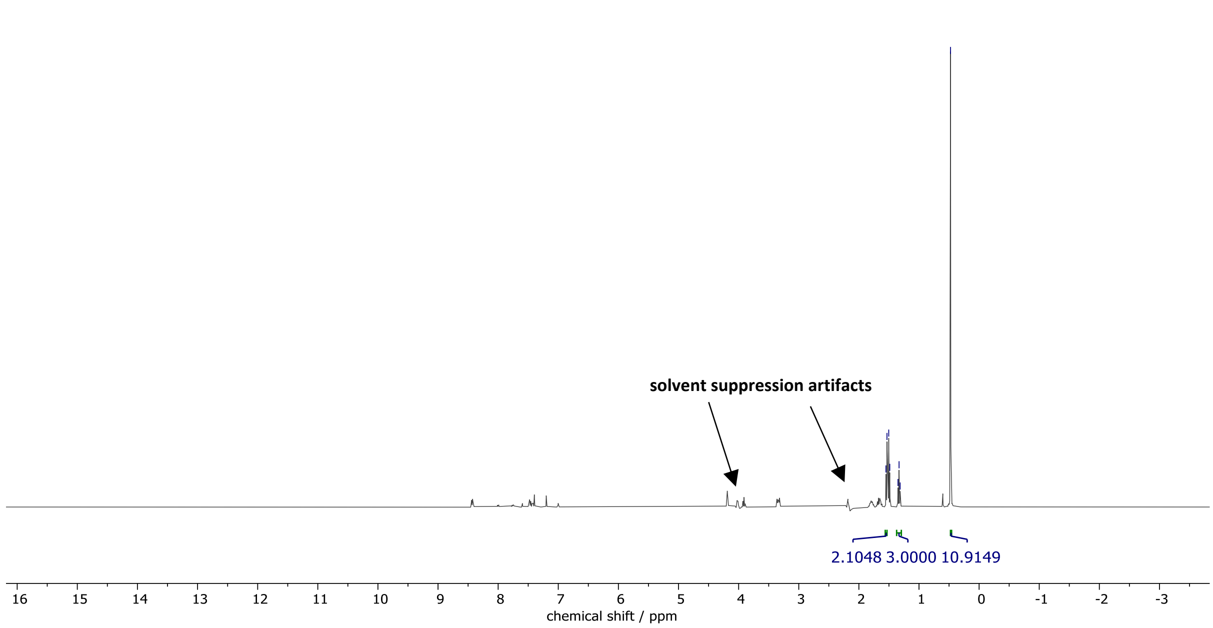

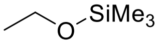

(EtOTMS) ^{29}Si-INEPT NMR (79.5 MHz, THF)

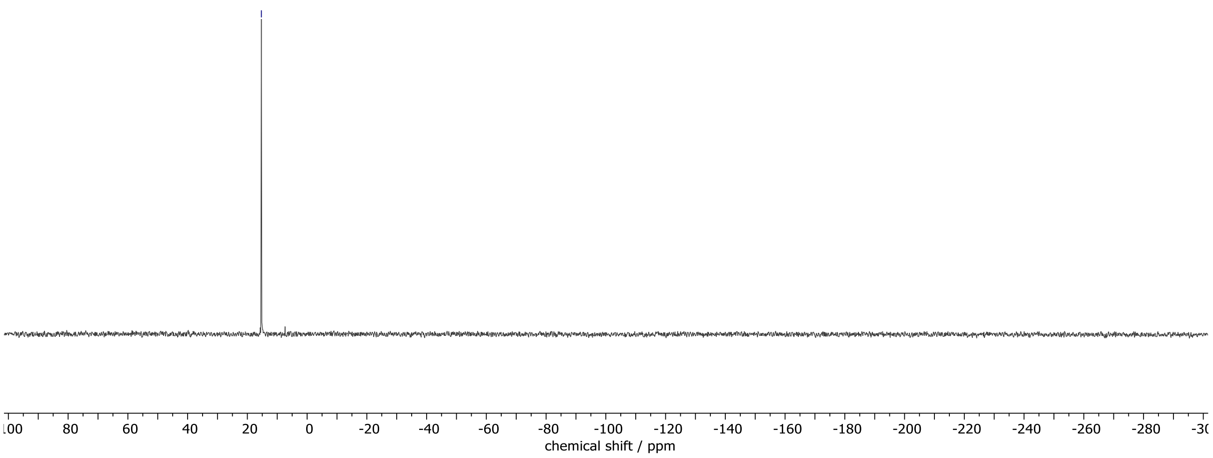

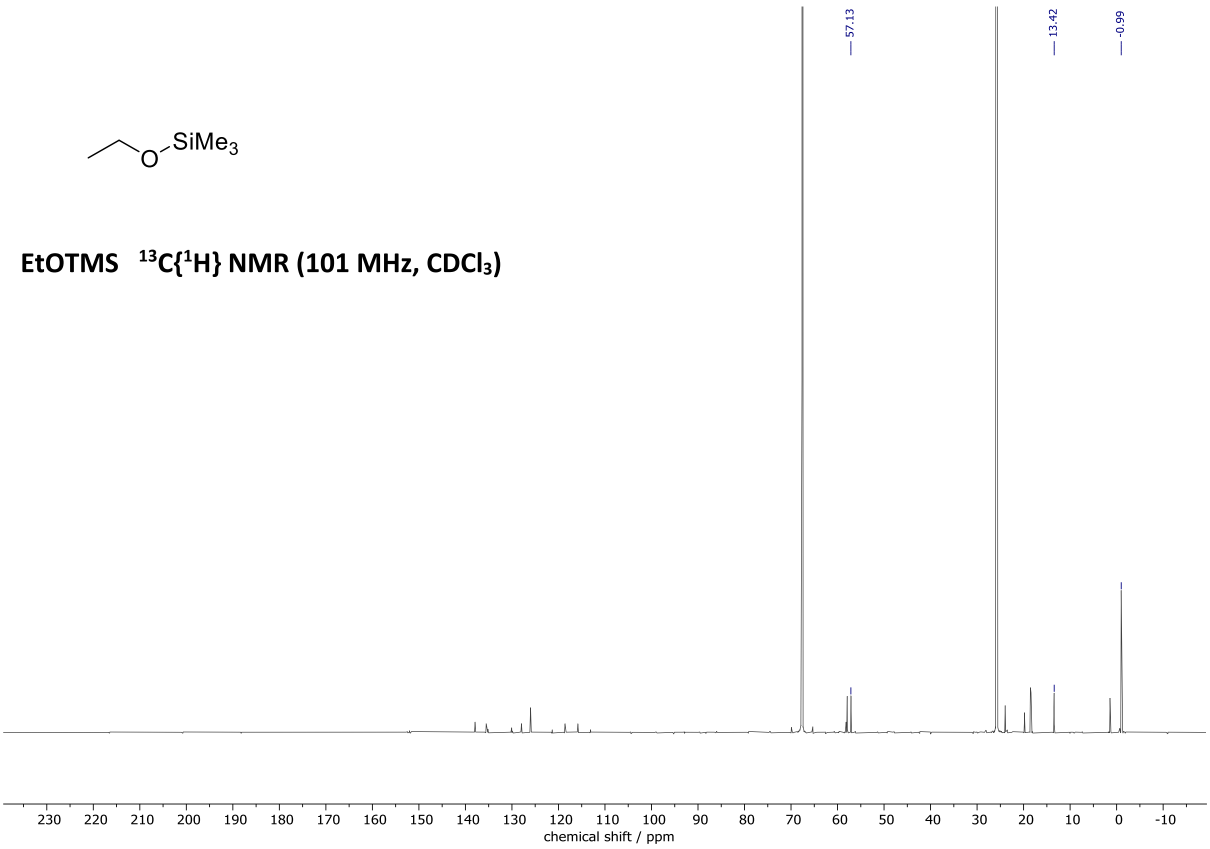
SiMe3
O
EtOTMS 13C{1H} NMR (101 MHz, CDCl3)
57.13
13.42
-0.99
230 220 210 200 190 180 170 160 150 140 130 120 110 100 90 80 70 60 50 40 30 20 10 0 -10
chemical shift / ppm

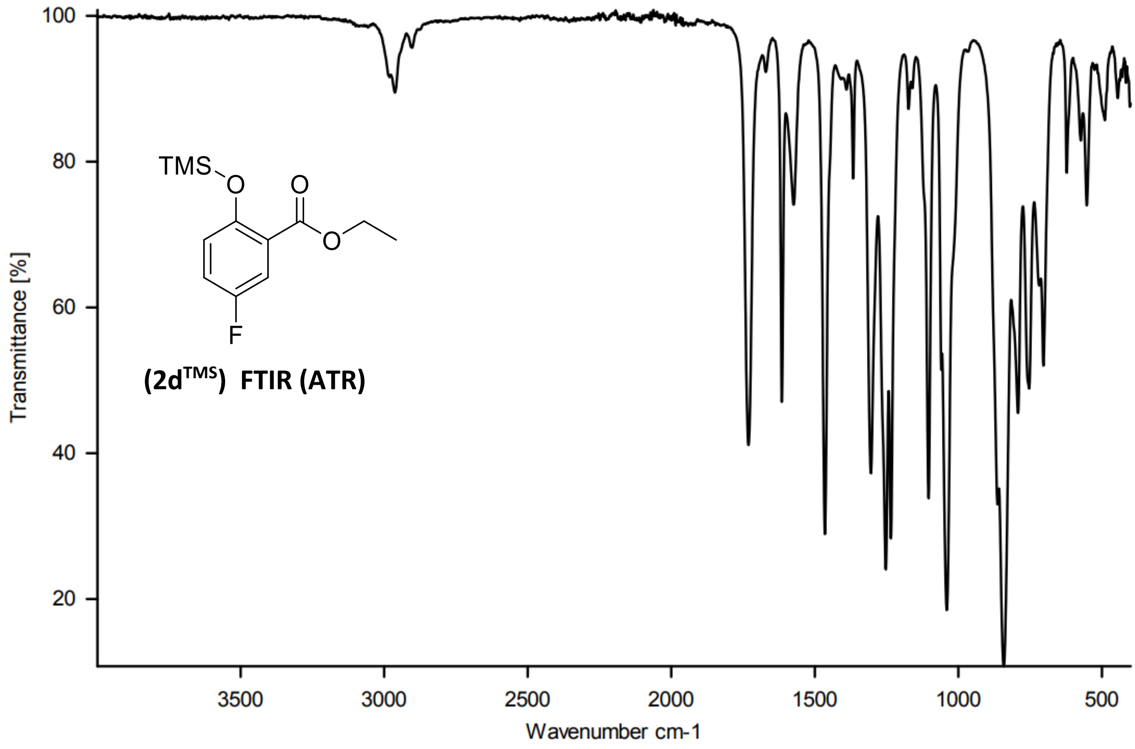
TMS
O
O
O
F
(2dTMS) FTIR (ATR)
Transmittance [%]
100
80
60
40
20
3500
3000
2500
2000
1500
1000
500
Wavenumber cm-1

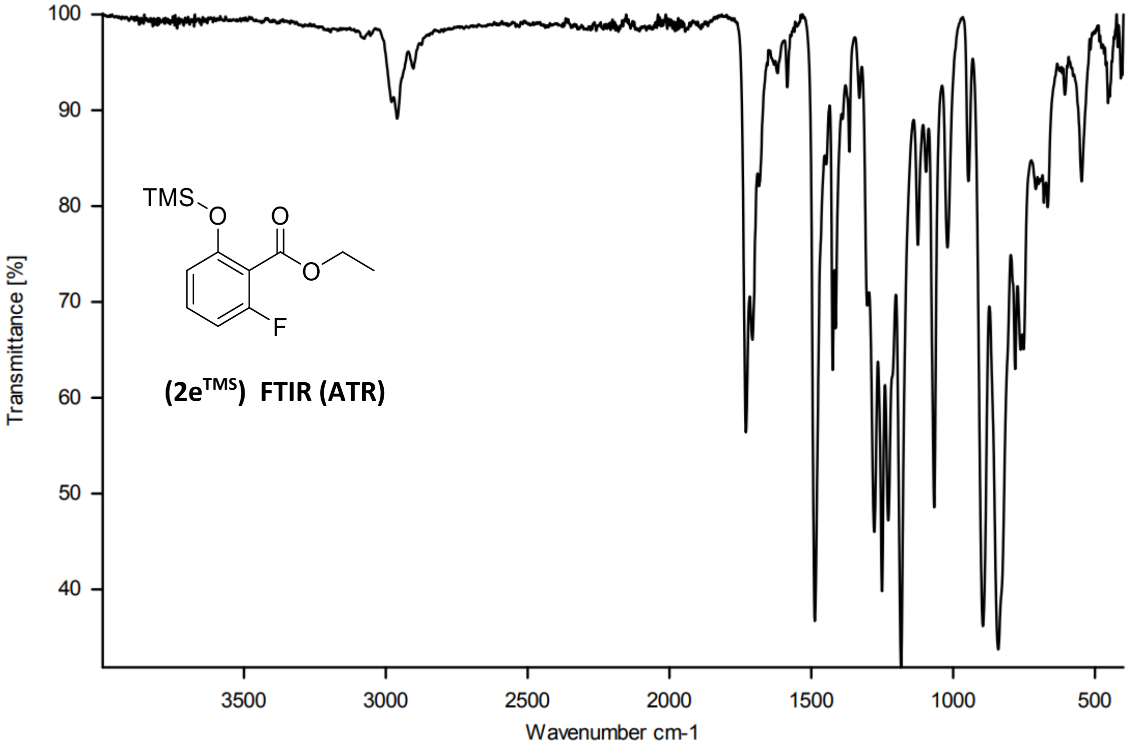
100
90
80
70
60
50
40
Transmittance [%]
TMS
O
O
O
F
(2eTMS) FTIR (ATR)
3500
3000
2500
2000
1500
1000
500
Wavenumber cm-1

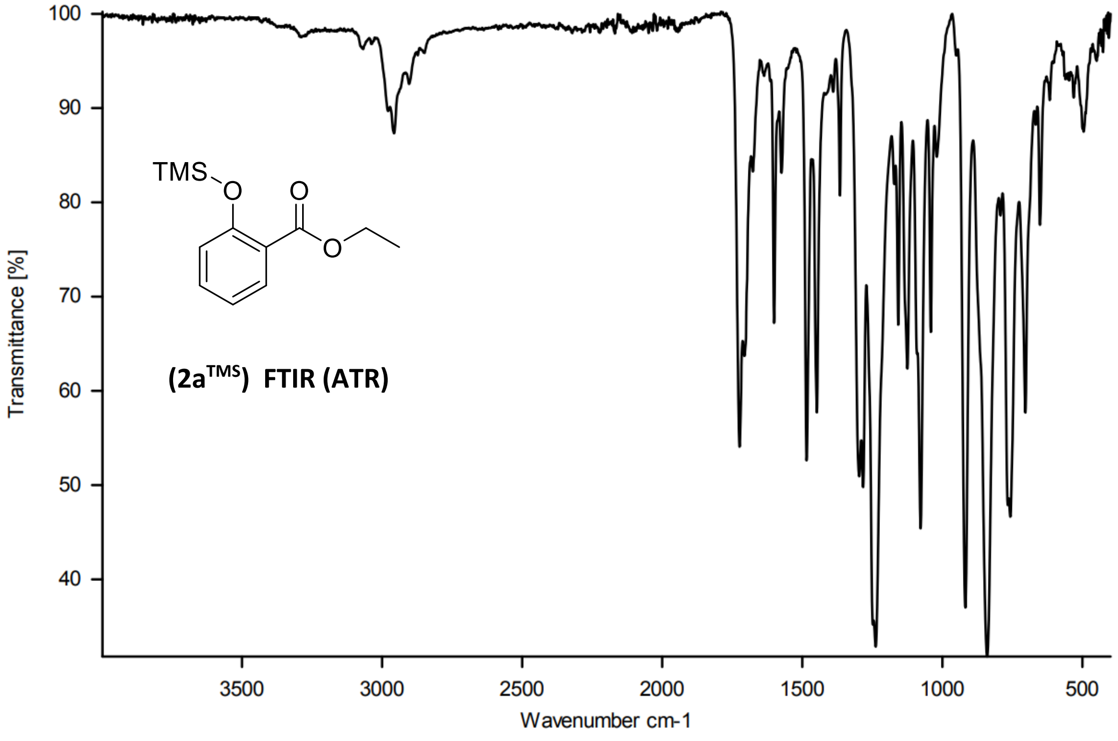
100
90
80
70
60
50
40
Transmittance [%]
TMS
O
O
O
(2aTMS) FTIR (ATR)
3500
3000
2500
2000
1500
1000
500
Wavenumber cm-1

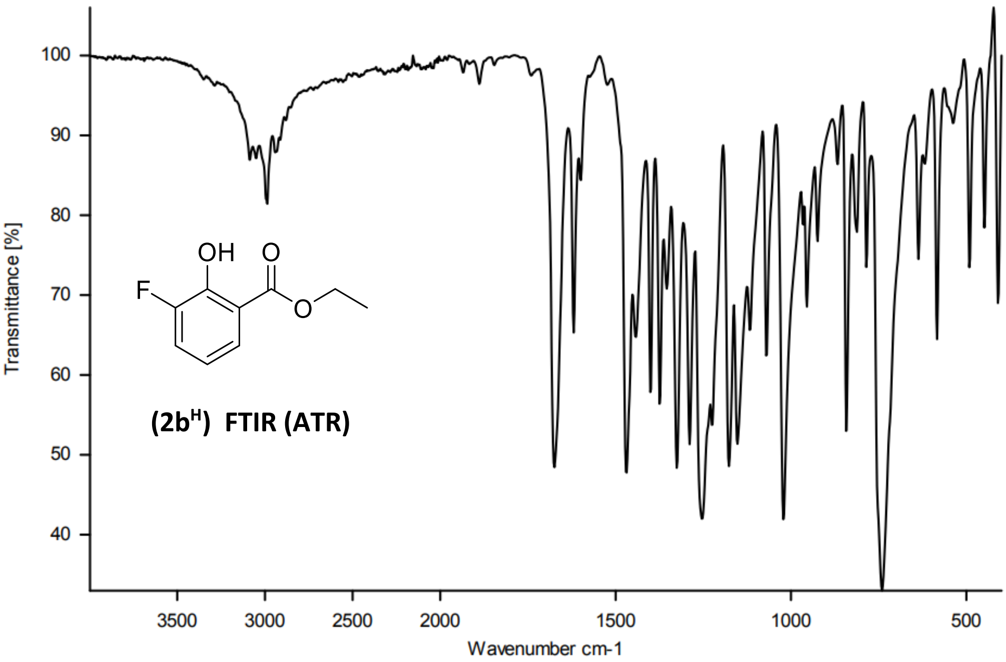

100
90
80
70
60
50
40
Transmittance [%]
3500
3000
2500
2000
1500
1000
500
Wavenumber cm-1
OH
O
F
O

(2b^{H}) FTIR (ATR)

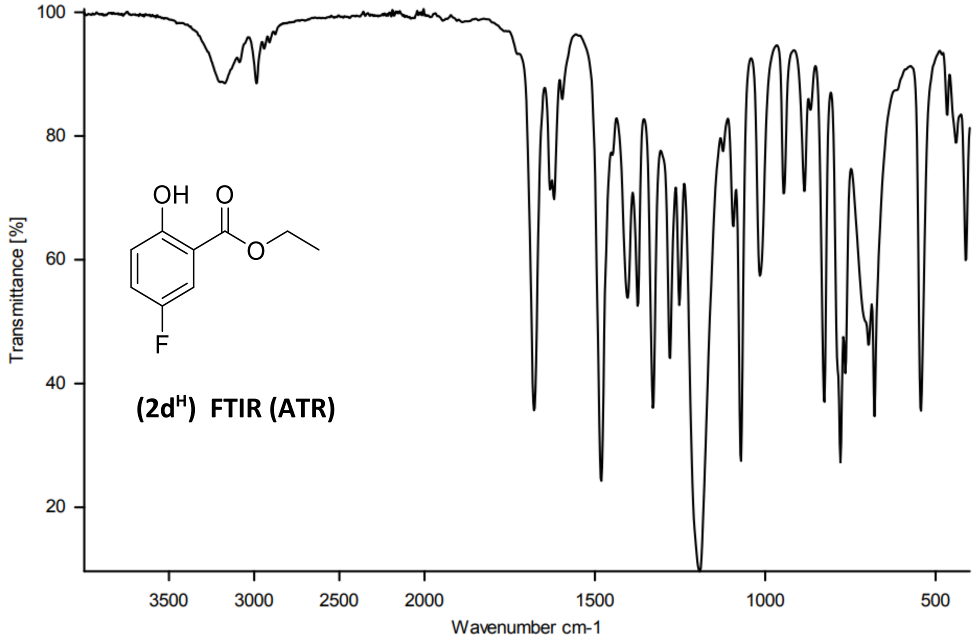

(2d^H) FTIR (ATR)
OH
O
O
F
Transmittance [%]
Wavenumber cm-1
100
80
60
40
20
3500
3000
2500
2000
1500
1000
500

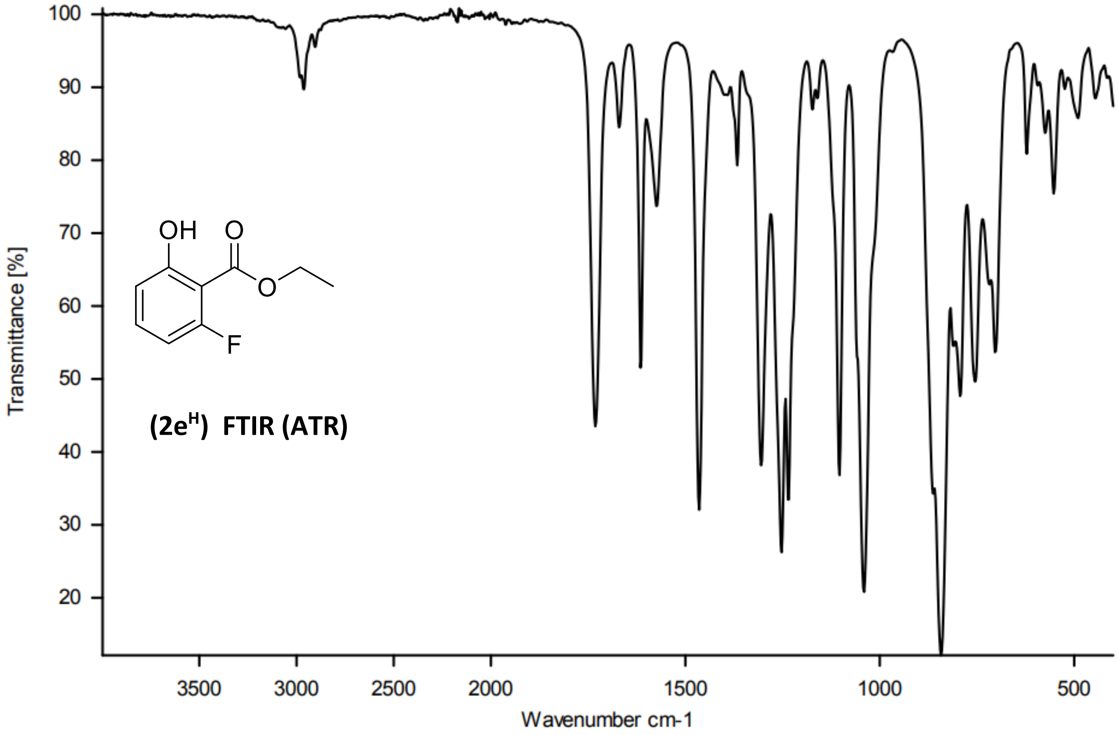

(2e^H) FTIR (ATR)
OH
O
O
F
Transmittance [%]
Wavenumber cm-1
100
90
80
70
60
50
40
30
20
3500
3000
2500
2000
1500
1000
500

4

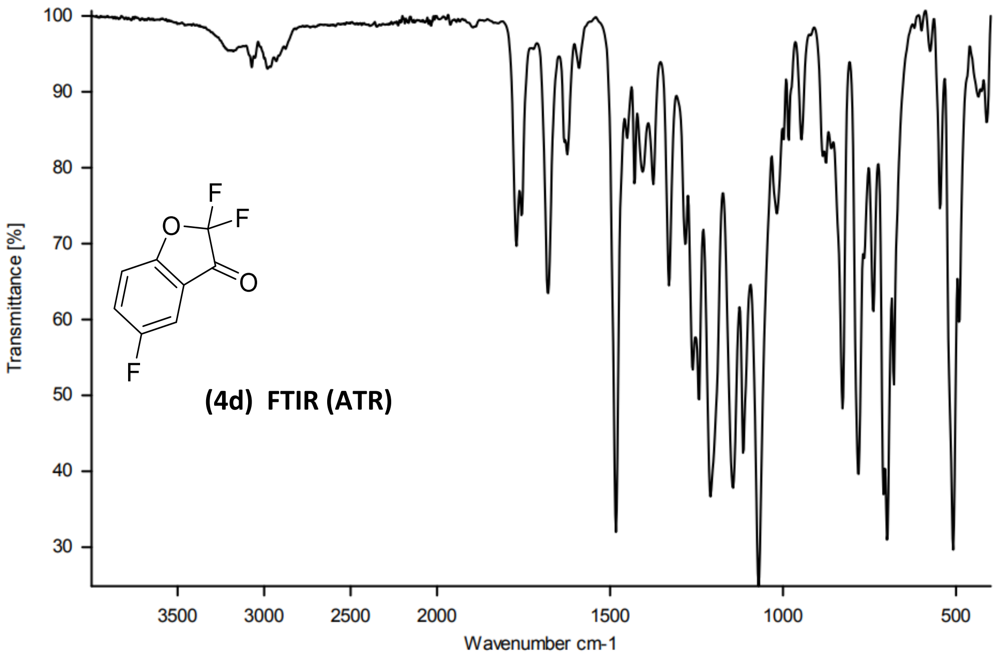

Transmittance [%]
Wavenumber cm-1
(4d) FTIR (ATR)

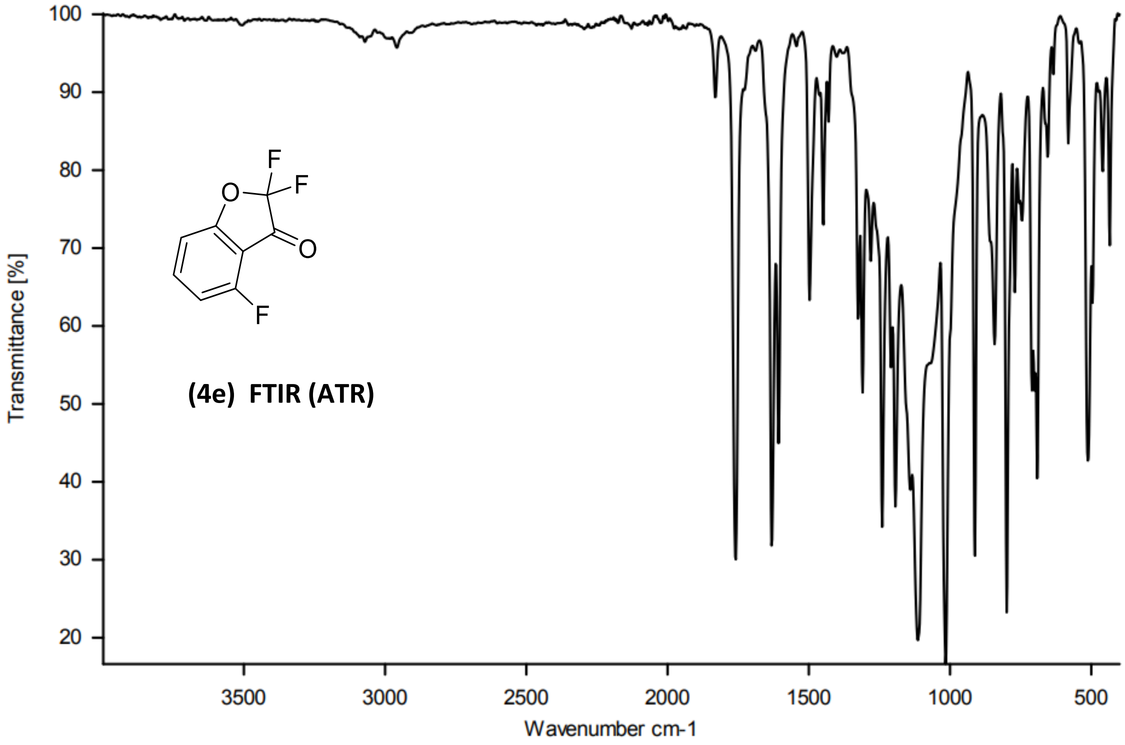

Transmittance [%]
Wavenumber cm-1
(4e) FTIR (ATR)
